# Supplementary figures and images for: MICU2 up-regulation enhances tumor aggressiveness and metabolic reprogramming during colorectal cancer development
Source: PLoS Biol. 2024 Oct 28;22(10):e3002854. doi: 10.1371/journal.pbio.3002854 (PMC11542858; doi:10.1371/journal.pbio.3002854)

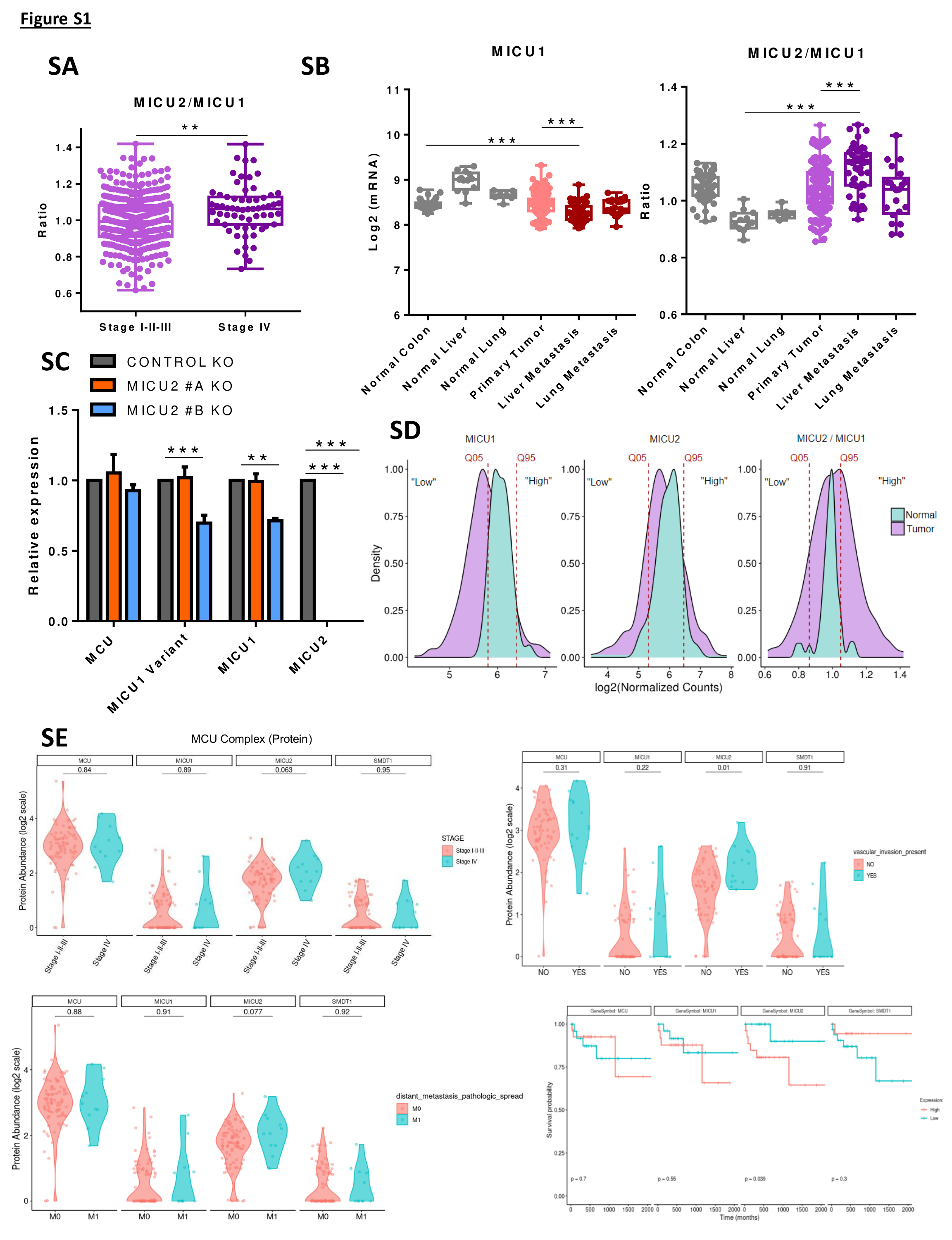

Supplement: S1 Fig — (A) Transcriptomic analysis of the MICU2/MICU1 ratio according to CRC stages in the TCGA-COAD (B) Transcriptomic analysis of the MICU1 and MICU2/MICU1 ratio in the GSE41258 data sets. Each data point represents an individual sample (ANOVA followed by Dunn’s multiple comparisons test). (C) Bar plot representing the expression of MCU, MICU1, MICU1 variant, and MICU2 mRNAs measured by RT-qPCR in the HCT116 Control and MICU2 KO cell lines (n = 3, ANOVA followed Dunnett’s multiple comparisons test, **p < 0.01 and ***p < 0.001). (D) Density plots representing the definition of the status of MICU1, MICU2, and the MICU2/MICU1 ratio of primary colon tumor samples of the TCGA-COAD data set. (E) Proteomics analysis generated by CPTAC consortium of 95 tumor samples included into the TCGA-COAD and TCGA-READ genomic projects. (Upper left) Violin plots representing the relative protein abundance of MCU, MICU1, MICU2, and EMRE (SMDT1) in tumor samples in function of AJCC pathological stages (Stages I-II-III compared to Stage IV, upper left), of the presence of a vascular invasion (upper right), of the presence of distant metastasis (lower left). Kaplan–Meier survival plot representing the overall survival probability of patients according the levels of expression of MCU, MICU1, MICU2, and EMRE (SMDT1). High and Low groups have been determined based on the median of protein abundance of individual protein in tumor samples. The data underlying the graphs shown in the figure can be found in S1 and S2 Data. (TIF) [file pbio.3002854.s001.tif]

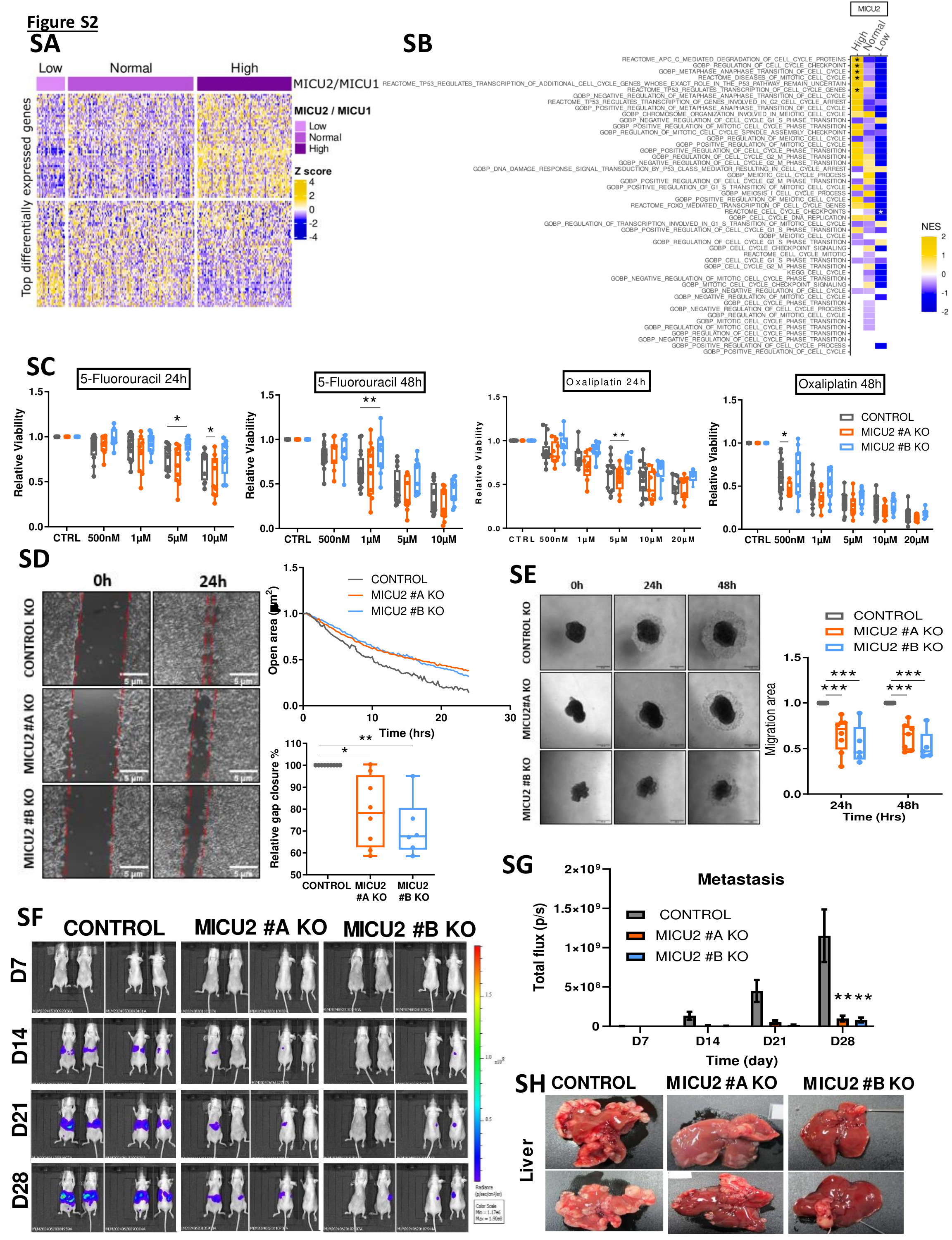

Supplement: S2 Fig — (A) Heatmap of the most differentially expressed genes between colon tumor samples in the TCGA-COAD data set with low, normal, or high expression of MICU2/MICU1 ratio. (B) Heatmap of normalized enrichment scores of all cell cycle–associated genes obtained by GSEA for primary colon tumor samples with low, normal, or high expression of MICU2. A star indicates an adjusted p-value <0.05. (C) Boxplots representing the viability of the Control and MICU2 KO cell lines cultured in the absence of presence in 5FU (500 nM, 1 μm, 5 μm, and 10 μm) (top panel) and oxaliplatin (500 nM, 1 μm, 5 μm, 10 μm, and 20 μm) (bottom panel) for 24 and 48 h (n = 9–11). (D) Left panel: representative images of the wound healing assay. Right panel: curve and boxplots representing the percentage of gap closure as a function of time and after 24 h in the Control and MICU2 KO cell lines. The scale bar is 5 μm (n = 6–8, Kruskal–Wallis test). (E) Left panel: representative images of 3D spheroids of the Control and MICU2 KO cell lines plated on top of a fibronectin layer after 0, 24, and 48 h. Right panel: boxplot representing the migration area at 24 and 48 h. The scale bar is 20 μm (n = 5–8, Kruskal–Wallis test). (F) Representative bioluminescence images of cancer metastasis in mice injected with the luciferase-expressing Control or MICU2 KO cells lines. (G) Quantification of number of photons/s per mouse at indicated days (n = 5–8, Kruskal–Wallis test). (H) Representative image showing the formation of metastatic nodules in the liver of mice injected with HCT116 WT or MICU2 KO cells. On all plots, *p < 0.05, **p < 0.01, and ***p < 0.001. The data underlying the graphs shown in the figure can be found in S1, S2 and S4 Datas. (TIF) [file pbio.3002854.s002.tif]

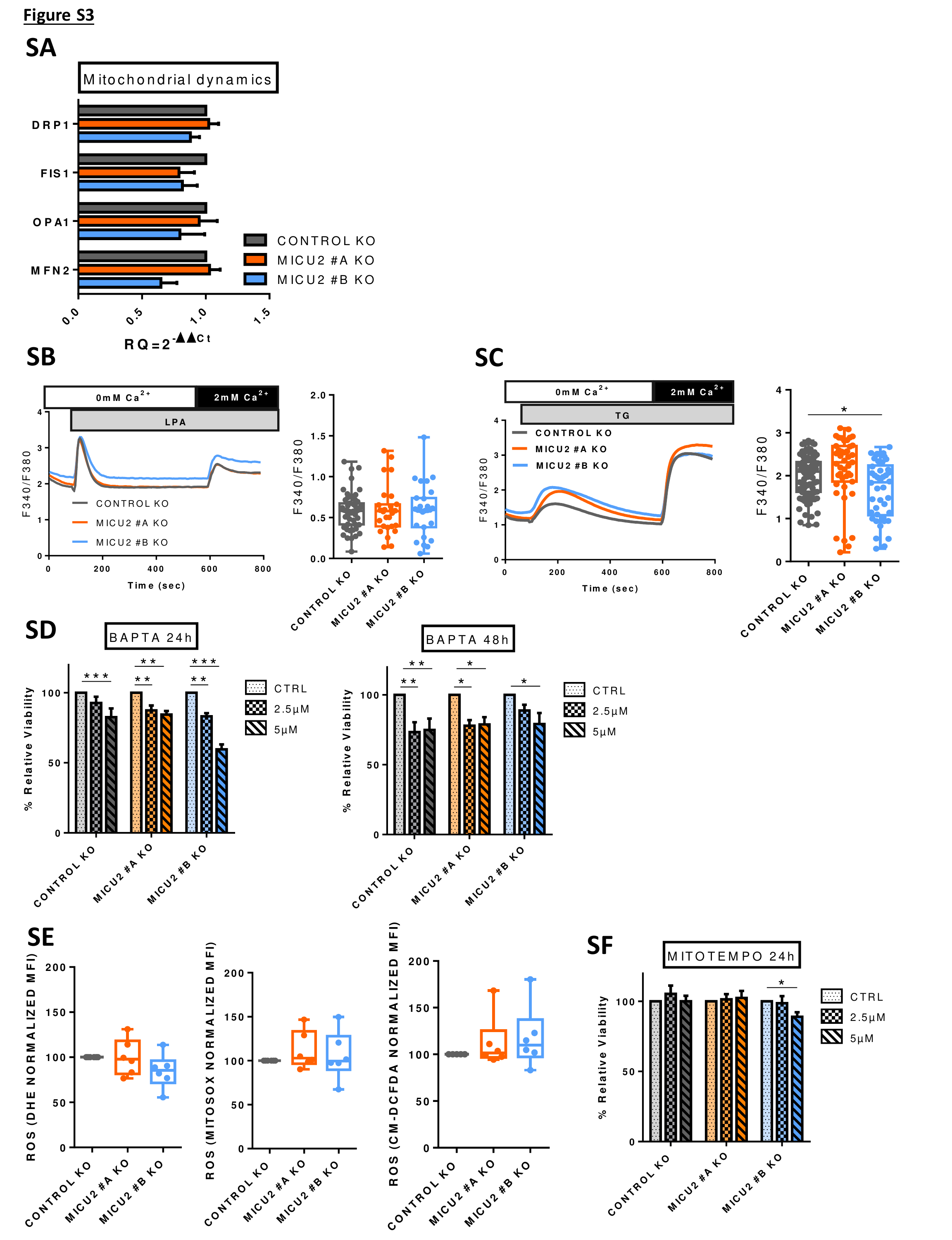

Supplement: S3 Fig — (A) Bar plots representing the fold change of the expression of genes associated with mitochondrial dynamics in the Control and MICU2 KO cell lines compared with the Control KO cell line (n = 3–5). (SB) Left panel: representative measurements of cytosolic [Ca2+] in the Control and MICU2 KO cell lines measured with Fura-2 AM in response to 1 μm of LPA applied in the absence of extracellular Ca2+ and in the presence of 2 mM of extracellular Ca2+. Right panel: boxplot representing the cytosolic Fura-2AM 340/380 ratio measured in the presence of extracellular Ca2+ in the Control and MICU2 KO cell lines (n = 4, N = 27–55). (C) Left panel: representative measurements of cytosolic [Ca2+] in the Control and MICU2 KO cell lines measured with Fura-2AM in response to 2 μm TG applied in the absence of extracellular Ca2+ and in the presence of 2 mM of extracellular Ca2+. Right panel: boxplot representing the basal Fura-2AM 340/380 ratio measured in the presence of extracellular Ca2+ in the Control and MICU2 KO cell lines (n = 6, N = 42–95, ANOVA followed by Dunnett’s multiple comparisons test). (D) Bar plots representing the viability of the Control and MICU2 KO cell lines cultured 24 or 48 h in presence or absence of BAPTA-AM (a Ca2+ chelator) (n = 3–5, ANOVA followed by Dunnett’s multiple comparisons test). (E) Boxplots representing ROS production in the Control and MICU2 KO cell lines. Total ROS, mitochondrial ROS, and hydrogen peroxide were measured using DHE, MitoSox, and DCFDA, respectively. (F) Bar plots representing the viability of the Control and MICU2 KO cell lines incubated for 24 h with MITOTEMPO (a mitochondrial ROS chelator) (n = 5–7, ANOVA followed by Dunnett’s multiple comparisons test). On all plots, *p < 0.05, **p < 0.01, and ***p < 0.001. The data underlying the graphs shown in the figure can be found in S1 Data. (TIF) [file pbio.3002854.s003.tif]

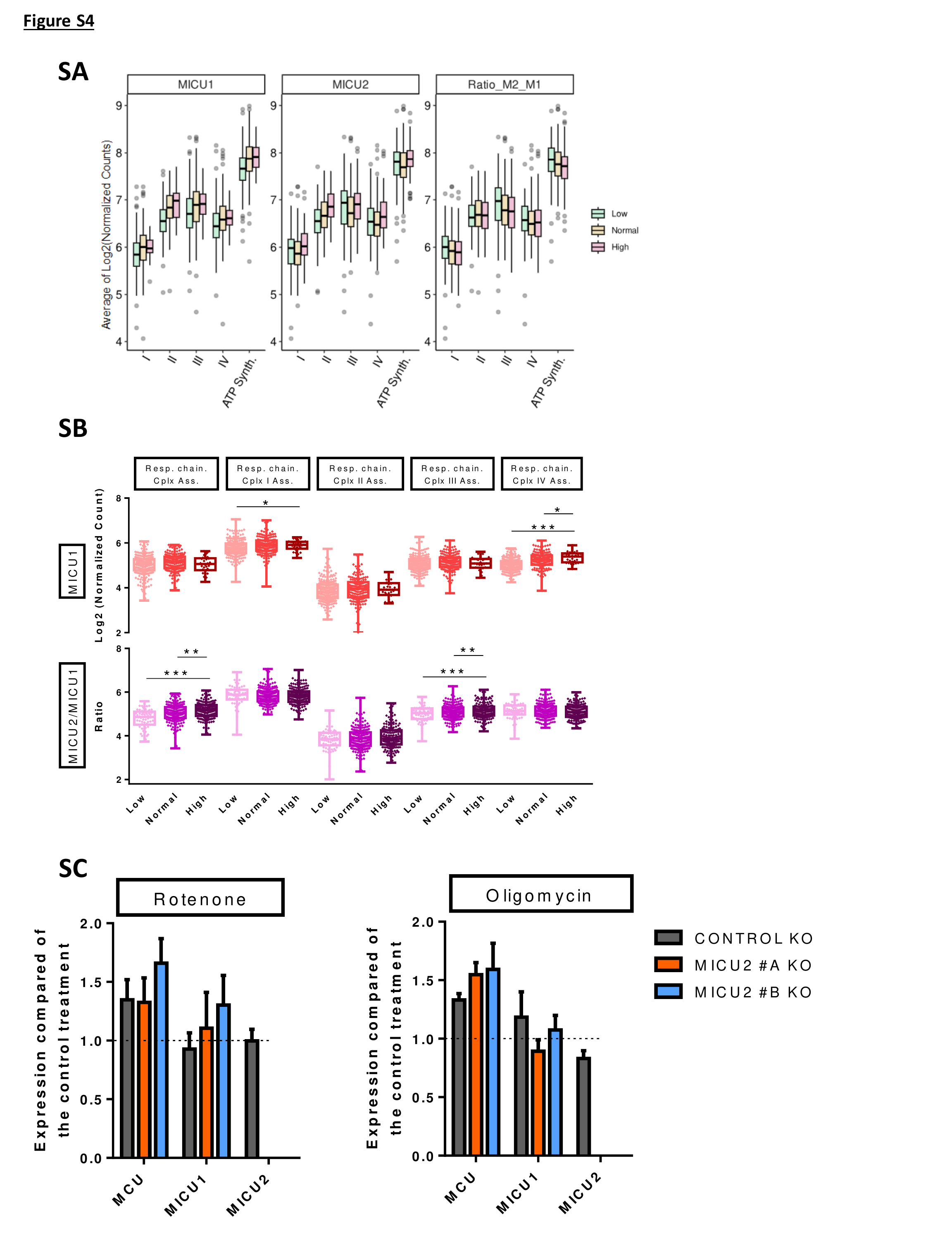

Supplement: S4 Fig — (A) TCGA analysis showing expression values of respiratory chain complexes as a function of low, normal, and high expression of MICU1, MICU2, and the MICU2/MICU1 ratio. (B) Boxplot representing the average expression of genes associated with the assembly of mitochondrial respiratory chain complexes I, II, III, and IV based on the status MICU1 and MICU2/MICU1 ratio in primary colon tumor samples from the TCGA-COAD data set. (C) The expression levels of MCU, MICU1, and MICU2 were determined in the Control KO and MICU2 KO cells treated with the inhibitors rotenone and oligomycin using RT-qPCR. The data are presented as the mean ± standard error of the mean of 4 independent experiments. On all plots, *p < 0.05, **p < 0.01, and ***p < 0.001. The data underlying the graphs shown in the figure can be found in S1 and S2 Datas. (TIF) [file pbio.3002854.s004.tif]

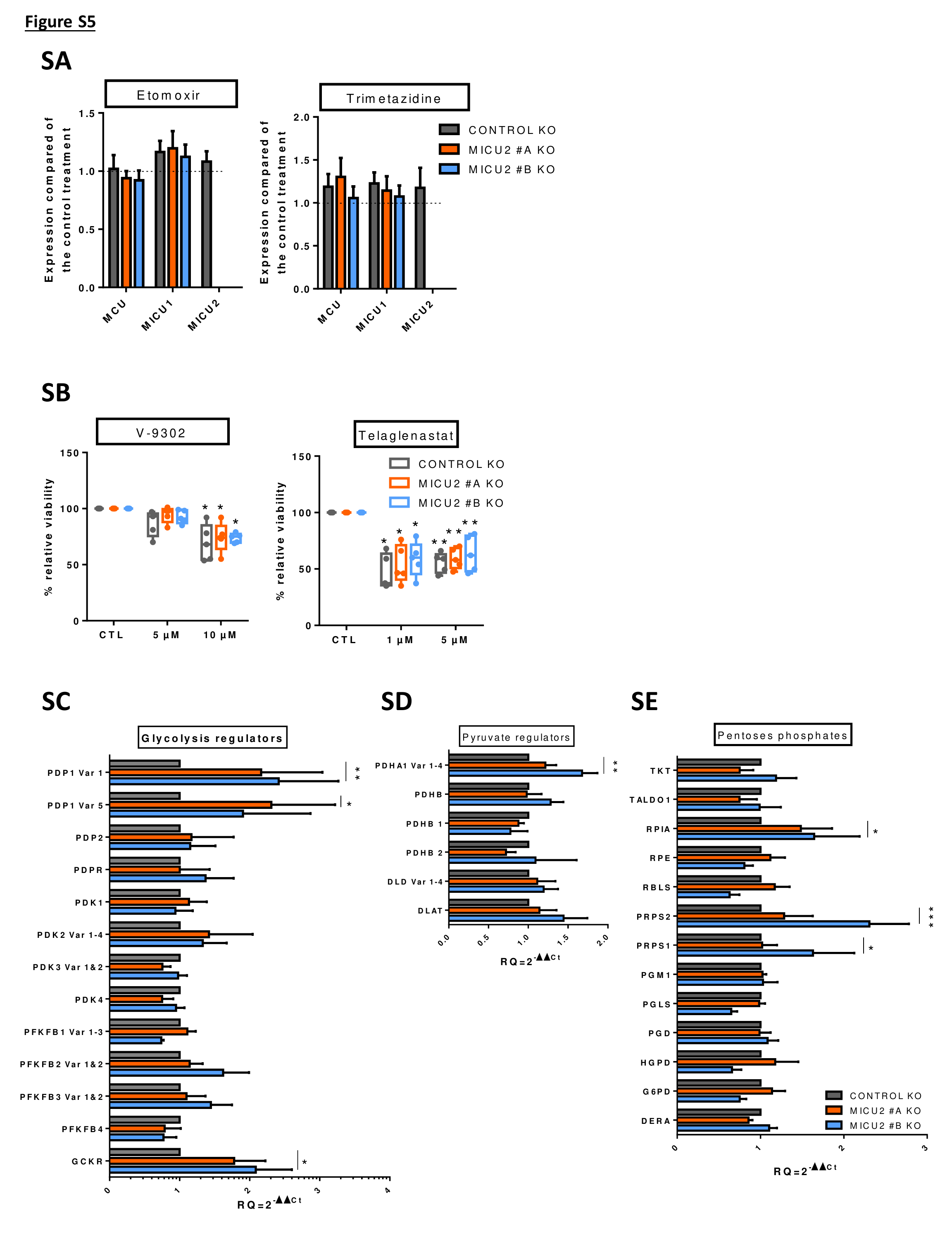

Supplement: S5 Fig — (A) The expression of MCU, MICU1, and MICU2 were determined in the Control and MICU2 KO cell lines treated with the inhibitors etomoxir (left panel) and trimetazidine (right panel) using RT-qPCR. The data are presented as the mean ± standard error of the mean of 3 or 4 independent experiments. (B) Efficacy of V-9302 (ASCT2 inhibitor) (left panel) and telaglenastat (glutaminase inhibitor) (right panel) treatments (1–10 μm) after 48 h on the Control and MICU2 KO cell lines (n = 5, ANOVA followed by Dunn’s multiple comparisons test, *p < 0.05). (C) qRT-PCR data showing fold changes in mRNA levels of glycolysis regulator proteins in the Control and MICU2 KO cell lines (n = 4–7, ANOVA followed by Dunnett’s multiple comparisons test, *p < 0.05 and **p < 0.01). (D) RT-qPCR data showing fold changes in mRNA levels of pyruvate regulator proteins in the Control and MICU2 KO cell lines (n = 3–5, ANOVA followed by Dunnett’s multiple comparisons test, **p < 0.01). (E) RT-qPCR data showing fold changes in mRNA levels of pentose phosphates proteins in the Control KO and MICU2 KO cell lines (N = 5, ANOVA followed by Dunnett’s multiple comparisons test, *p < 0.05 and ***p < 0.001). The data underlying the graphs shown in the figure can be found in S1 Data. (TIF) [file pbio.3002854.s005.tif]

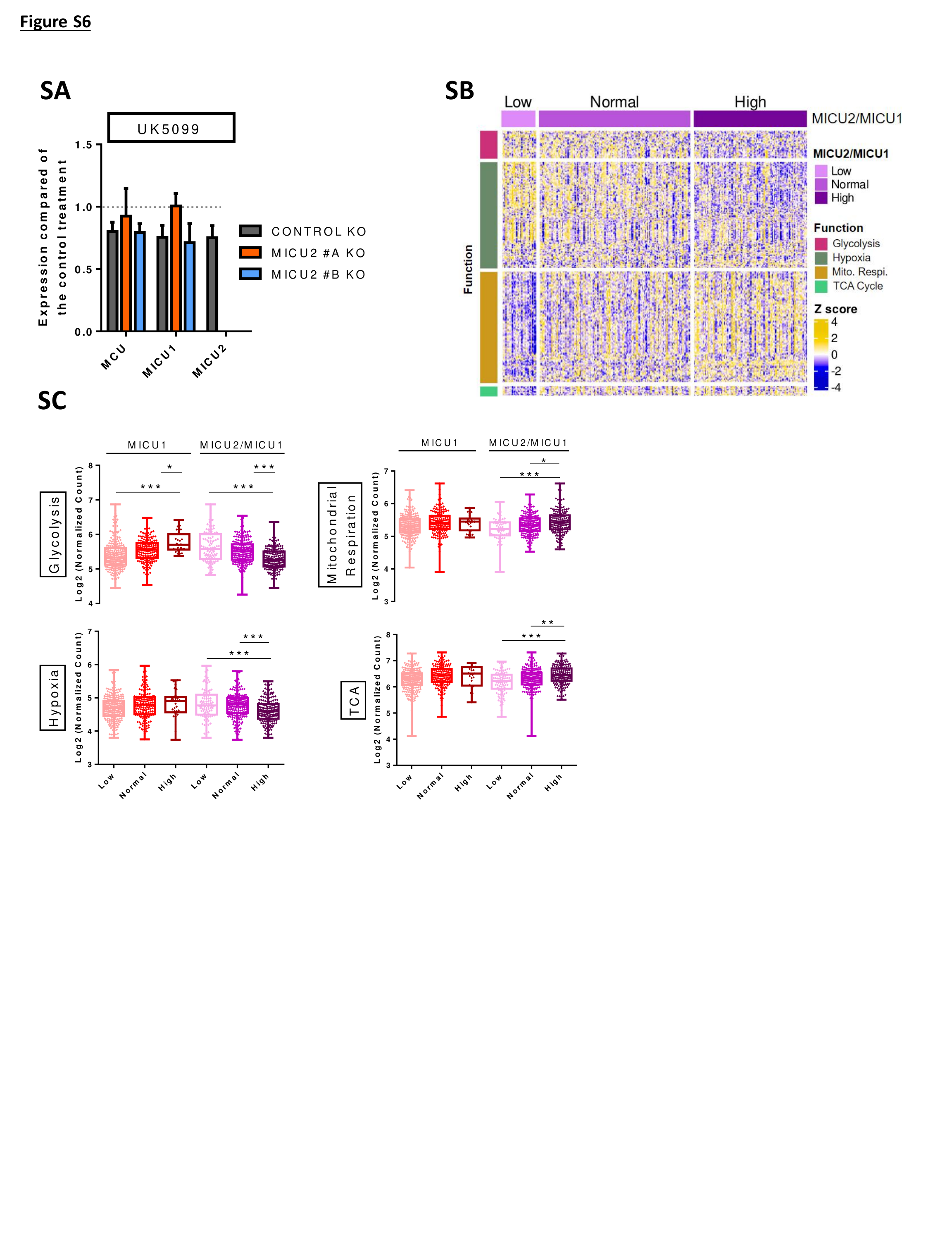

Supplement: S6 Fig — (A) The expression of MCU, MICU1, and MICU2 was determined in the Control and MICU2 KO cell lines treated with the inhibitor UK5099 using RT-qPCR. The data are presented as the mean ± standard error of the mean of 4 independent experiments. (B) Heatmap representing the expression of genes associated with glycolysis, hypoxia, mitochondrial respiration, and the TCA cycle as a function of the MICU2/MICU1 status of primary tumor samples from the TCGA-COAD data set. (C) Boxplot representing the mean expression of genes associated with glycolysis, hypoxia, mitochondrial respiration, and the TCA cycle according to the MICU1 and MICU2/MICU1 ratio status of primary tumor samples of the TCGA-COAD data set. (D) Scatter plot representing the correlation of the average expression of genes associated with glycolysis, hypoxia, mitochondrial respiration, and the TCA cycles as a function of the expression of MICU1, MICU2, or the MICU2/MICU1 ratio in a panel of 57 CRC cell lines from the RNA-Seq data set of the CCLE (R is the Pearson correlation coefficient). (E) Boxplots representing the average expression of genes associated with glycolysis, hypoxia, mitochondrial respiration, and the TCA cycle in a panel of 57 CRC cell lines from the RNA-Seq data set of the CCLE classified as low or high expression of MICU1, MICU2, or the MICU2/MICU1 ratio as a function of the median expression of MICU1, MICU2, or the MICU2/MICU1 ratio. On all plots, *p < 0.05, **p < 0.01, and ***p < 0.001. The data underlying the graphs shown in the figure can be found in S1 and S2 Datas. (TIF) [file pbio.3002854.s006.tif]

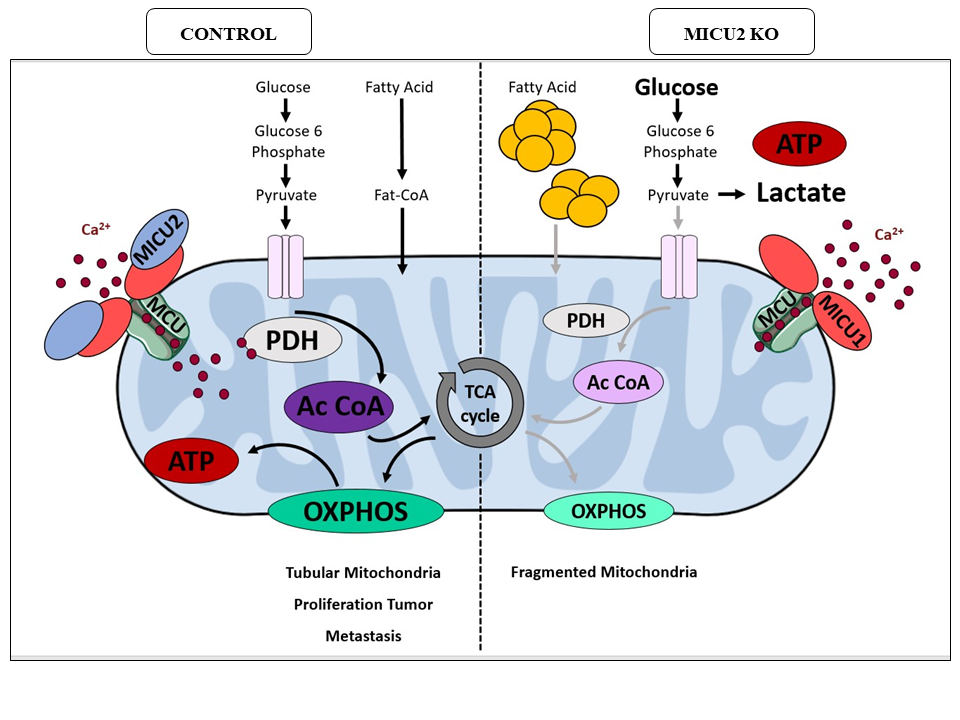

Supplement: S7 Fig — Graphical summary of the main study results. (TIF) [file pbio.3002854.s007.tif]
