# Supplementary material for: MICU2 up-regulation enhances tumor aggressiveness and metabolic reprogramming during colorectal cancer development
Source: PLoS Biol. 2024 Oct 28;22(10):e3002854. doi: 10.1371/journal.pbio.3002854 (PMC11542858; doi:10.1371/journal.pbio.3002854)
Supplement: S1 Table — (DOCX) [file pbio.3002854.s012.docx]

| Primers | Forward | Reverse |
| --- | --- | --- |
| MFN2 | TGAGAGGCATCAGTGAGGTG | GCAGAACTTTGTCCCAGAGC |
| OPA1 | AATCGGACCCAAGAACAGTG | ACTCCTCGGGATTCAAGGTT |
| FIS1 | GGAGGACCTGCTGAAGTTTG | ACGATGCCTTTACGGATGTC |
| DRP1 | GGAACAGCGAGATTGTGAGG | CCTTTGGCACACTGTCTTGA |
| PFKFB1 | ACATGGAAGCCCTGCAAAT | GGCTGAGATAGTTGTGAACATCC |
| PFKFB2 | ATCTCTCGGGGTGCCCTAT | TGCATAGGTCATCTCTTCACACA |
| PFKFB3 | CAACAGCTTTGAGGAGCATGT | GGGAGCCTTTCATGTTTTGT |
| PFKFB4 | CCAGATGAAGAGGACAATCCA | TCCTCGTAGGTCATTTCCTCA |
| PDK1 | CACCAAGACCTCGTGTTGAG | ACGTGATATGGGCAATCCAT |
| PDK2 | CTGGCCAACATCATGAAAGA | CCAGGAGGCTCTGGACATAC |
| PDK3 | TGTGTGAACAGTATTACCTGGTAGC | GTTTGTCTGGCGCTTTGG |
| PDK4 | CAGTGCAATTGGTTAAAAGCTG | GGTCATCTGGGCTTTTCTCA |
| PDP1 var 1 | ATGTTGTCGGCTCCGTGT | TGGAACTTCTGACTGGGATTC |
| PDP1 var 5 | TCGGGAAGAATCGTTTGGT | AAAAACAGTTGAGTTGGTGCTG |
| PDP2 | CTGAGCCTGAGGTCACATACC | AGGCCAGCACAAGGAACTTA |
| PDPR | GTGGCCTATCACCTCTCCAA | GCCAGCACAGAACCTGGTAG |
| GCKR | AGATGATATTCGGGCTGCTC | GGCGATGGGTATCACCTGT |
| DLAT | GGGTGAGAAGCTAAGTGAAGGA | CAGGGACCAGGATTTTTGC |
| DLD | CCCAGAGGTAGAATTCCAGTCA | GAGCCAGCATTGGACCAG |
| PDHA1 | GTCCGAGAGGCAACAAGGT | AAGTCTGCAGCTCCATCAGG |
| PDHB | CGGATAGAGGACACGACCA | GTCCAGTGAAAGCGCCTCT |
| DERA | CTGCGGGCCATTAGAGATT | AGAGAGCCAAGCAAGGGAAT |
| G6PD | GCAAACAGAGTGAGCCCTTC | GGCCAGCCACATAGGAGTT |
| H6PD | GGGTGGAGATCATCATGAAAG | GCGAATGACACCGTACTCCT |
| PGD | ATTGCTGCAAAAGTGGGAAC | GTTGTGCACCATCTTCACGA |
| PGLS | GCTGAGGACTACGCCAAGAA | AGGATCAGCAGGTCGAAAAC |
| PGM1 | GATGGACGCGAGCAAACT | GACAGCCCACAGTCCATCTT |
| PRPS1 | GCATTGCTCCAAAATACAGGT | AAGGGACATGGCTGAATAGG |
| PRPS2 | ACACTTGCGGCACCATCT | TCCCATGGGTAAGGATAGCA |
| RBKS | CTACAGGAACTGCTTCTATAATTGTCA | TCAGATCCTCCGTATTCAAAAGT |
| RPE | CGATGGTGGAGTAGGTCCTG | AGCACTGCCAGACACAATCA |
| RPIA | TCATCGTGATCGCTGATTTC | GATGACCTCGATGGGGATT |
| TALDO1 | CAGATGCCCGCTTACCAG | TTTAATCTGGTCCTCTTGTGACC |
| TKT | GGATGACCAGGTGACCGTTA | CGCGGATGTTGATCTTTTCT |
| UCP2 Blast 1 | AAGGGGATCGGGCCATGATA | GAGCTGGGTTGCAGGATGTT |
| UCP2 Blast 2 | CAAAGCCGGCTGGGTCTTAT | ACTGAGAAGGCTCAGGCAAA |
| UCP3 long | AGCCCCCTCGACTGTATGAT | ACTTTCATCAGGGCCCGTTT |
| UCP3 Short | AGCCCCCTCGACTGTATGAT | AGGAGGCTCACCCCTTGTAG |
| PRMT1 Cell | CCAGTGGAGAAGGTGGACAT | GTCATTCCGCTTCACTTGCA |
| PRMT1 Blast | CGCGAACTGCATCATGGAGAA | CTGGCCACAGGACACTTCTT |
| PLIN 2 | CCTCCTGTCCAACATCCAAG | GCATTGCGGAACACTGAGTA |
| PLIN 3 | CCACCAATGTGAAGGACCA | GAAGGAGTGGATGCTGGAAA |
| PLIN 4 | AATGAGCAACTTCGGAGCAC | CACCGTGTGACTTTGGACAG |
| PLIN 5 | CCACAAGCTGGGTTCTTCAC | TGATCCACCTGCCTCAGACT |
| HPRT1 | TGACCTTGATTTATTTTGCATACC | CGAGCAAGACGTTCAGTCCT |
| MCU | CGCCAGGAATATGTTTATCCA | CTTGTAATGGGTCTCTCAGTCTGTT |
| MICU1 | GAGGCAGCTCAAGAAGCACT | CAAACACCACATCACACACG |
| MICU1 Variant | CAATATATAATAAAACGCTTTGATGGAAAGA | GCGAATGATGCTCTGAACCT |
| MICU2 | GGCAGTTTTACAGTCTCCGC | AAGAGGAAGTCTCGTGGTGTC |
| MCT1 | GTGACCATTGTGGAATGCTG | CATGTCATTGAGCCGACCTA |
| MCT2 | GTTGACAGCGAGGCGAAT | CTTGTTTCAGGTTTCACAGGAA |
| MCT4 Var1,2&5 | GGGAAGGTCCAACCTTACACT | CCATCACAAACACATTCACCA |
| MCT4 Var3,6 | GGGAAGGTCCAACCTTACACT | AATAGCAACCAGGGGACCA |
| SLC2A1 | GGTTGTGCCATACTCATGACC | CAGATAGGACATCCAGGGTAGC |
| SLC2A3 | GCCCTGAAAGTCCCAGATTT | TTCATCTCCTGGATGTCTTGG |
| SLC2A4 | CTGTGCCACCTGATGACTG | CGTAGCTCATGGCTGGAACT |
| HK1 | CACCTGTGAGGTTGGACTCA | CCACCATCTCCACGTTCTTC |
| HK2 | TCCCCTGCCACCAGACTA | TGGACTTGAATCCCTTGGTC |
| GPI | CCCTATGACCAGTACCTGCAC | TTCCCATTGGACTCCATGTC |
| PFKM | GCCATCAGCCTTTGACAGA | CTCCAAAAGTGCCATCACTG |
| PFKL | GCTTCGACACCCGTGTAACT | ATGCCCATCTTGCTGCTC |
| ALDOA | TGCCAGTATGTGACCGAGAA | GCCTTCCAGGTAGATGTGGT |
| ALDOC | GAACCCGAGCTGTGCTTG | GTACGAGTGAGGCATGGTGA |
| TPI1 | GTTGGGGAAACTGGAAGAT | TAGGGGGAGCACAAACCAC |
| PGK1 | CAGCTGCTGGGTCTGTCAT | GCTGGCTCGGCTTTAACC |
| PGK2 | CCAGCTCCTGGTTCAGTCA | TCCCTTCTTCCTCCACATGA |
| PGAM1 | GGAGGGGAAACGTGTACTGAT | AGCTCCATGATAGCCTCTTCA |
| ENO1 | TCCCAACATCCTGGAGAATAA | ATGCCGATGACCACCTTATC |
| PKM1 | CAGCCAAAGGGGACTATCCT | CCTCAGCCTCACGAGCTATC |
| PKM2 | CAGCCAAAGGGGACTATCCT | CAAATAATTGCAAGTGGTAGATGG |
| LDHA | GTCCTTGGGGAACATGGAG | TTCAGAGAGACACCAGCAACA |
| LDHB | GATGGATTTTGGGGGAACAT | AACACCTGCCACATTCACAC |
| LDHC | GCTCTGAAGACTCTGGACCCTA | TCAGCTTGATAATTTCATAGGCACT |
| CPT1 | CCTCCGTAGCTGACTCGGTA | GGAGTGACCGTGAACTGAAAG |
| CPT2 | TGACCAAAGAAGCAGCAATG | GAGCTCAGGAAGATGATCC |

Supplementary table 1. List of primers for real-time polymerase chain reaction
