# Supplementary material for: MICU2 up-regulation enhances tumor aggressiveness and metabolic reprogramming during colorectal cancer development
Source: PLoS Biol. 2024 Oct 28;22(10):e3002854. doi: 10.1371/journal.pbio.3002854 (PMC11542858; doi:10.1371/journal.pbio.3002854)
Supplement: S2 Table — (DOCX) [file pbio.3002854.s013.docx]

| REAGENT or RESOURCE | SOURCE | IDENTIFIER |
| --- | --- | --- |
| Antibodies |  |  |
| MICU2 | Abcam | ab101465 |
| MICU1 | Sigma | HPA037479 |
| MCU | Cell signaling | #14997 |
| OPA1 | BD Biosciences | 612607 |
| MFN2 | Cell signaling | #11925 |
| DRP1 (D8H5) | Cell signaling | #5391 |
| P-DRP1 (S616) | Cell signaling | #3455 |
| NDUFS2 | Abcam | ab192022 |
| SDHA | Abcam | ab137040 |
| UQCRC2 | Abcam | ab14745 |
| MT-CO1 | Abcam | ab14705 |
| Chemicals, peptides and recombinant proteins |  |  |
| CyQuant | Thermo Fisher Scientific | #35006 |
| Mitotracker | Invitrogen/Molecular Probes | M7514 |
| mt-riG6m | Li et al. 2020 (https://doi.org/10.1016/j.ceca.2020.102165) |  |
| miGer | Li et al. 2020 (https://doi.org/10.1016/j.ceca.2020.102165) |  |
| Nucleospin RNA plus Kit | Macherey–Nagel | 740984-250 |
| PrimeScript RT Reagent Kit | Takara | RR037A |
| SYBR Green Master kit | Takara | RR420L |
| Hypoxyprobe kit | Hypoxyprobe | HP1-1000Kit |
| Fura-2-AM | Thermo Fisher Scientific | F1221 |
| MitoSox Red | Thermo Fisher Scientific | M36008 |
| BCA protein assay kit | Thermo Fisher Scientific | 23227 |
| FAOBlue | Funakoshi (Japan) | FDV-0033 |
| Inhibitors |  |  |
| 2-Deoxy-D-glucose | Sigma-Aldrich | D8375 |
| 2NBDG | Abcam | ab146200 |
| UK5099 | MedChemExpress | HY-15475 |
| Rotenone | TOCRIS | CAS 83-79-4 |
| Oligomycin | Abcam | ab141829 |
| Trimetazidine | Sigma-Aldrich | 653322 |
| Etomoxir | Sigma-Aldrich | E1905 |
| Telaglenastat | MedChemExpress | HY-12248 |
| V-9302 | MedChemExpress | HY-112683 |
| BAPTA | Sigma-Aldrich | A1076 |
| MITOTEMPO | Sigma-Aldrich | SML0737 |
| Treatments |  |  |
| 5-Fluorouracil | Fluorouracile Accord 50mg/ml |  |
| Oxaliplatin | Oxaliplatine Accord 5mg/ml |  |
| Software |  |  |
| Image Lab | Bio Rad |  |
| GraphPad Prism 6 |  |  |
| Kaluza 1.3 software | Beckman Coulter |  |
| T-scratch software |  |  |
| Image 4.4 software | Perkin Elmer |  |
| VisualSonics VevoLAZR System | FUJIFILM |  |
| ImageJ/Fiji |  |  |
| Metamorph 7.7 | Molecular Devices |  |
| Imaris 8.0 | Bitplane |  |
| SoftMax Pro 4.5.6 |  |  |
| R 4.3.0 | R-Cran |  |

Supplementary Table 2. List of key resources
