# Supplementary material for: MICU2 up-regulation enhances tumor aggressiveness and metabolic reprogramming during colorectal cancer development
Source: PLoS Biol. 2024 Oct 28;22(10):e3002854. doi: 10.1371/journal.pbio.3002854 (PMC11542858; doi:10.1371/journal.pbio.3002854)
Supplement: S1 Raw Images — (PDF) [file pbio.3002854.s014.pdf]

Colon lines
Figure 1C

MICU1 (≈50 / 55kDa)

01_04_23

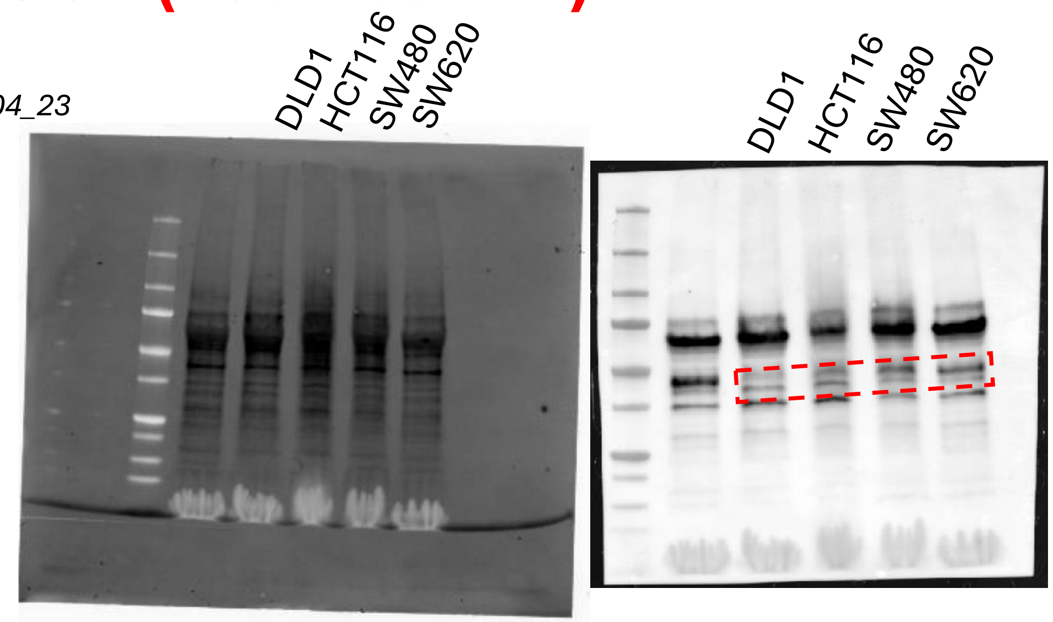

06_04_23

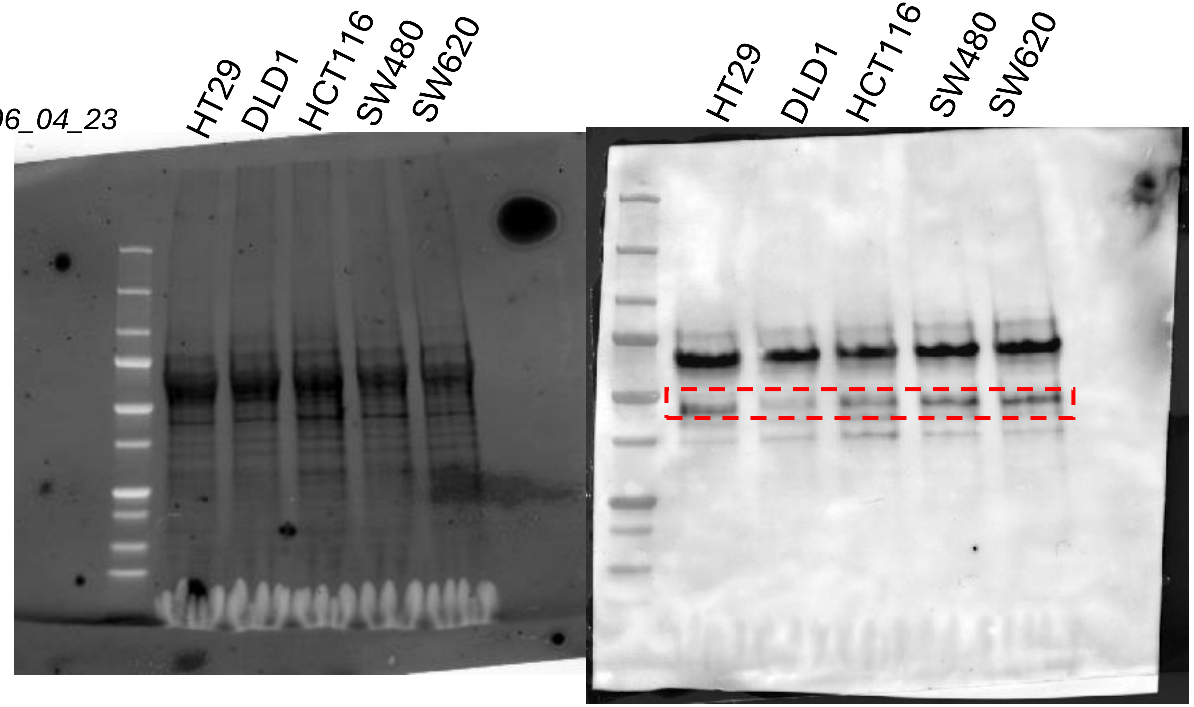

28_04_23

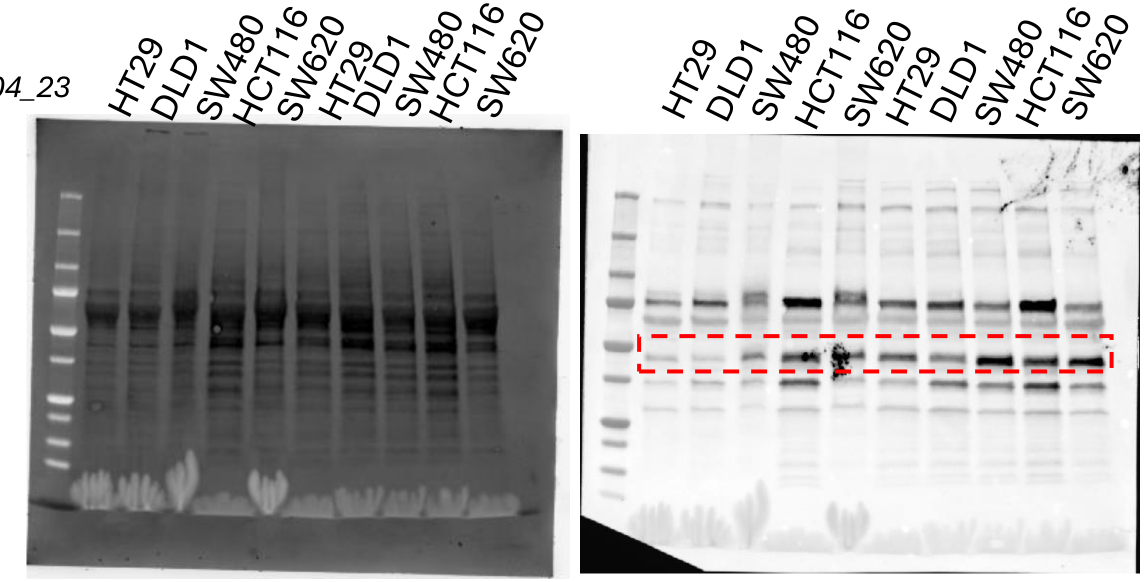

06_05_23

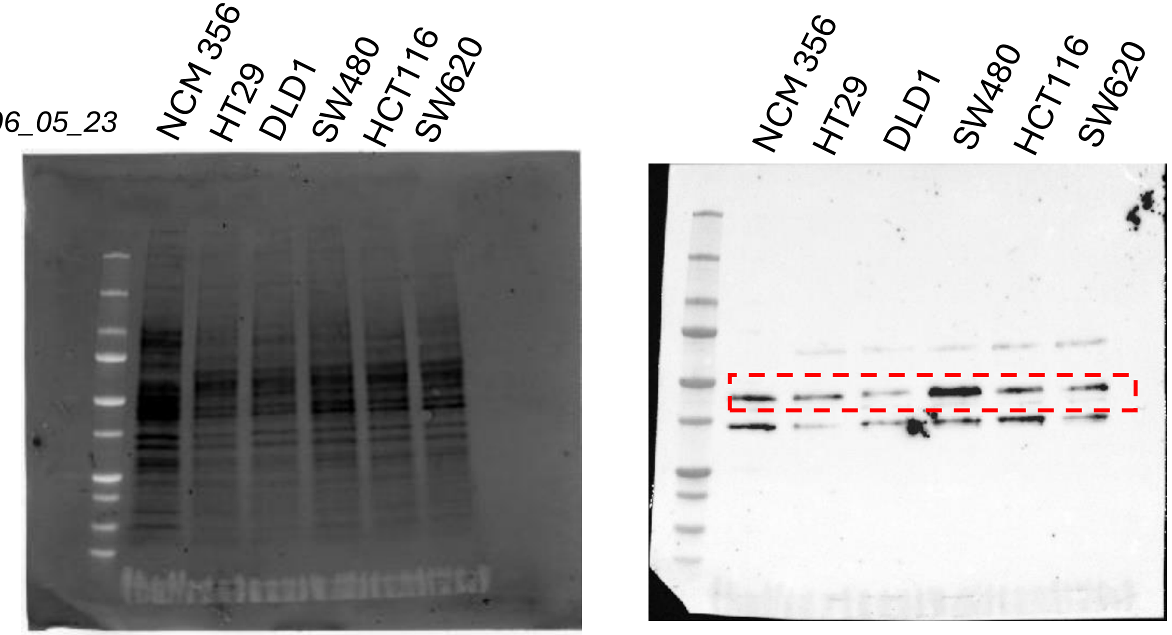

MICU1 (≈50 / 55kDa)

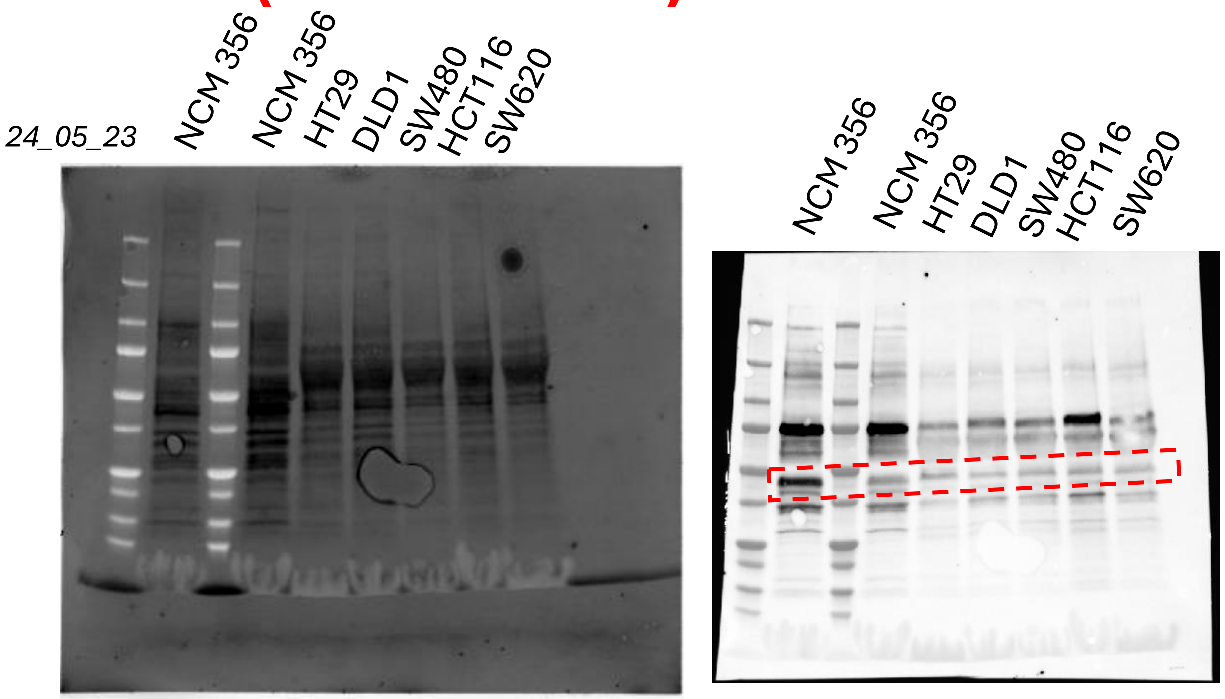

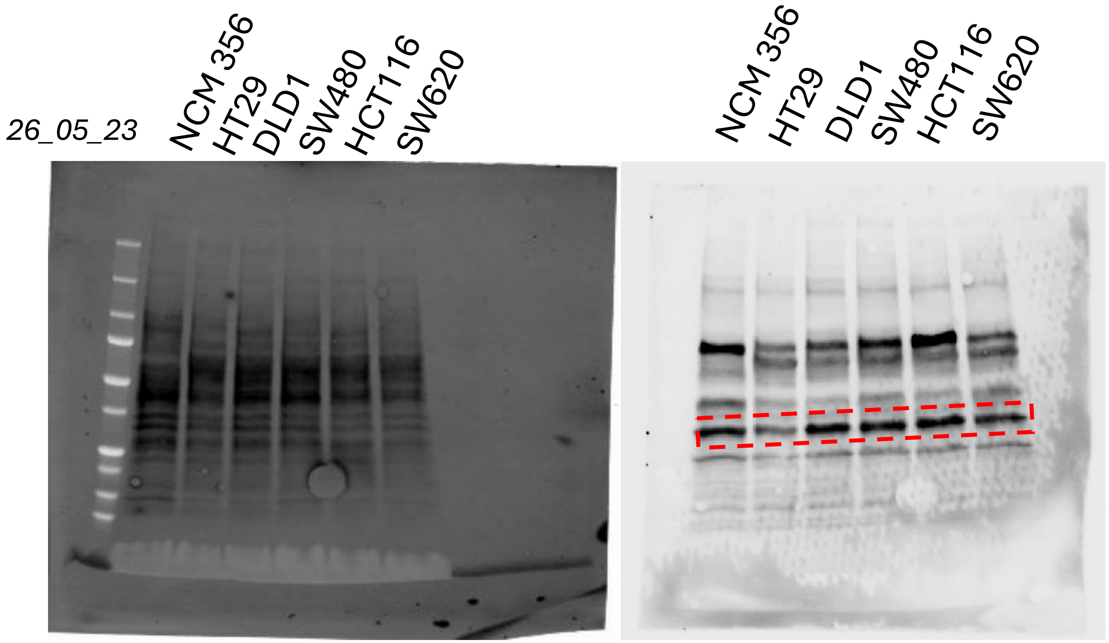

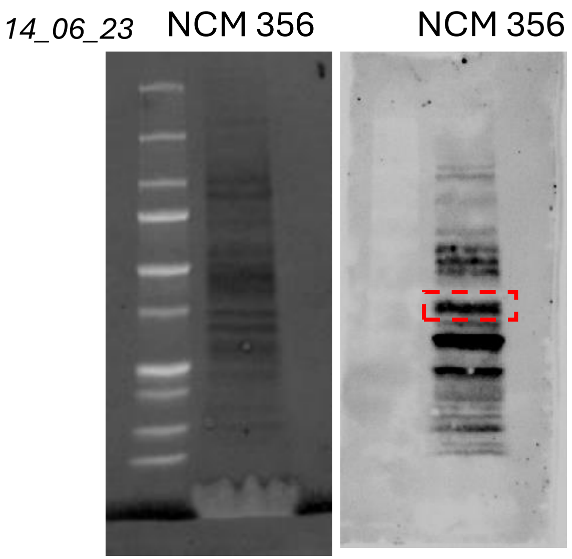

MICU2 (≈50kDa)

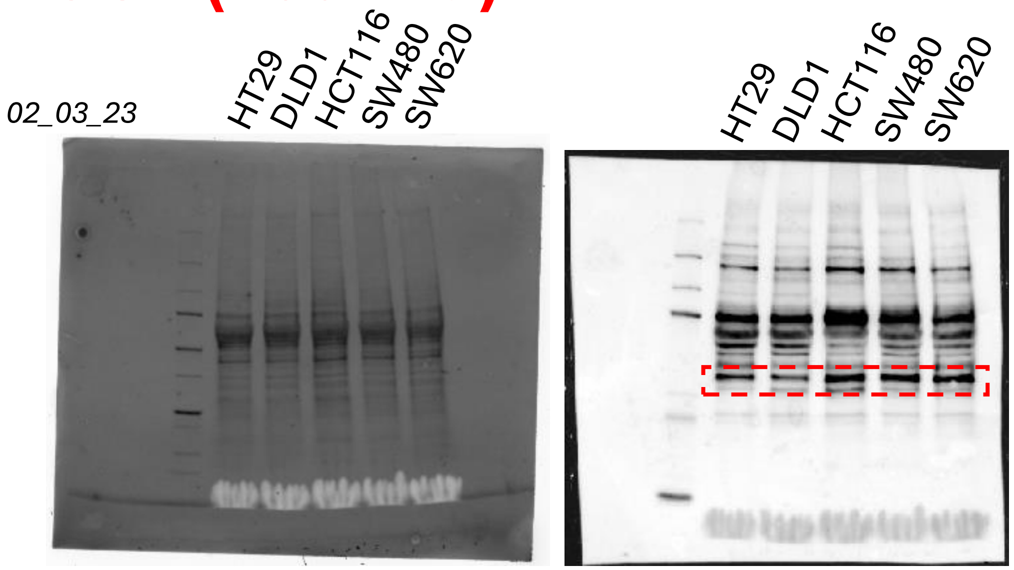
02_03_23
HT29
DLD1
HCT116
SW480
SW620
HT29
DLD1
HCT116
SW480
SW620

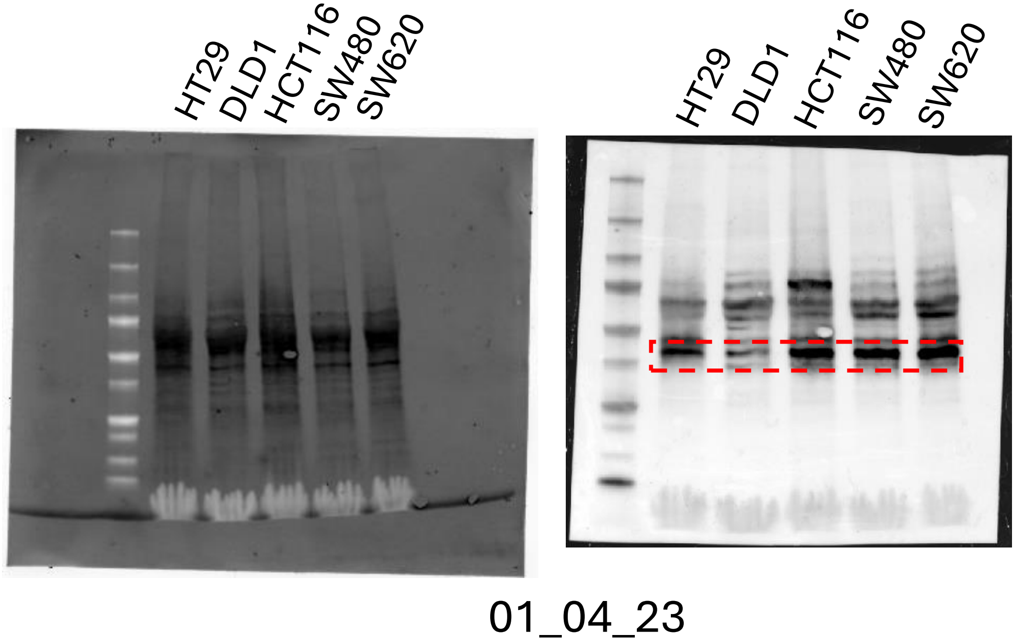
HT29
DLD1
HCT116
SW480
SW620
HT29
DLD1
HCT116
SW480
SW620
01_04_23

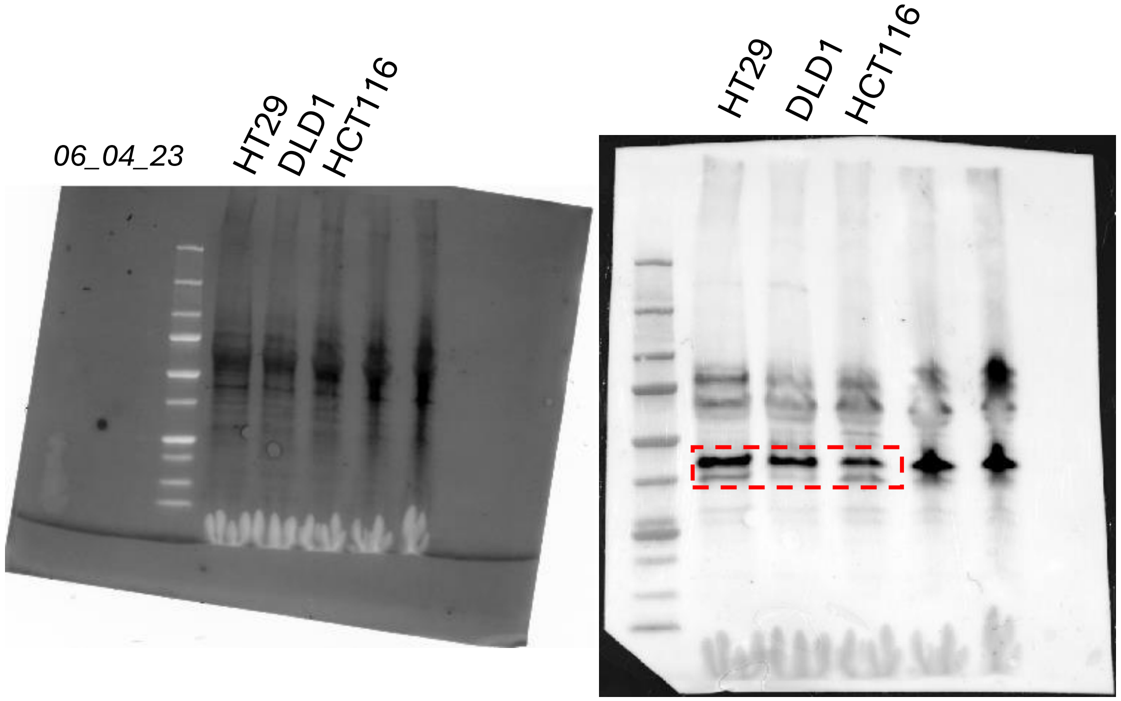
06_04_23
HT29
DLD1
HCT116
HT29
DLD1
HCT116

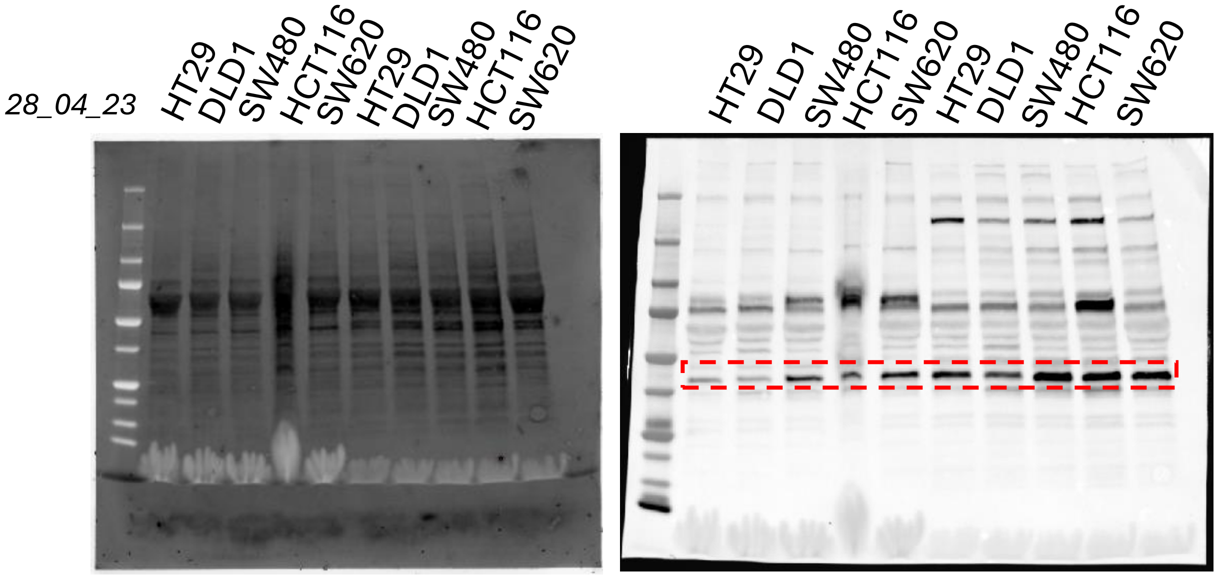
28_04_23
HT29
DLD1
SW480
HCT116
SW620
HT29
DLD1
SW480
HCT116
SW620
HT29
DLD1
SW480
HCT116
SW620
HT29
DLD1
SW480
HCT116
SW620

MICU2 (≈50kDa)

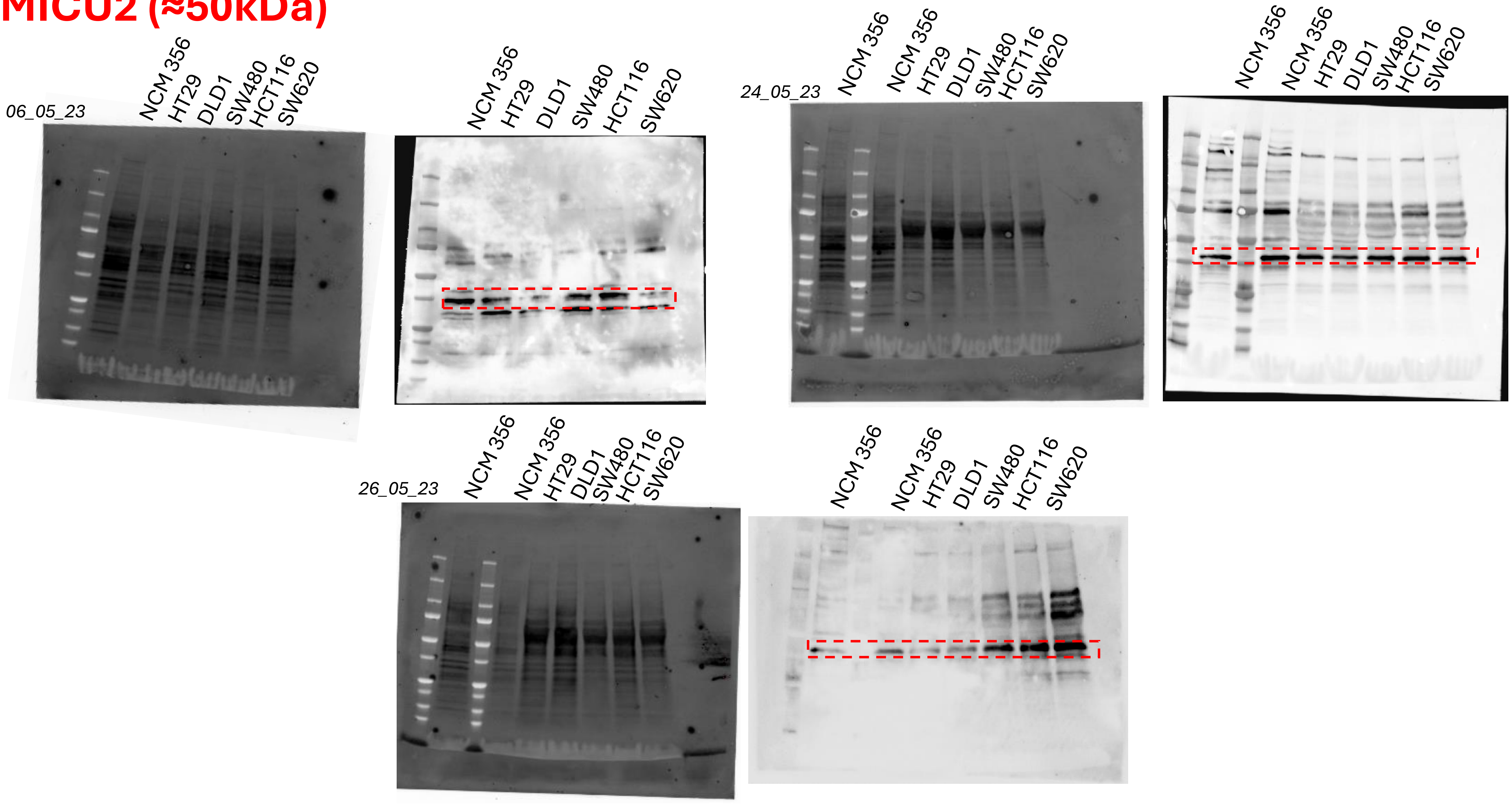
06_05_23
NCM 356
HT29
DLD1
SW480
HCT116
SW620
NCM 356
HT29
DLD1
SW480
HCT116
SW620
24_05_23
NCM 356
NCM 356
HT29
DLD1
SW480
HCT116
SW620
NCM 356
NCM 356
HT29
DLD1
SW480
HCT116
SW620
26_05_23
NCM 356
NCM 356
HT29
DLD1
SW480
HCT116
SW620
NCM 356
NCM 356
HT29
DLD1
SW480
HCT116
SW620

MICU2 (≈50kDa)

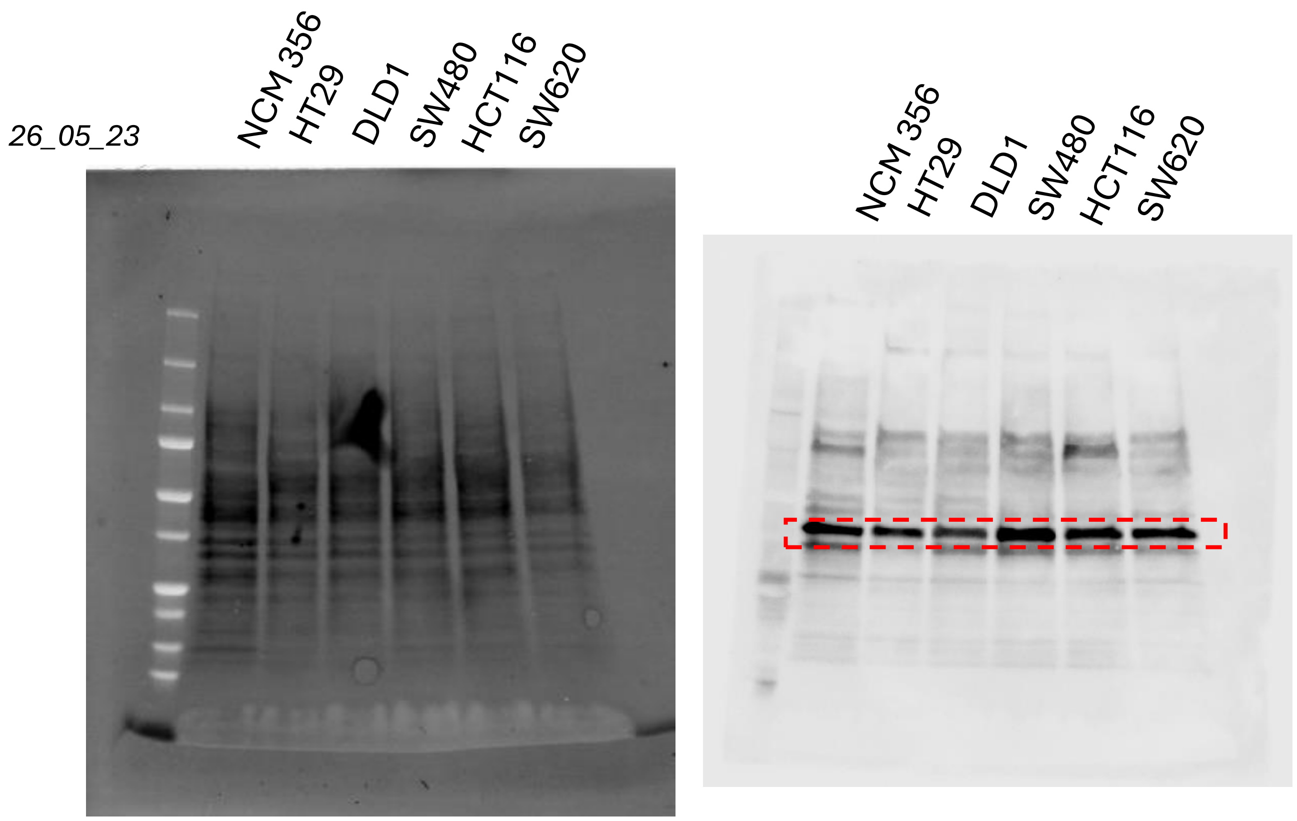
26_05_23
NCM 356
HT29
DLD1
SW480
HCT116
SW620
NCM 356
HT29
DLD1
SW480
HCT116
SW620

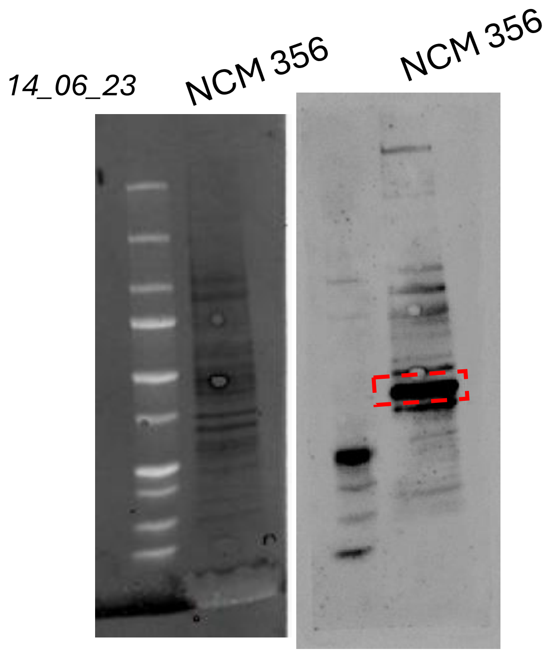
14_06_23
NCM 356
NCM 356

MCU (≈35 / 40kDa)

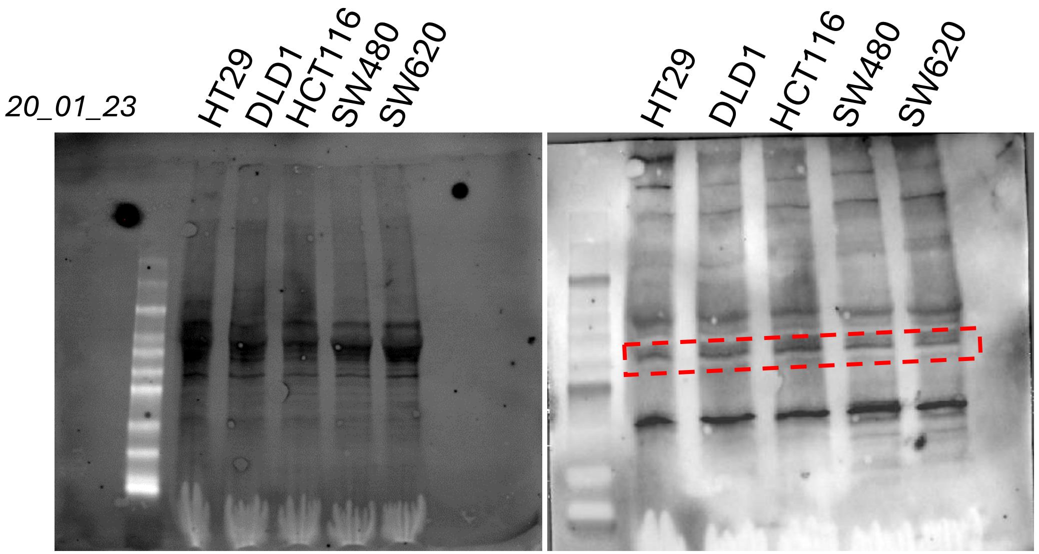
20_01_23
HT29
DLD1
HCT116
SW480
SW620
HT29
DLD1
HCT116
SW480
SW620

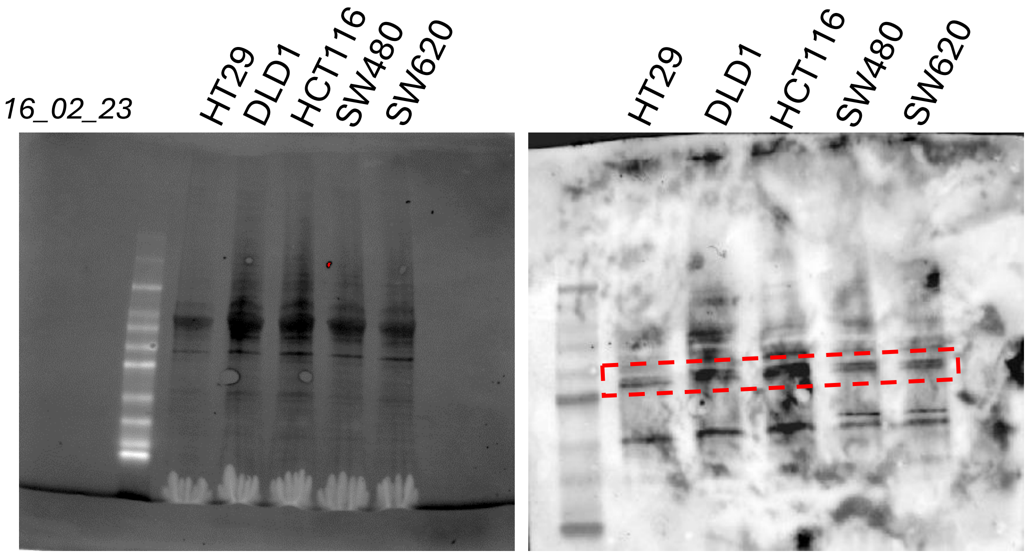
16_02_23
HT29
DLD1
HCT116
SW480
SW620
HT29
DLD1
HCT116
SW480
SW620

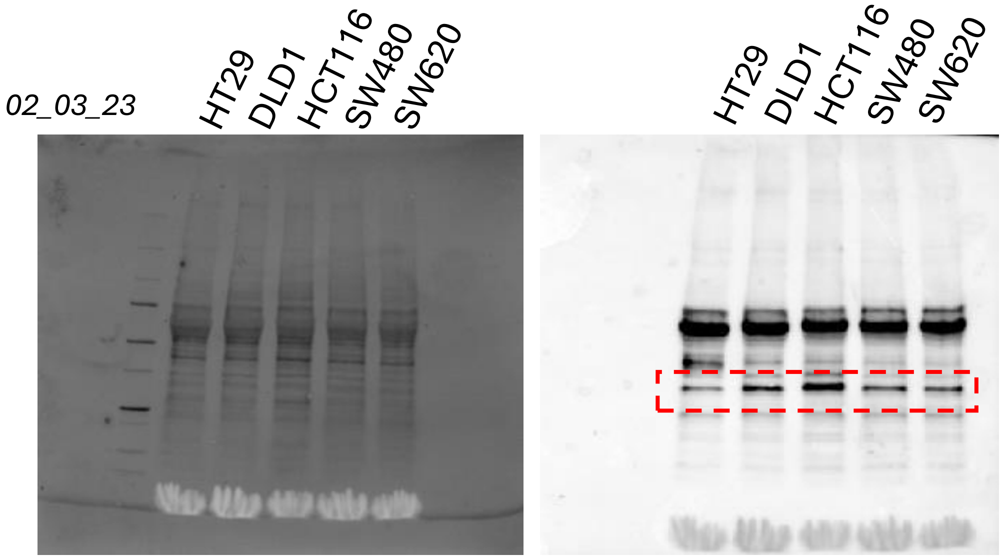
02_03_23
HT29
DLD1
HCT116
SW480
SW620
HT29
DLD1
HCT116
SW480
SW620

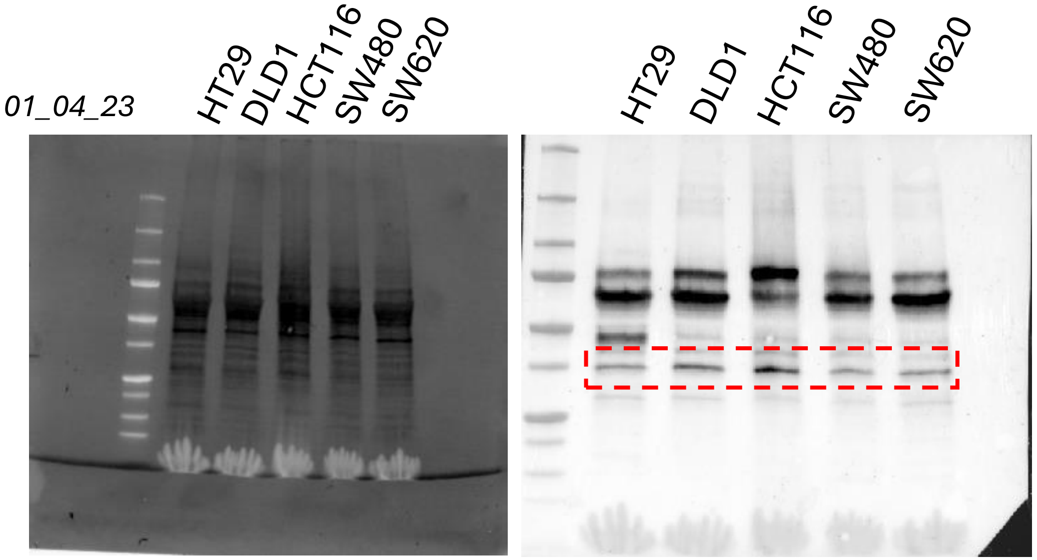
01_04_23
HT29
DLD1
HCT116
SW480
SW620
HT29
DLD1
HCT116
SW480
SW620

MCU (≈35 / 40kDa)

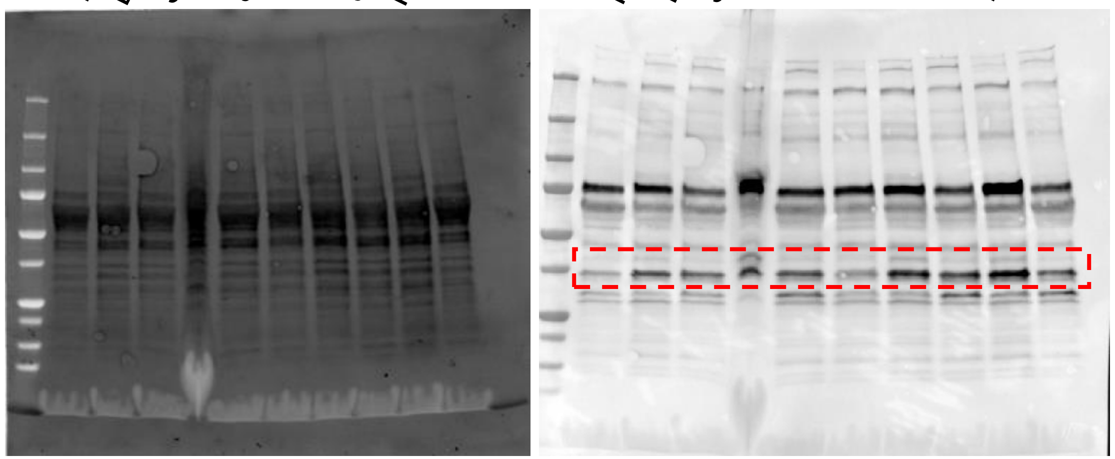
28_04_23
HT29
DLD1
SW480
SW620
HT29
DLD1
SW480
HCT116
SW620
HT29
DLD1
SW480
SW620
HT29
DLD1
SW480
HCT116
SW620

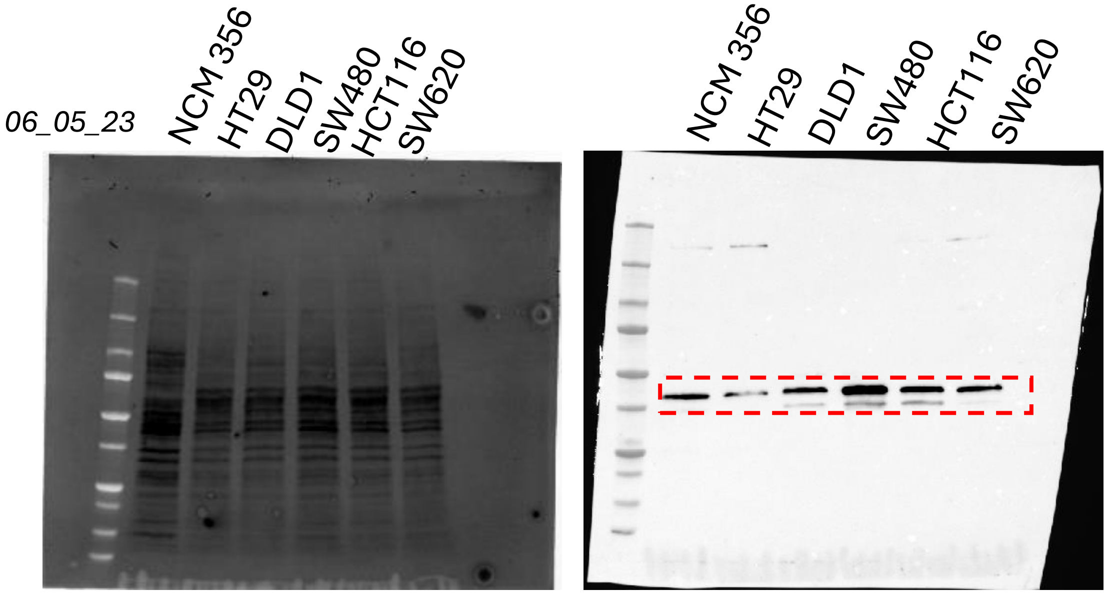
06_05_23
NCM 356
HT29
DLD1
SW480
HCT116
SW620
NCM 356
HT29
DLD1
SW480
HCT116
SW620

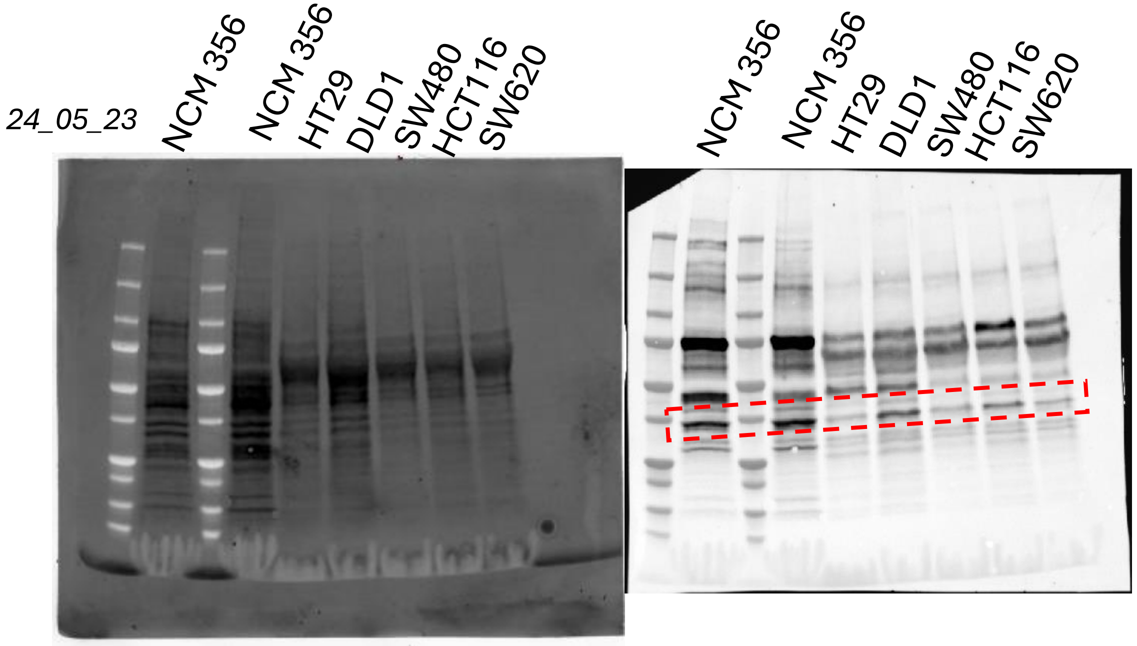
24_05_23
NCM 356
NCM 356
HT29
DLD1
SW480
HCT116
SW620
NCM 356
NCM 356
HT29
DLD1
SW480
HCT116
SW620

KO lines

Figure 1D

MICU1 (≈50 / 55kDa)

16_11_22

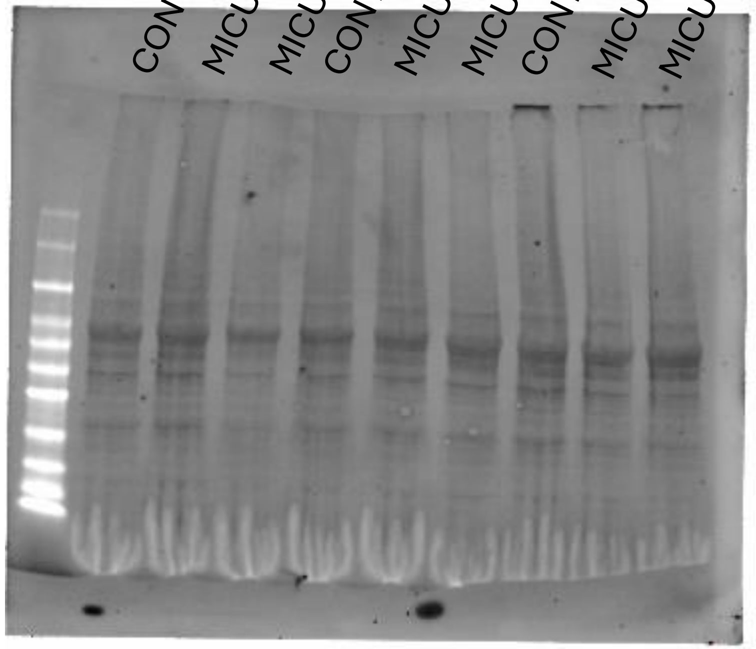

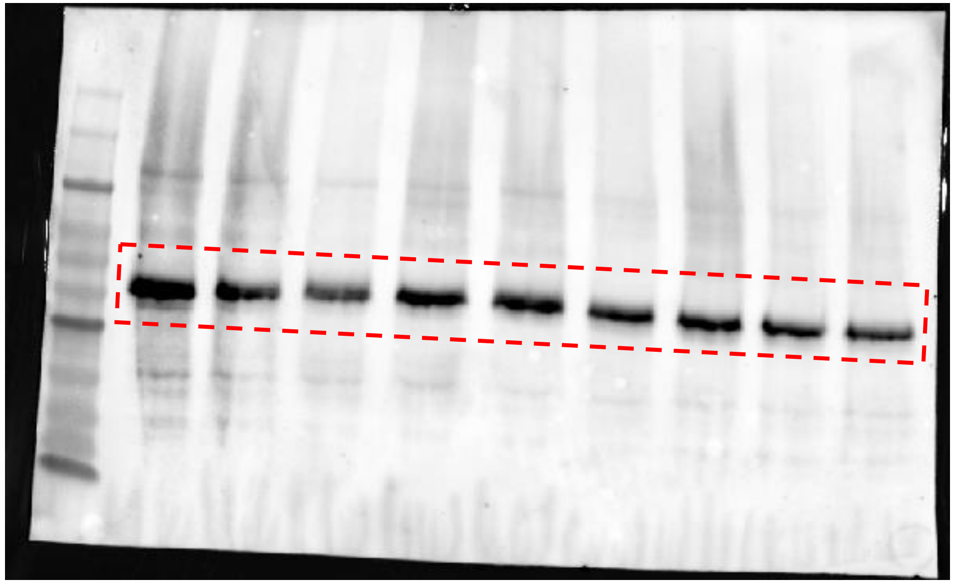

MICU1 (≈50 / 55kDa)

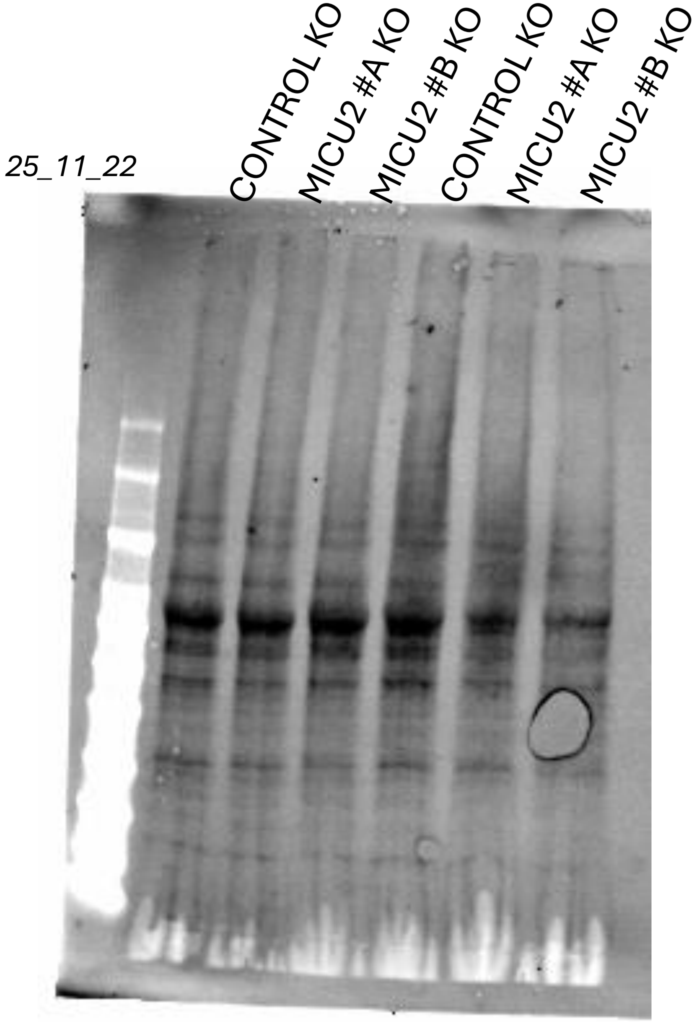

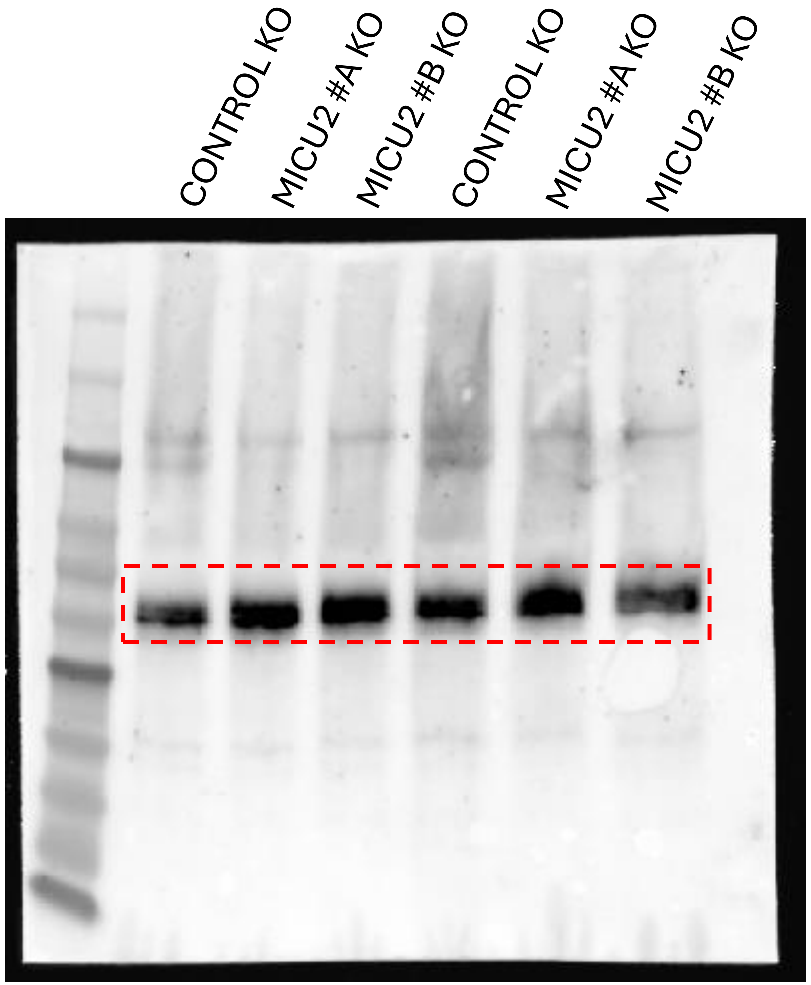

MICU2 (≈50kDa)

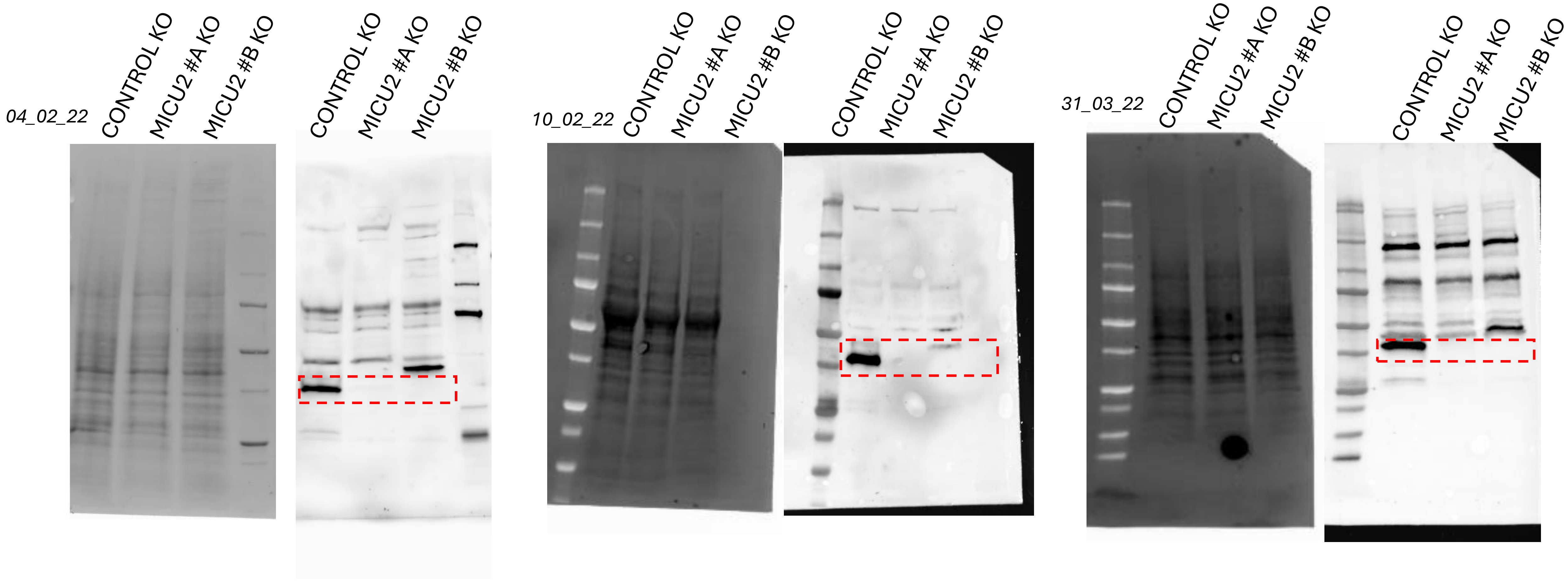
04_02_22
CONTROL KO
MICU2 #A KO
MICU2 #B KO
CONTROL KO
MICU2 #A KO
MICU2 #B KO
10_02_22
CONTROL KO
MICU2 #A KO
MICU2 #B KO
CONTROL KO
MICU2 #A KO
MICU2 #B KO
31_03_22
CONTROL KO
MICU2 #A KO
MICU2 #B KO
CONTROL KO
MICU2 #A KO
MICU2 #B KO

MICU2 (≈50kDa)

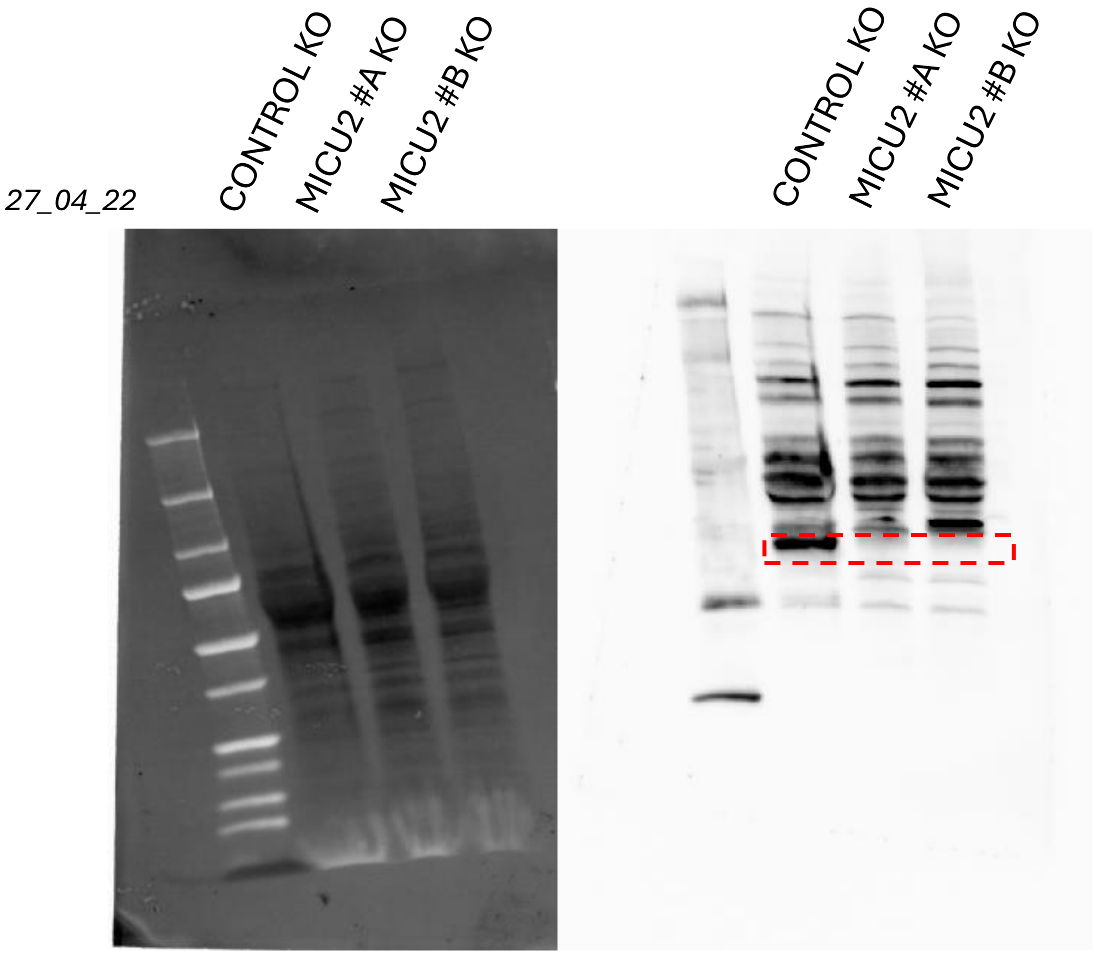
27_04_22
CONTROL KO
MICU2 #A KO
MICU2 #B KO
CONTROL KO
MICU2 #A KO
MICU2 #B KO

MICU2 (≈50kDa)

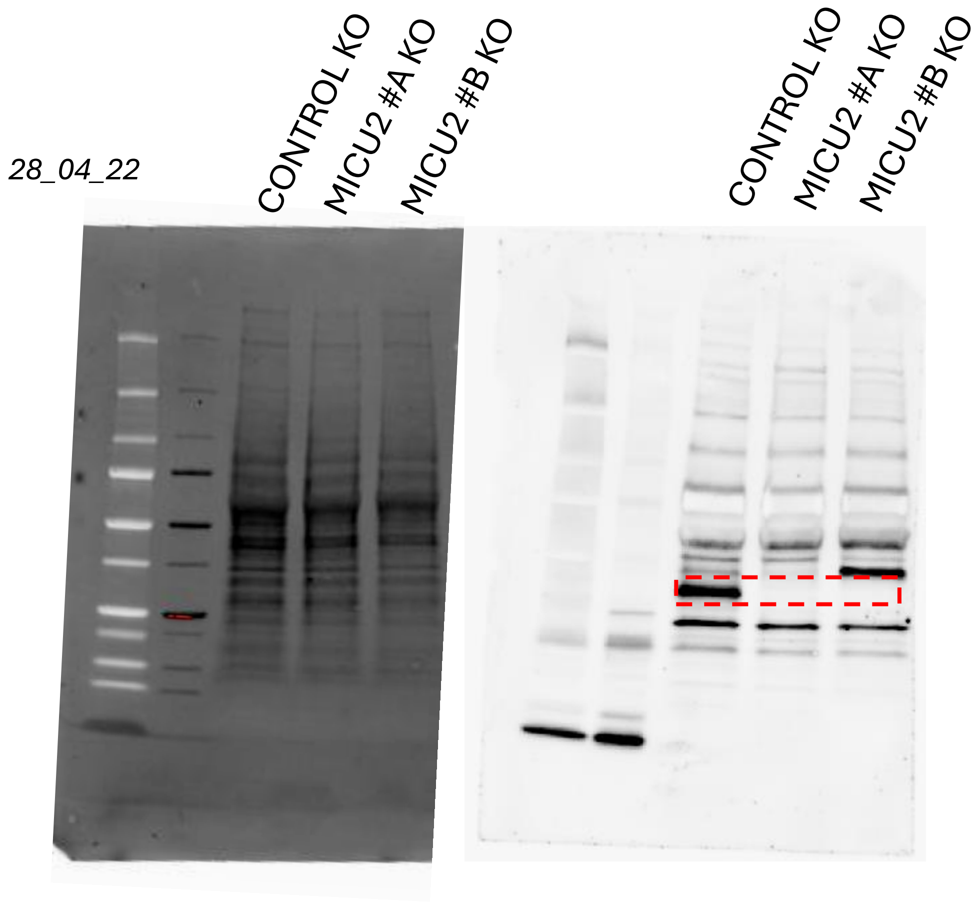
28_04_22
CONTROL KO
MICU2 #A KO
MICU2 #B KO
CONTROL KO
MICU2 #A KO
MICU2 #B KO

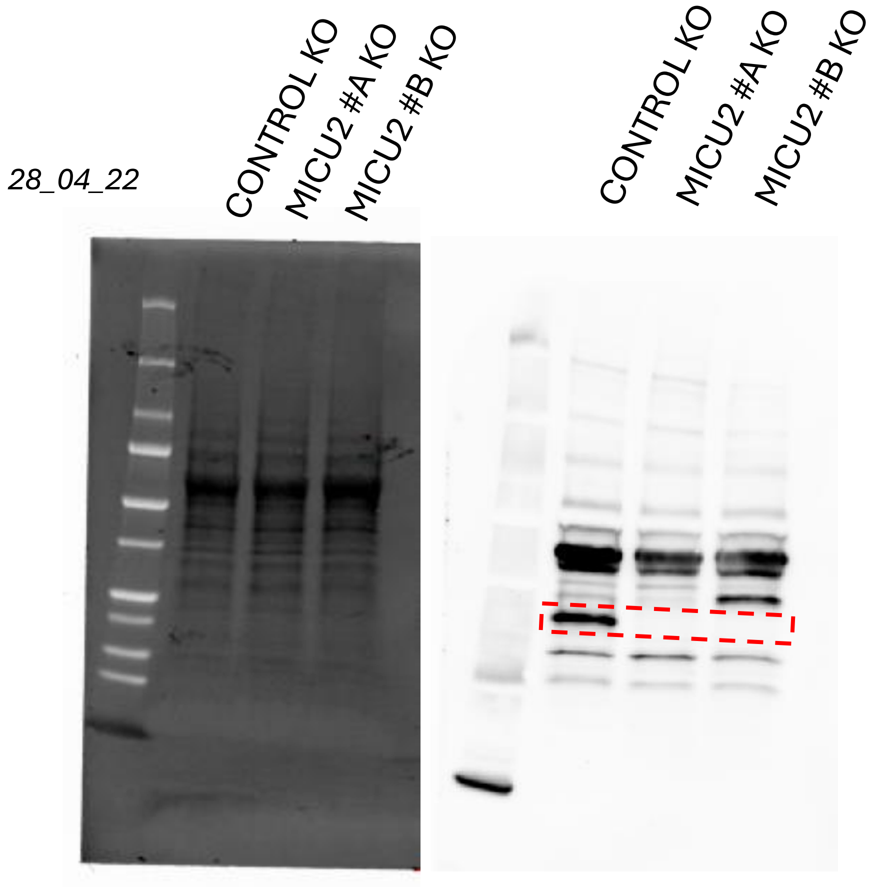
28_04_22
CONTROL KO
MICU2 #A KO
MICU2 #B KO
CONTROL KO
MICU2 #A KO
MICU2 #B KO

MCU (≈35 / 40kDa)

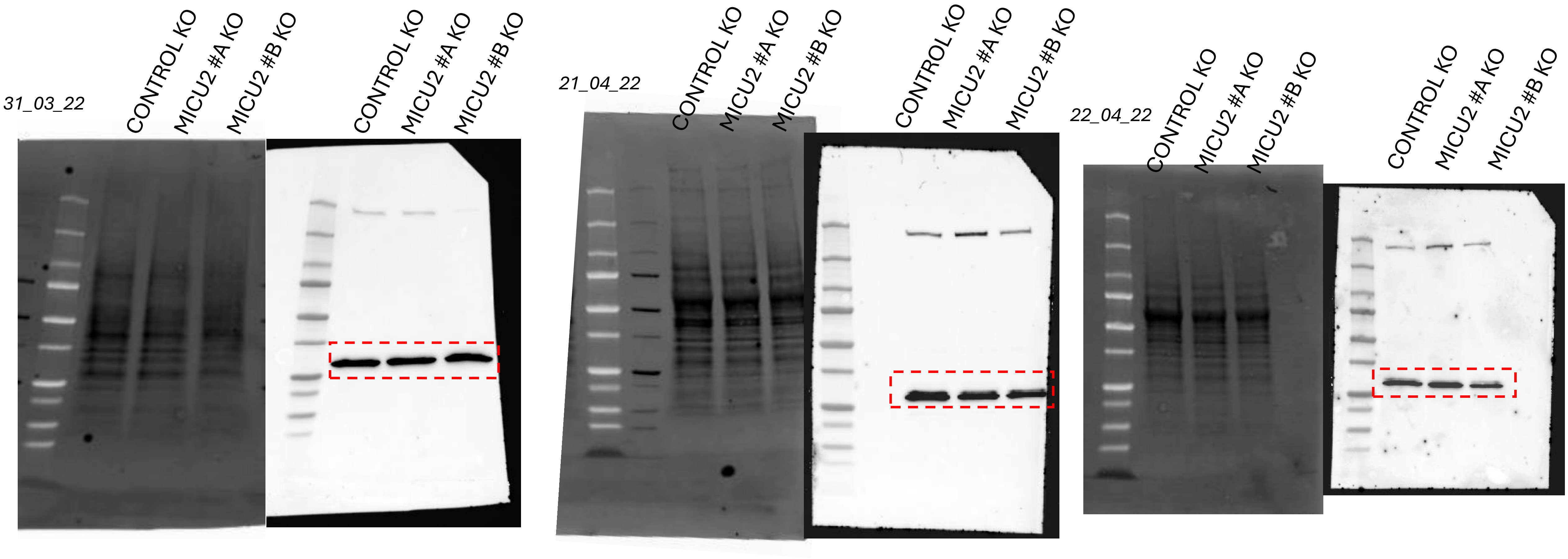

31_03_22
CONTROL KO
MICU2 #A KO
MICU2 #B KO
CONTROL KO
MICU2 #A KO
MICU2 #B KO
21_04_22
CONTROL KO
MICU2 #A KO
MICU2 #B KO
CONTROL KO
MICU2 #A KO
MICU2 #B KO
22_04_22
CONTROL KO
MICU2 #A KO
MICU2 #B KO
CONTROL KO
MICU2 #A KO
MICU2 #B KO

MCU (≈35 / 40kDa)

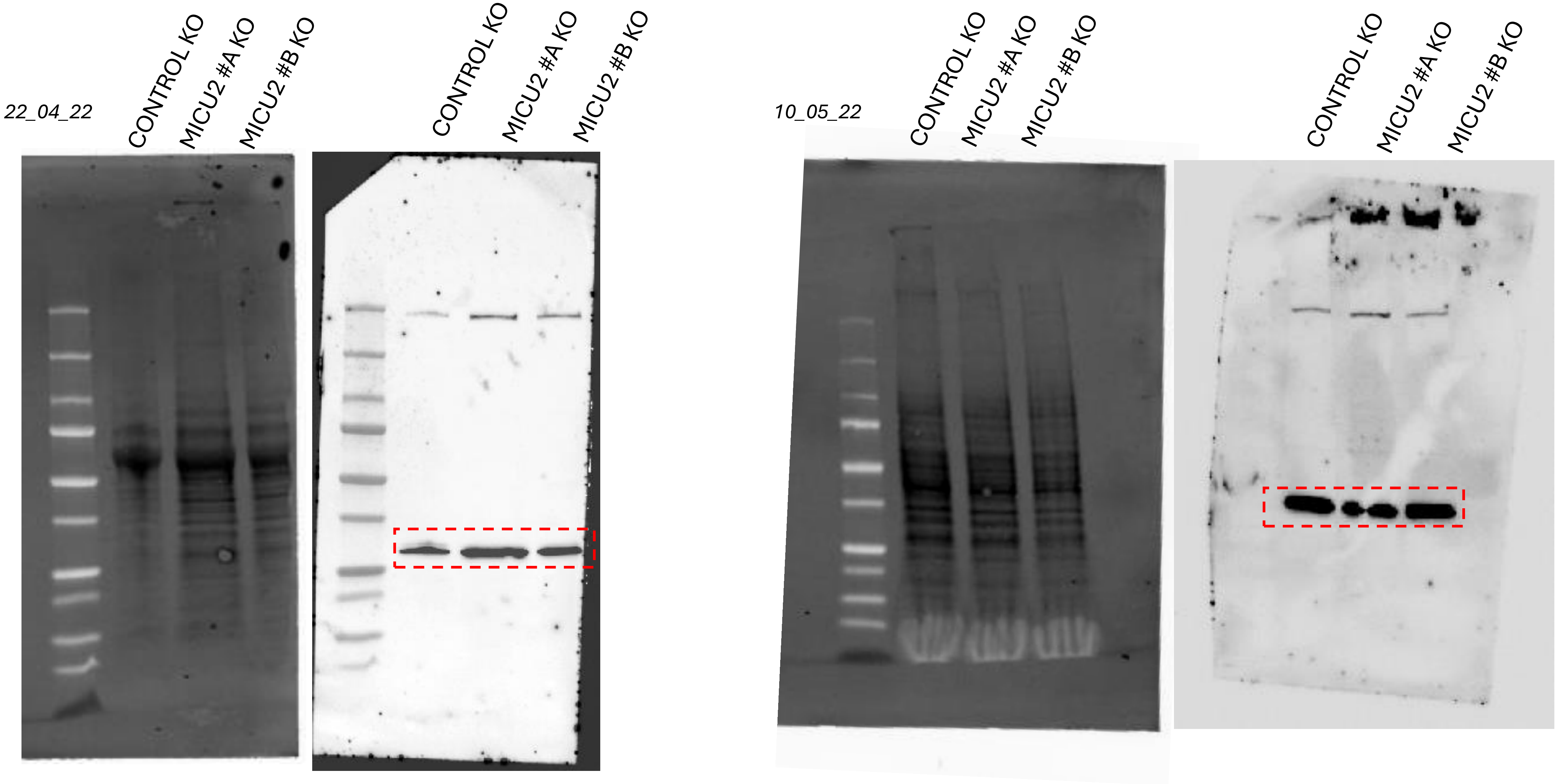
22_04_22
CONTROL KO
MICU2 #A KO
MICU2 #B KO
CONTROL KO
MICU2 #A KO
MICU2 #B KO
10_05_22
CONTROL KO
MICU2 #A KO
MICU2 #B KO
CONTROL KO
MICU2 #A KO
MICU2 #B KO

KO lines
Figure 1E

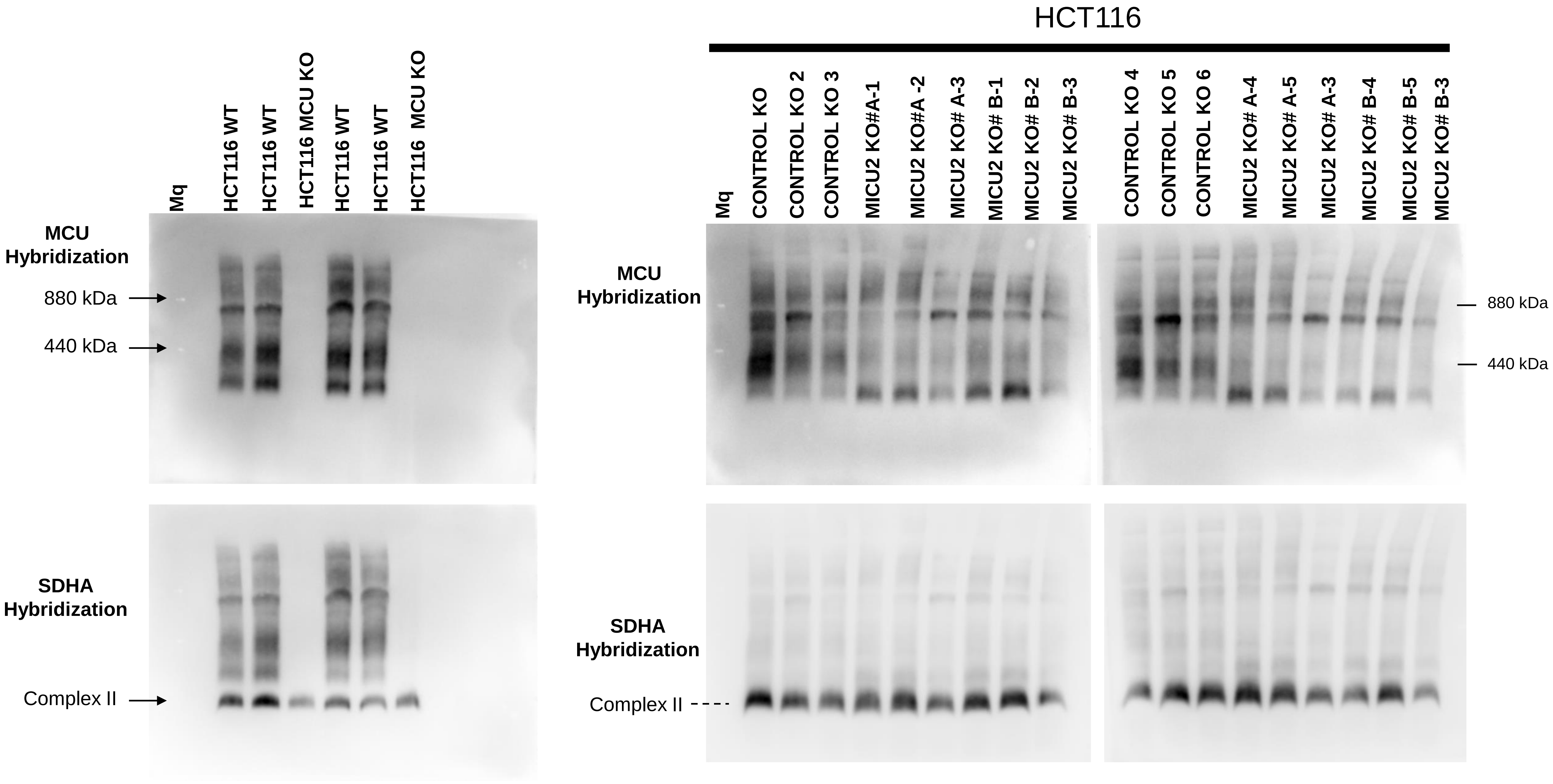

HCT116
Mq
HCT116 WT
HCT116 WT
HCT116 MCU KO
HCT116 WT
HCT116 WT
HCT116 MCU KO
MCU Hybridization
880 kDa
440 kDa
SDHA Hybridization
Complex II
Mq
CONTROL KO
CONTROL KO 2
CONTROL KO 3
MICU2 KO#A-1
MICU2 KO#A -2
MICU2 KO# A-3
MICU2 KO# B-1
MICU2 KO# B-2
MICU2 KO# B-3
CONTROL KO 4
CONTROL KO 5
CONTROL KO 6
MICU2 KO# A-4
MICU2 KO# A-5
MICU2 KO# A-3
MICU2 KO# B-4
MICU2 KO# B-5
MICU2 KO# B-3
MCU Hybridization
880 kDa
440 kDa
SDHA Hybridization
Complex II

KO lines
Figure 3D

OPA1 (≈80 / 100kDa)

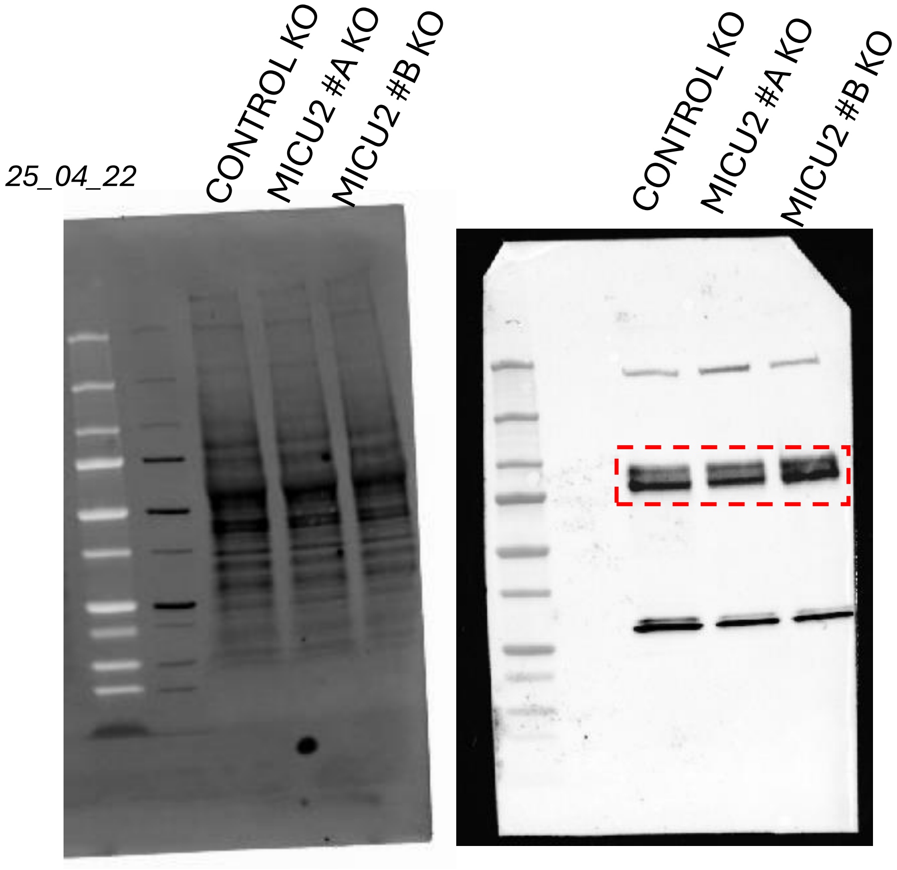

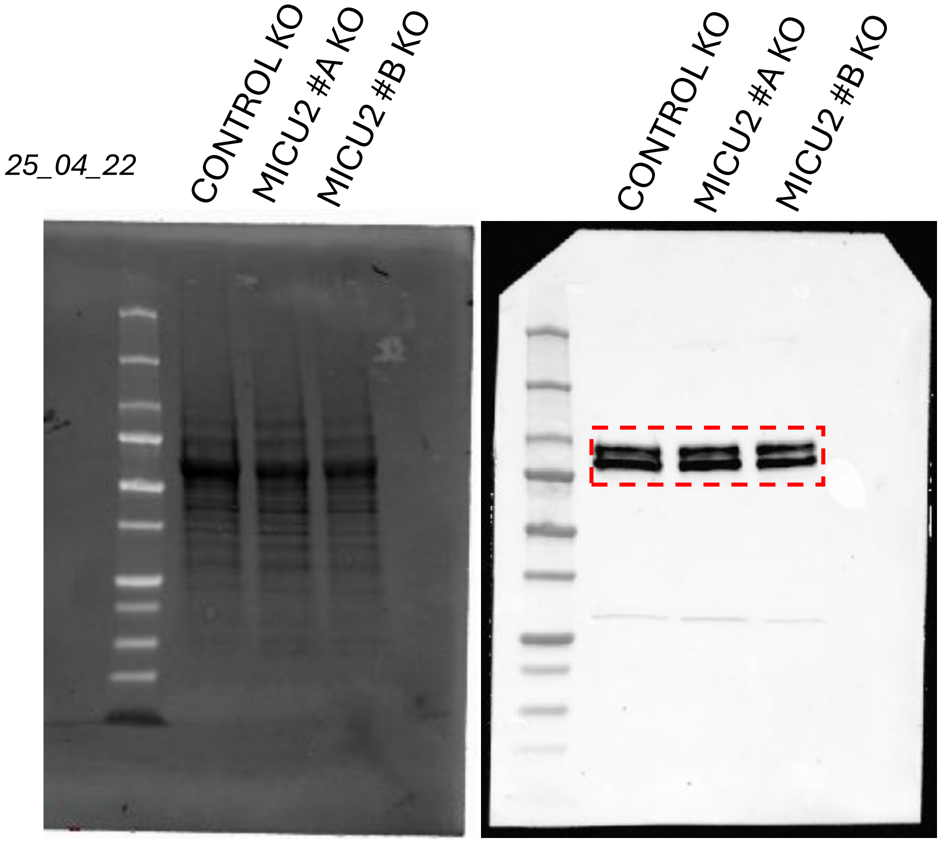

OPA1 (≈80 / 100kDa)

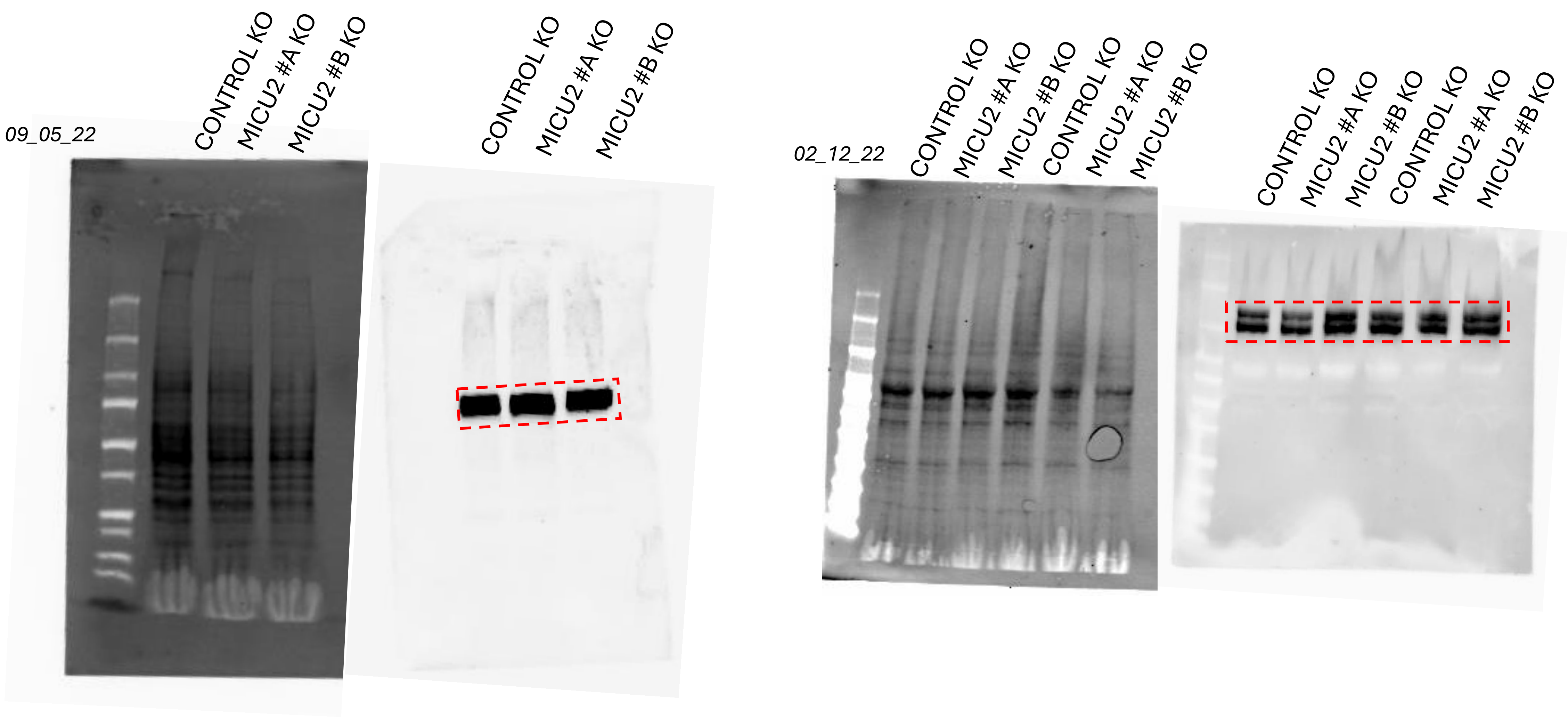

MFN2 (≈80kDa)

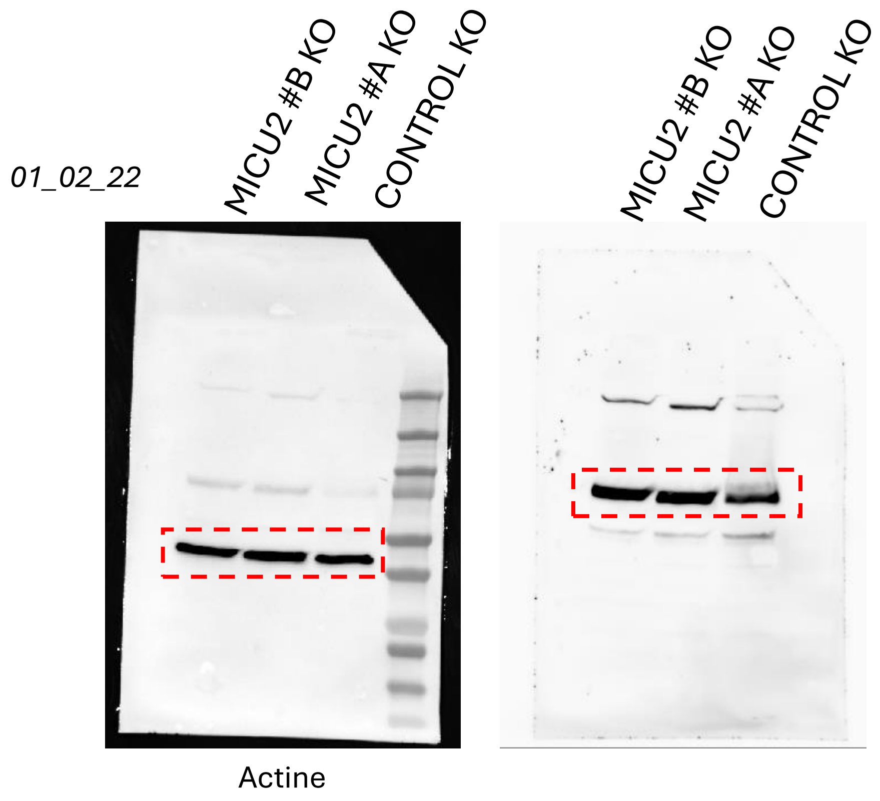
01_02_22
MICU2 #B KO
MICU2 #A KO
CONTROL KO
MICU2 #B KO
MICU2 #A KO
CONTROL KO
Actine

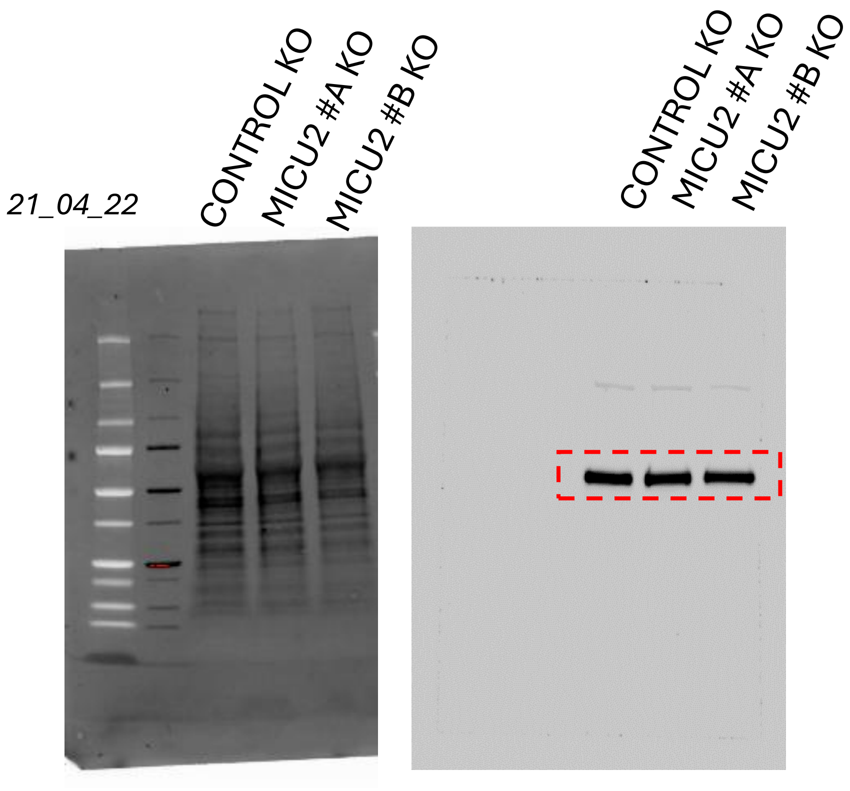
21_04_22
CONTROL KO
MICU2 #A KO
MICU2 #B KO
CONTROL KO
MICU2 #A KO
MICU2 #B KO

MFN2 (≈80kDa)

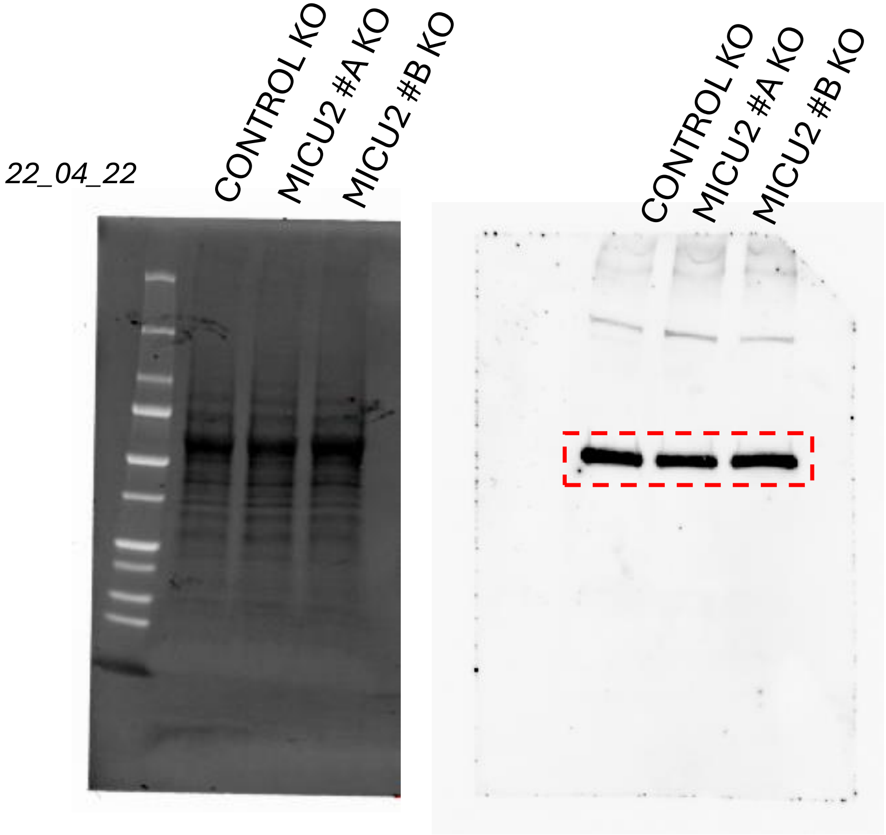

22_04_22
CONTROL KO
MICU2 #A KO
MICU2 #B KO
CONTROL KO
MICU2 #A KO
MICU2 #B KO

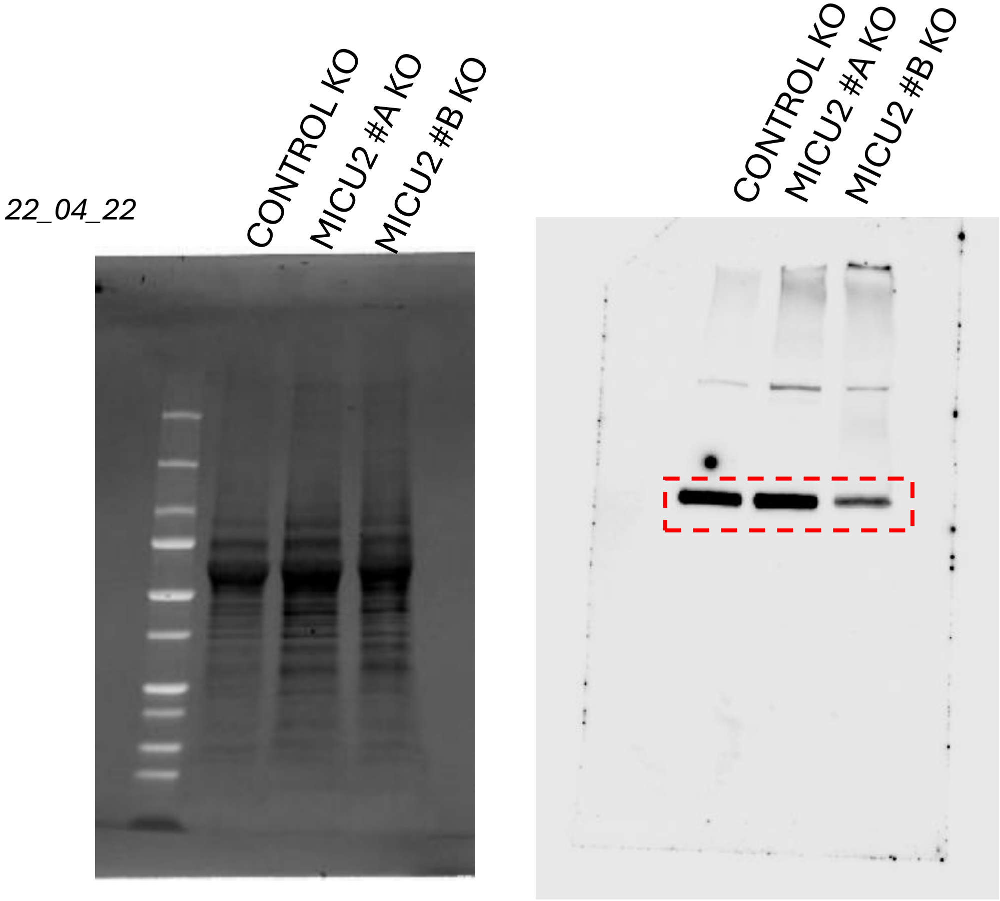

22_04_22
CONTROL KO
MICU2 #A KO
MICU2 #B KO
CONTROL KO
MICU2 #A KO
MICU2 #B KO

MFN2 (≈80kDa)

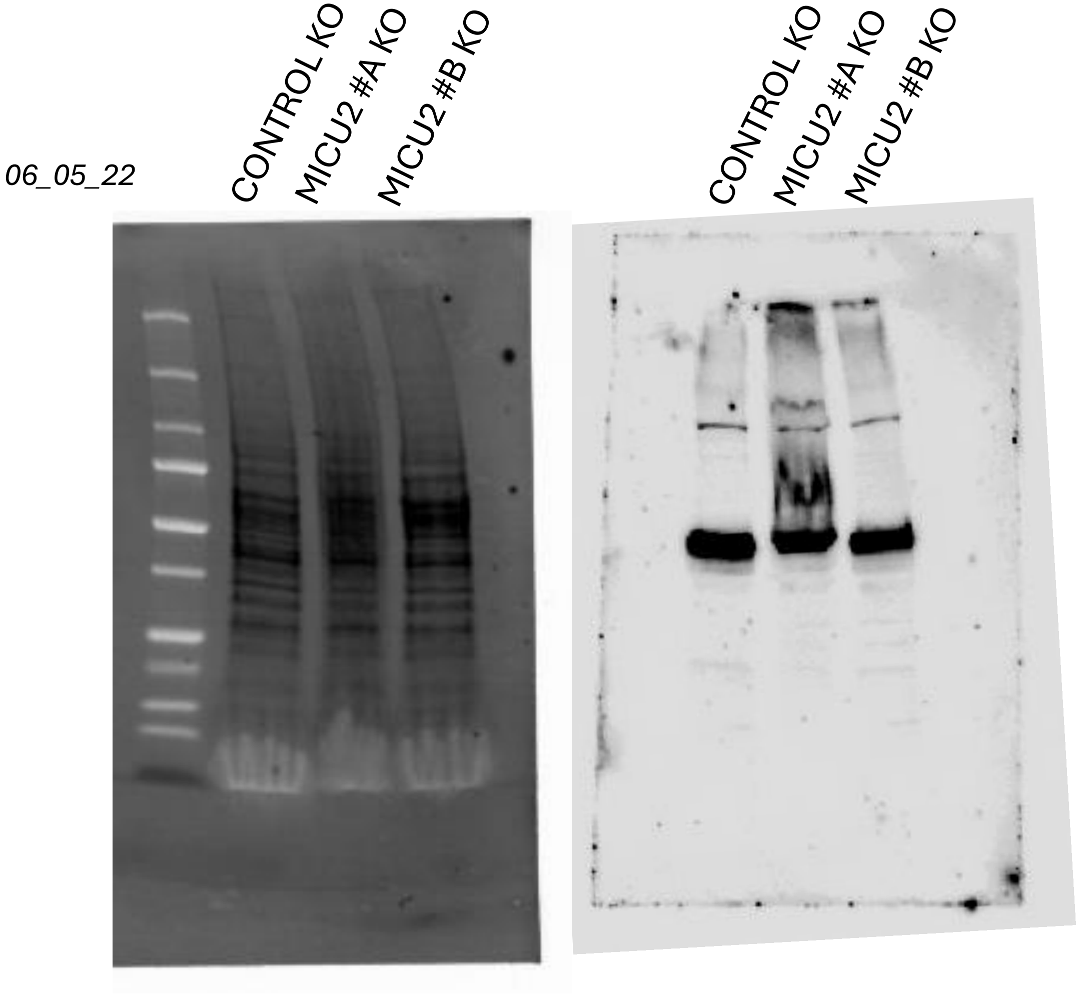

06_05_22
CONTROL KO
MICU2 #A KO
MICU2 #B KO
CONTROL KO
MICU2 #A KO
MICU2 #B KO

P-DRP1 (≈80kDa)

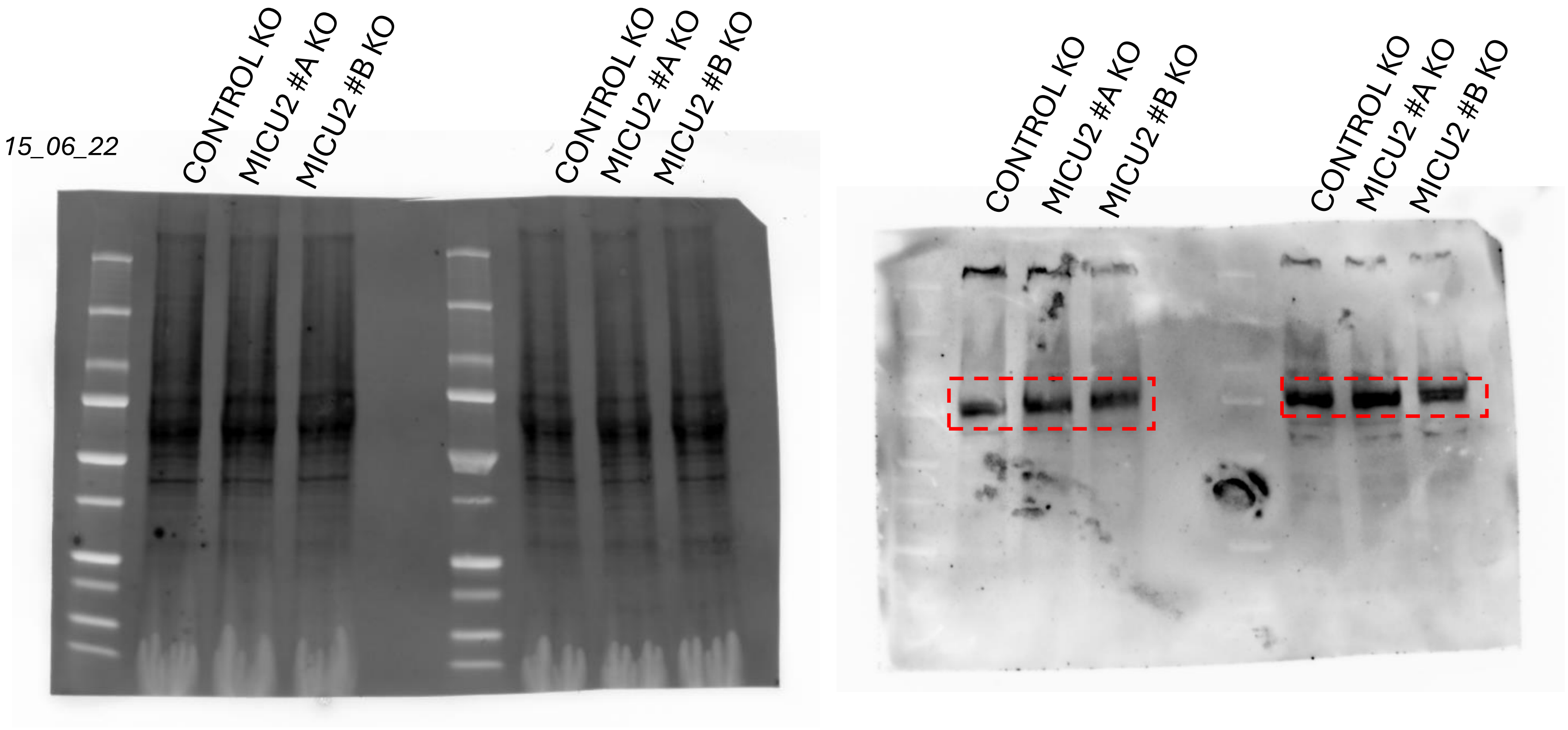
15_06_22
CONTROL KO
MICU2 #A KO
MICU2 #B KO
CONTROL KO
MICU2 #A KO
MICU2 #B KO
CONTROL KO
MICU2 #A KO
MICU2 #B KO
CONTROL KO
MICU2 #A KO
MICU2 #B KO

P-DRP1 (≈80kDa)

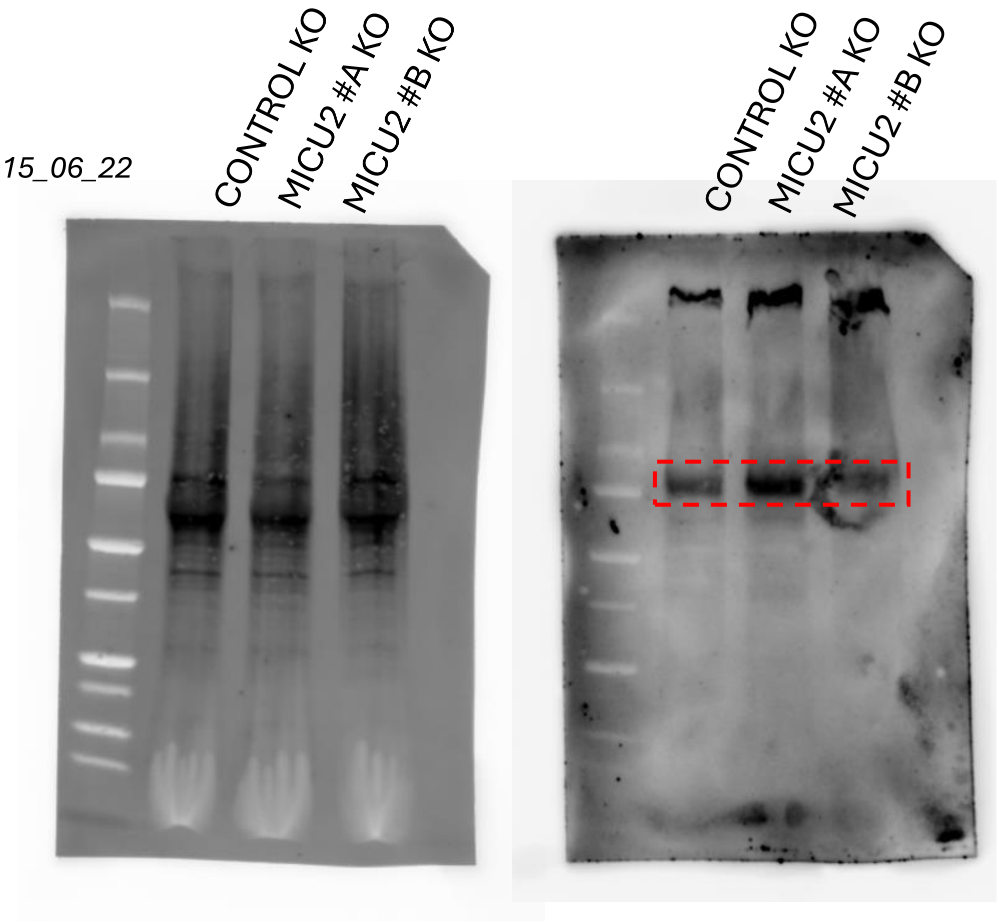

15_06_22
CONTROL KO
MICU2 #A KO
MICU2 #B KO
CONTROL KO
MICU2 #A KO
MICU2 #B KO

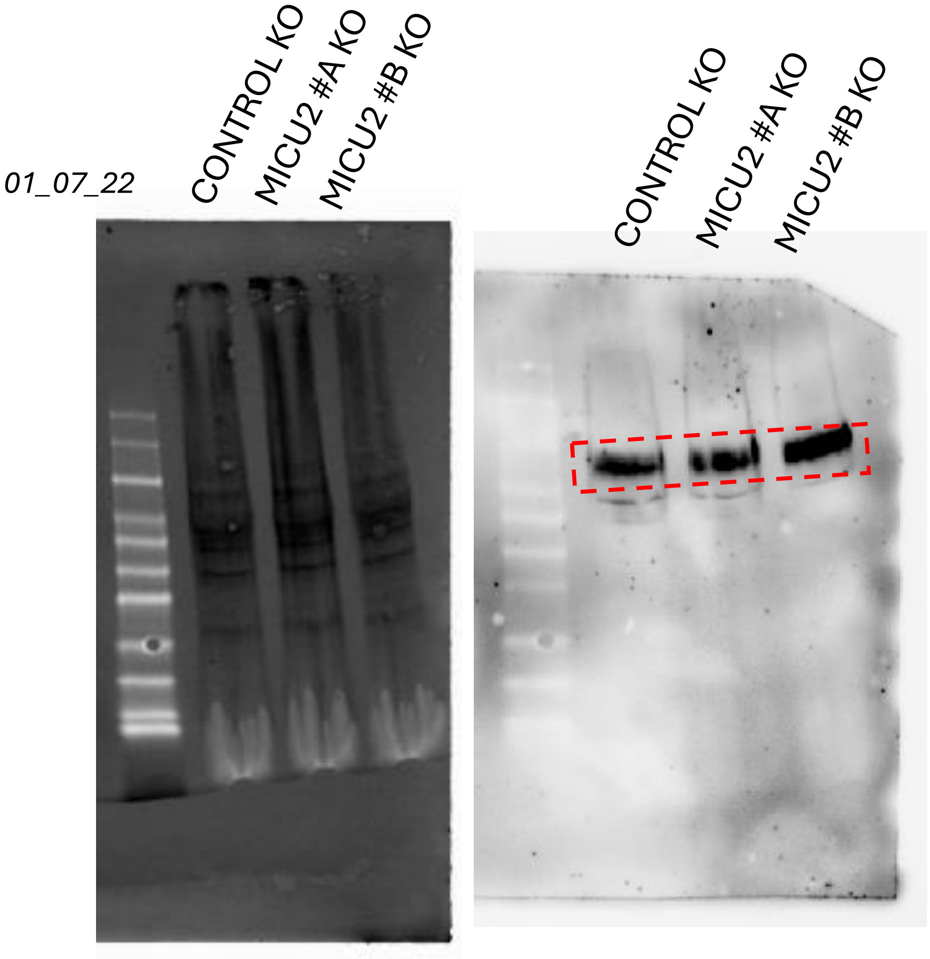

01_07_22
CONTROL KO
MICU2 #A KO
MICU2 #B KO
CONTROL KO
MICU2 #A KO
MICU2 #B KO

P-DRP1 (≈80kDa)

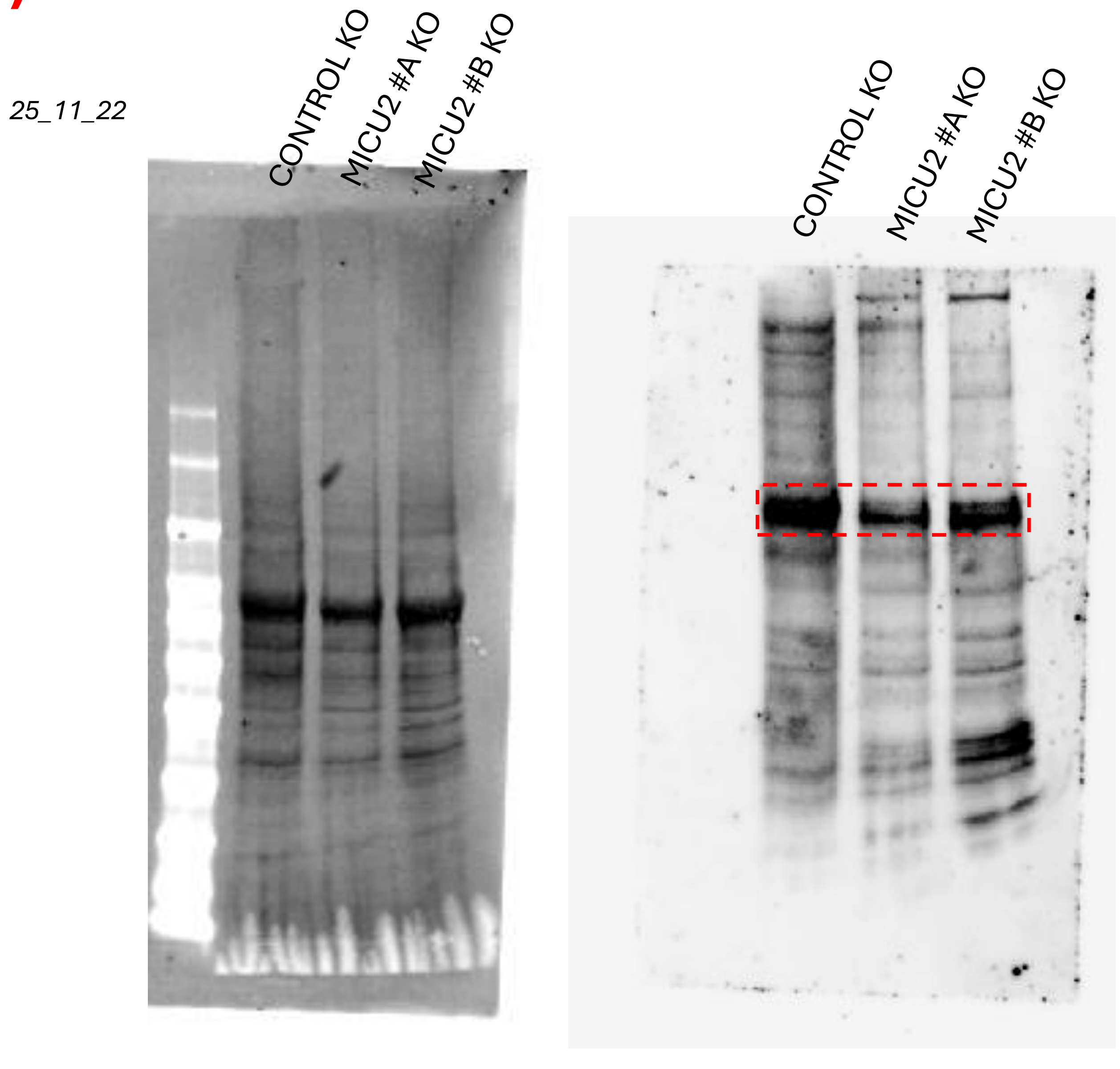
25_11_22
CONTROL KO
MICU2 #A KO
MICU2 #B KO
CONTROL KO
MICU2 #A KO
MICU2 #B KO

DRP1 (≈80kDa)

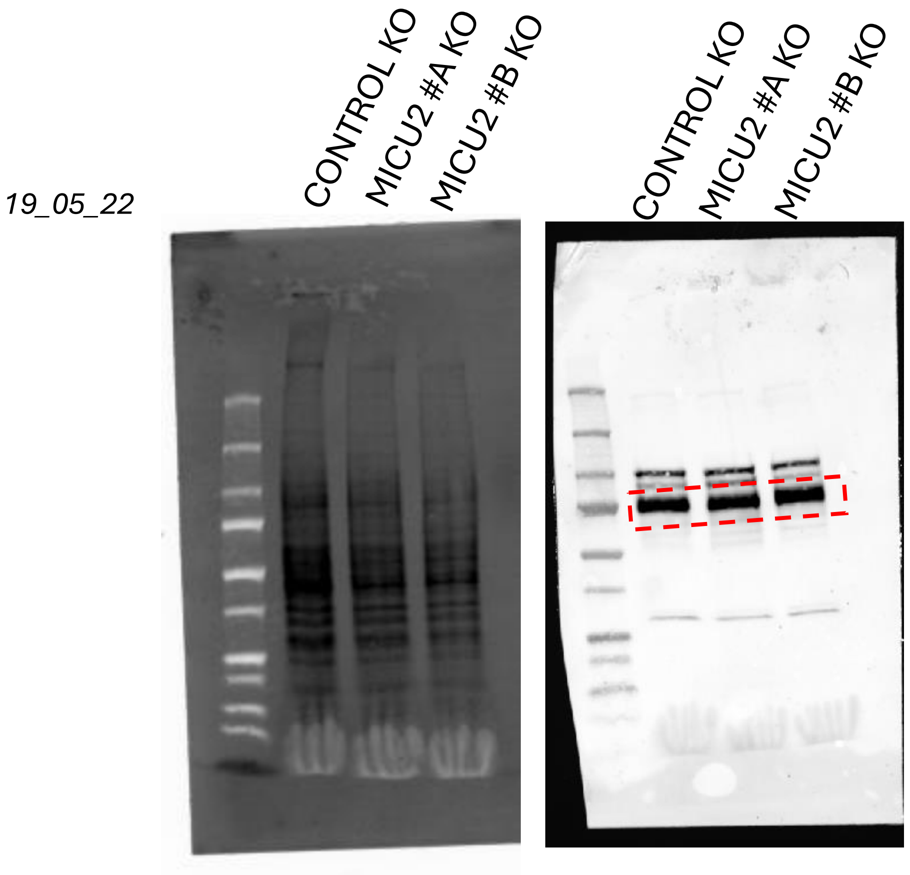
19_05_22
CONTROL KO
MICU2 #A KO
MICU2 #B KO
CONTROL KO
MICU2 #A KO
MICU2 #B KO

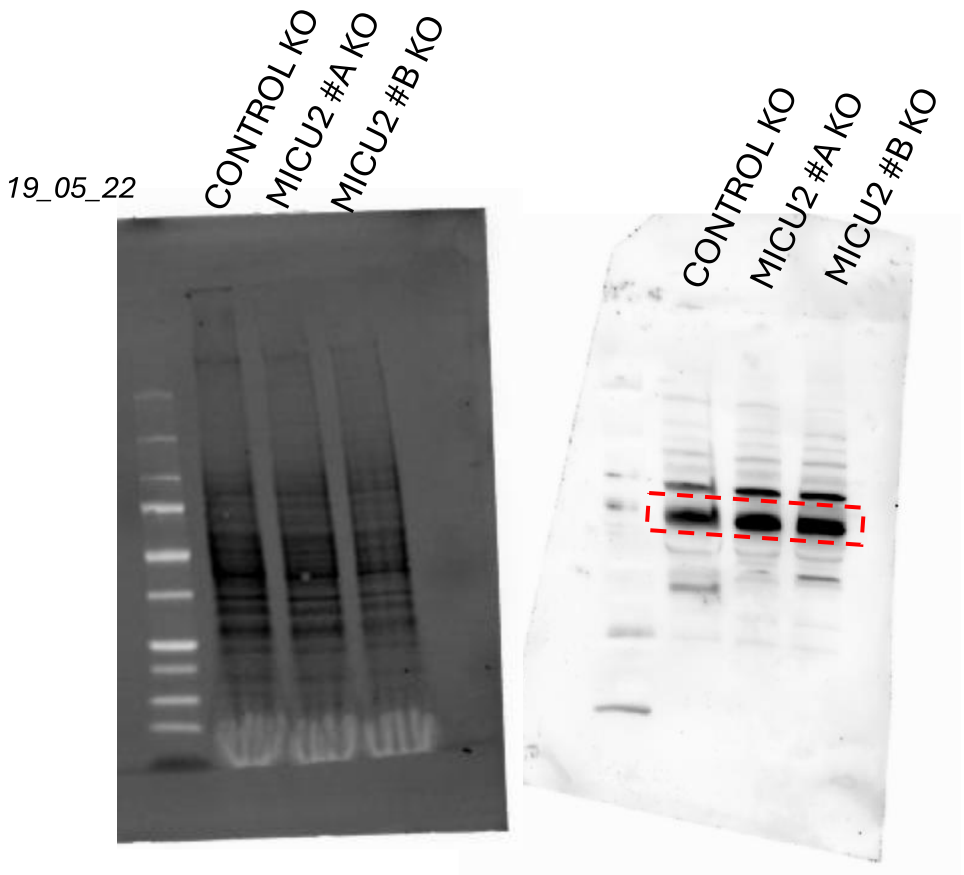
19_05_22
CONTROL KO
MICU2 #A KO
MICU2 #B KO
CONTROL KO
MICU2 #A KO
MICU2 #B KO

DRP1 (≈80kDa)

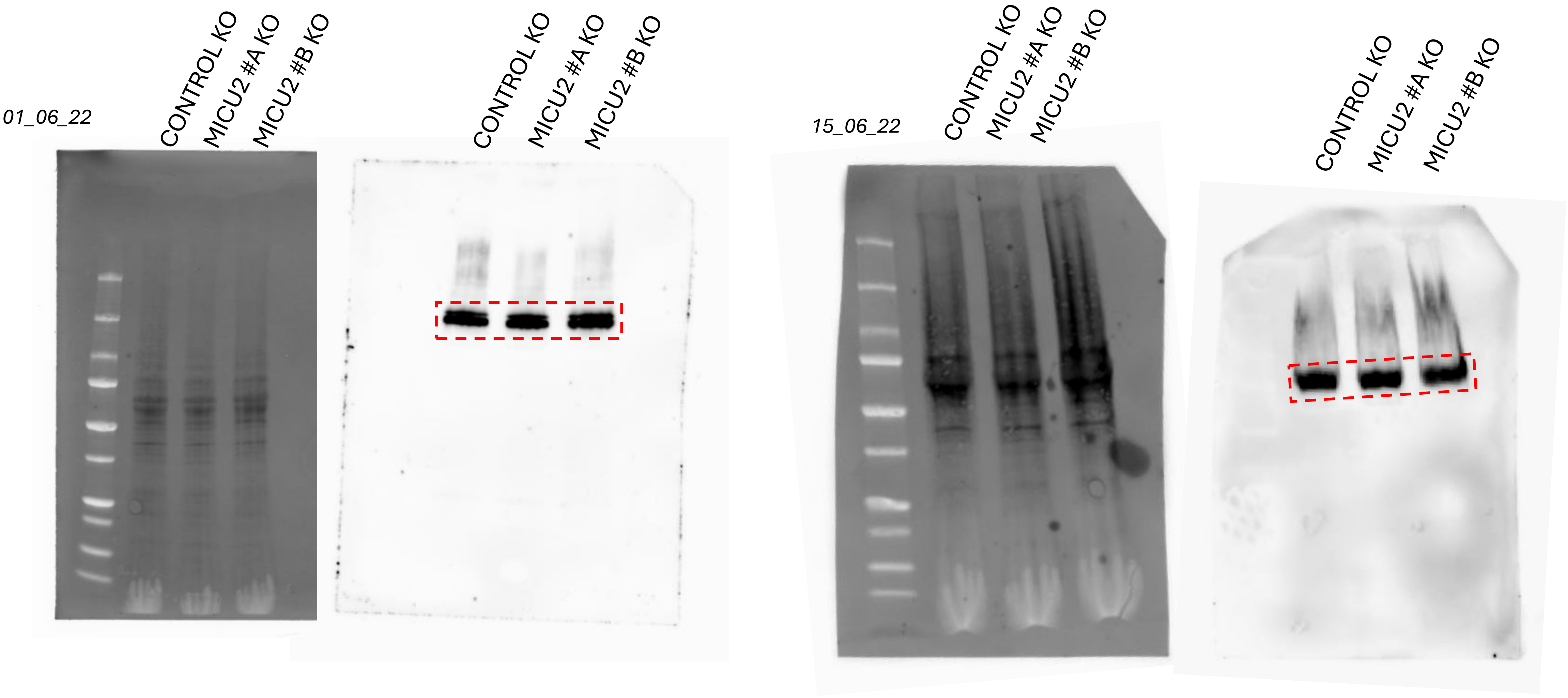
01_06_22
CONTROL KO
MICU2 #A KO
MICU2 #B KO
CONTROL KO
MICU2 #A KO
MICU2 #B KO
15_06_22
CONTROL KO
MICU2 #A KO
MICU2 #B KO
CONTROL KO
MICU2 #A KO
MICU2 #B KO

DRP1 (≈80kDa)

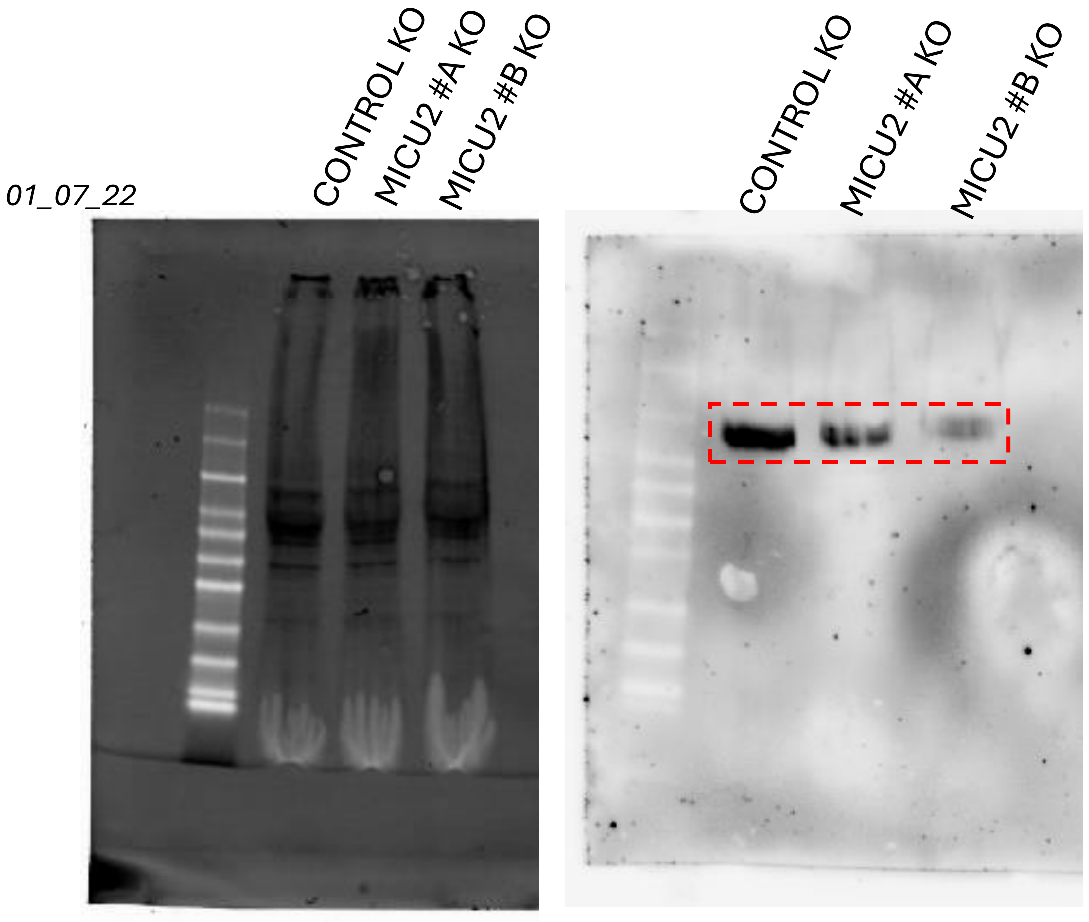
01_07_22
CONTROL KO
MICU2 #A KO
MICU2 #B KO
CONTROL KO
MICU2 #A KO
MICU2 #B KO

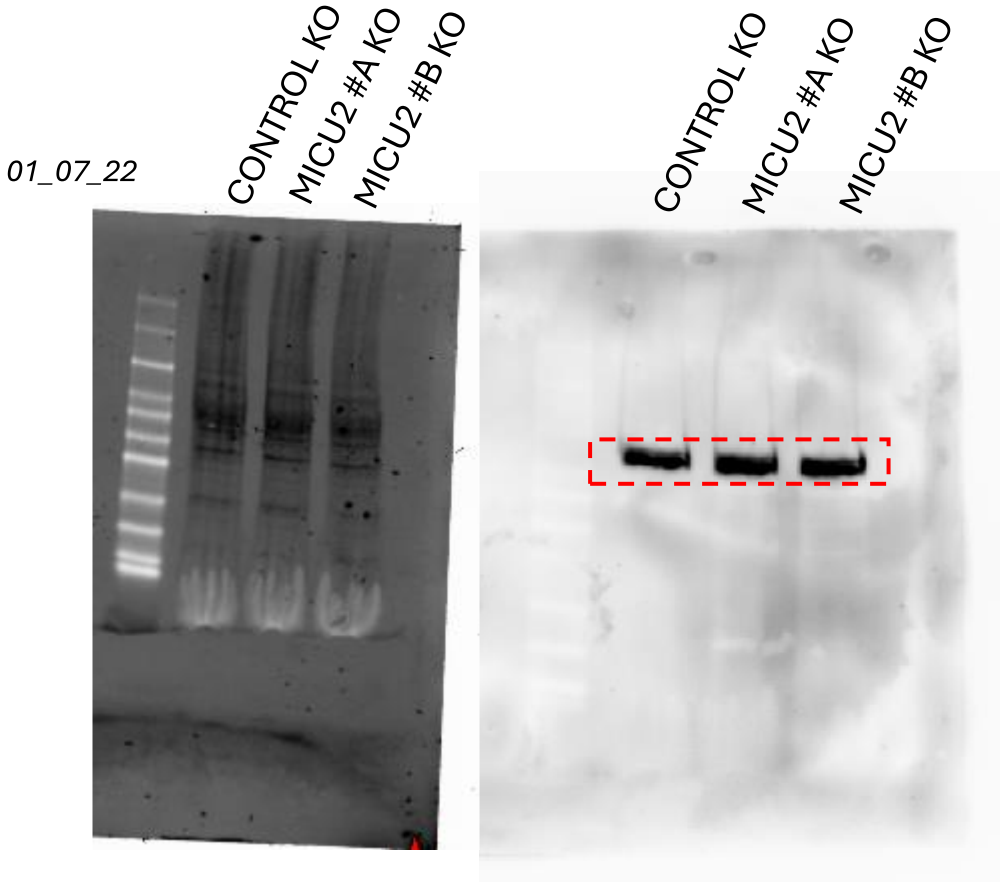
01_07_22
CONTROL KO
MICU2 #A KO
MICU2 #B KO
CONTROL KO
MICU2 #A KO
MICU2 #B KO

DRP1 (≈80kDa)

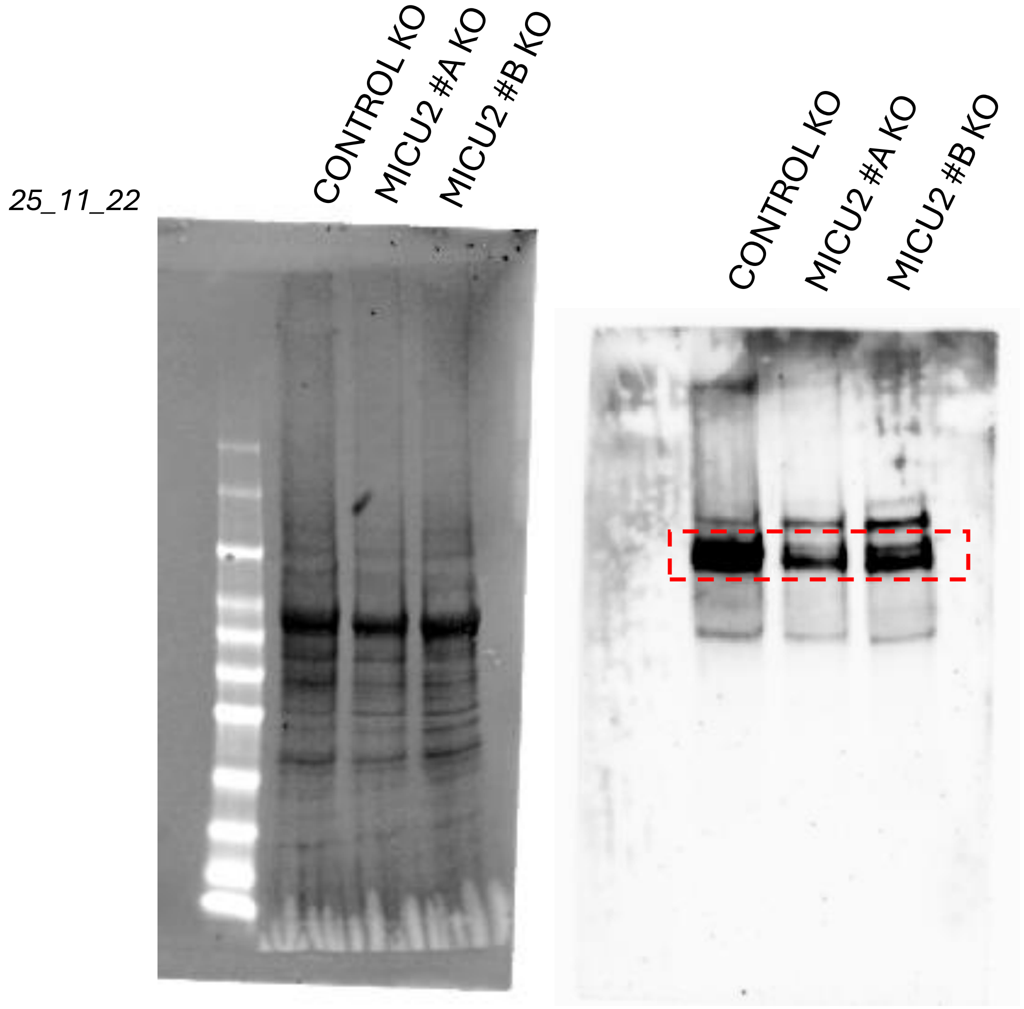
25_11_22
CONTROL KO
MICU2 #A KO
MICU2 #B KO
CONTROL KO
MICU2 #A KO
MICU2 #B KO

KO lines
Figure 4C

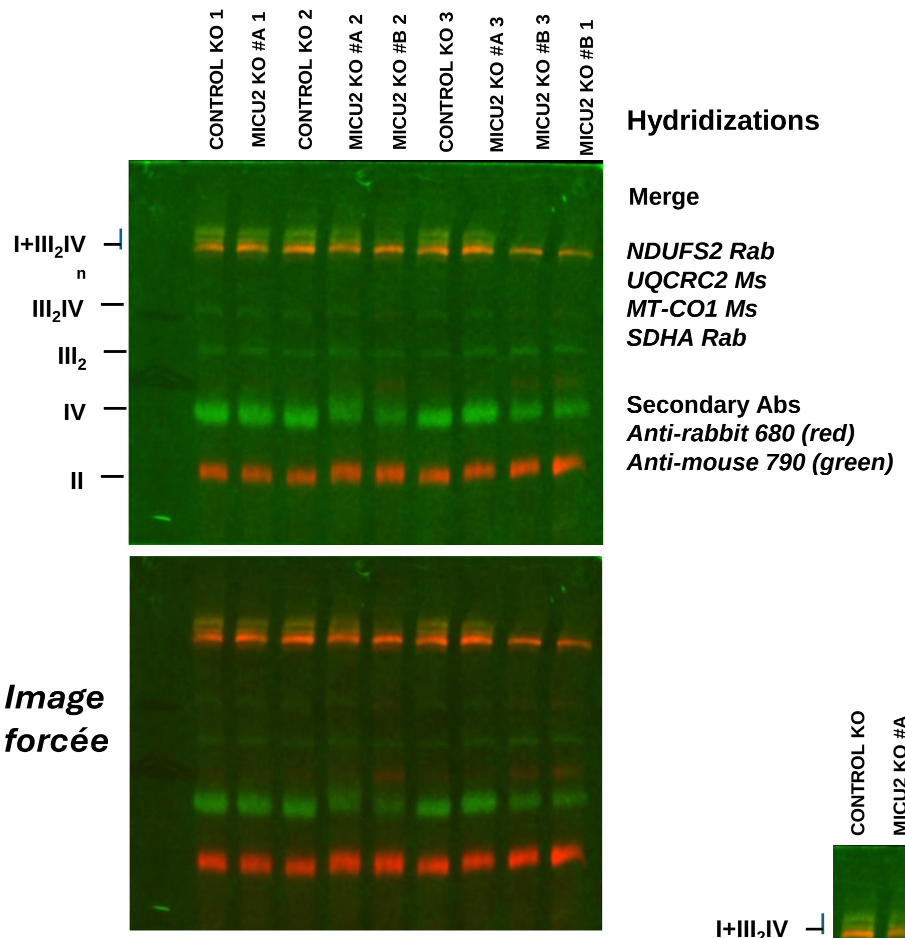
CONTROL KO 1
MICU2 KO #A 1
CONTROL KO 2
MICU2 KO #A 2
MICU2 KO #B 2
CONTROL KO 3
MICU2 KO #A 3
MICU2 KO #B 3
MICU2 KO #B 1
Hydridizations
Merge
NDUFS2 Rab
UQCRC2 Ms
MT-CO1 Ms
SDHA Rab
Secondary Abs
Anti-rabbit 680 (red)
Anti-mouse 790 (green)
I+III$_2$IV$_n$
III$_2$IV
III$_2$
IV
II
Image forcée

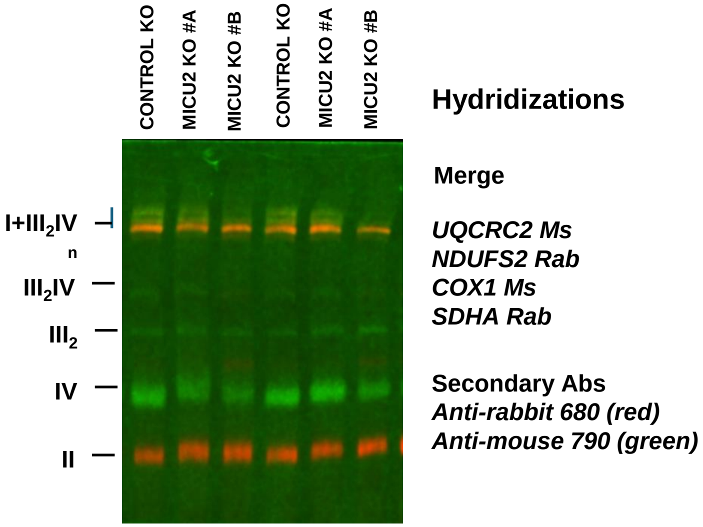
CONTROL KO
MICU2 KO #A
MICU2 KO #B
CONTROL KO
MICU2 KO #A
MICU2 KO #B
Hydridizations
Merge
UQCRC2 Ms
NDUFS2 Rab
COX1 Ms
SDHA Rab
Secondary Abs
Anti-rabbit 680 (red)
Anti-mouse 790 (green)
I+III$_2$IV$_n$
III$_2$IV
III$_2$
IV
II

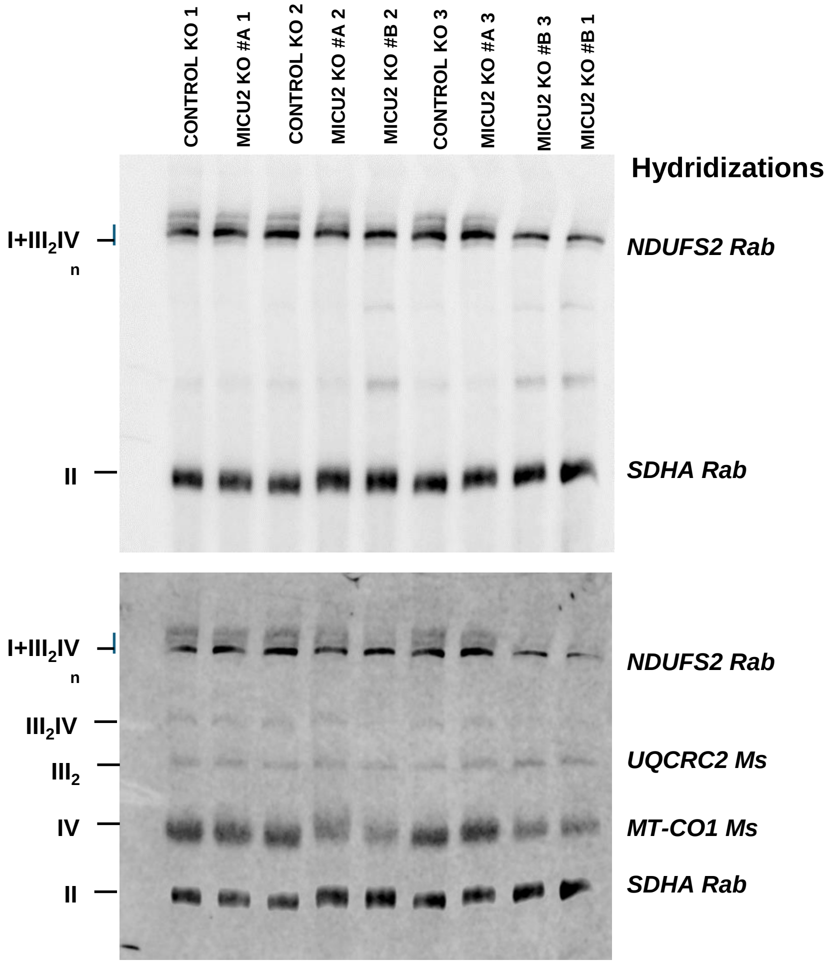
CONTROL KO 1
MICU2 KO #A 1
CONTROL KO 2
MICU2 KO #A 2
MICU2 KO #B 2
CONTROL KO 3
MICU2 KO #A 3
MICU2 KO #B 3
MICU2 KO #B 1
Hydridizations
NDUFS2 Rab
SDHA Rab
I+III$_2$IV$_n$
II
NDUFS2 Rab
UQCRC2 Ms
MT-CO1 Ms
SDHA Rab
I+III$_2$IV$_n$
III$_2$IV
III$_2$
IV
II

22/12/06

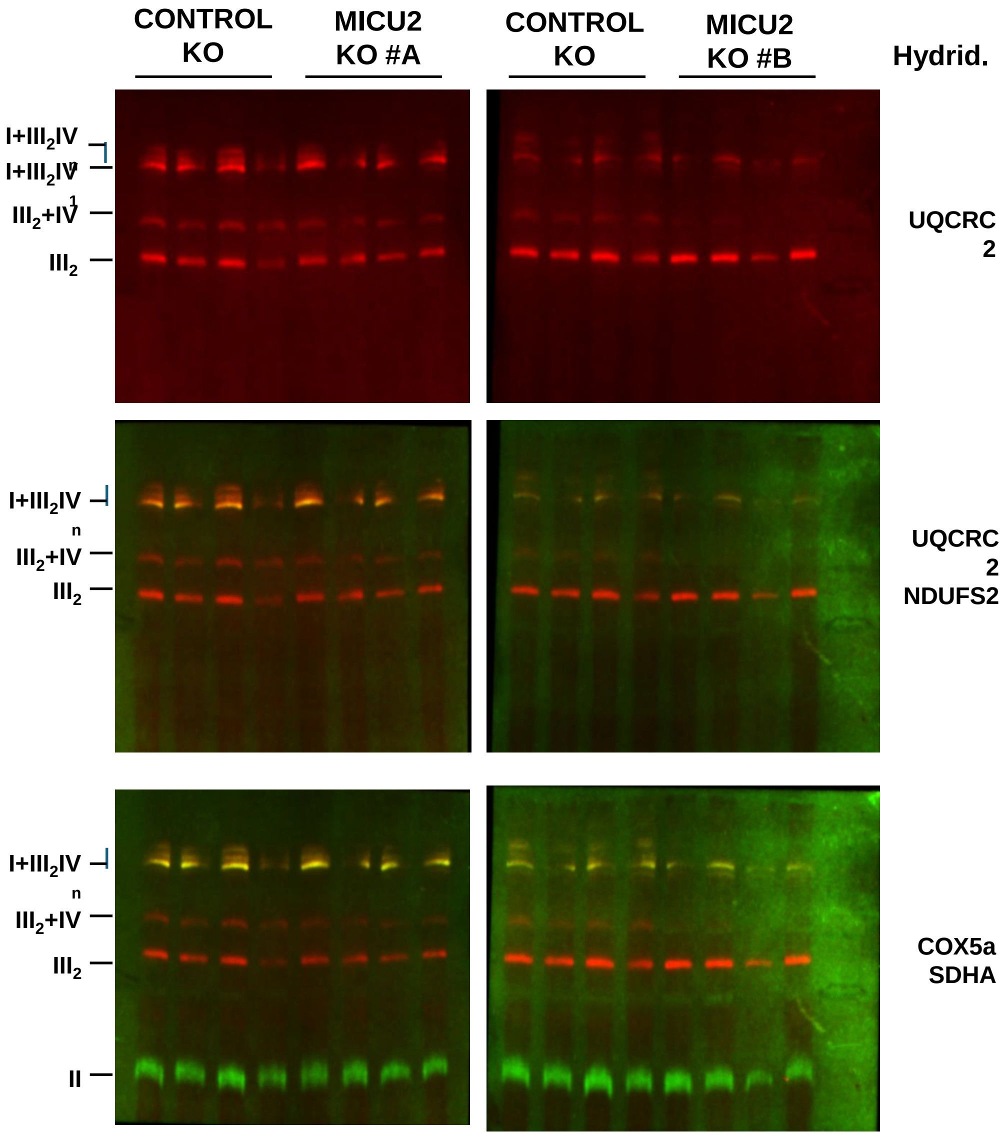

Secondaires :
Anti-Mouse 680 (red)
Anti-Rabbit 790 (green)

10/01/23

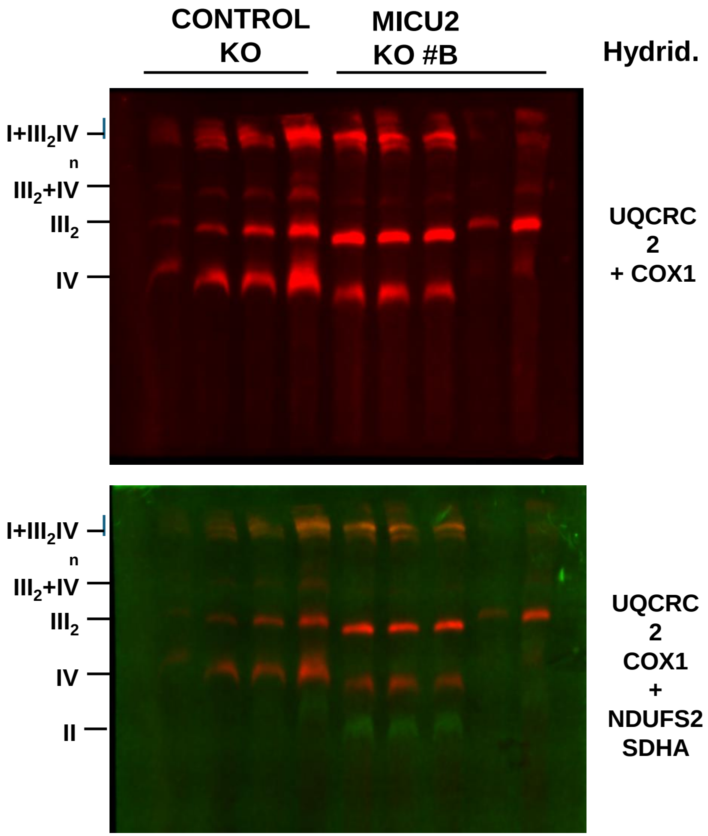

Secondaires :
Anti-Mouse 680 (red)
Anti-Rabbit 790 (green)

24/01/23

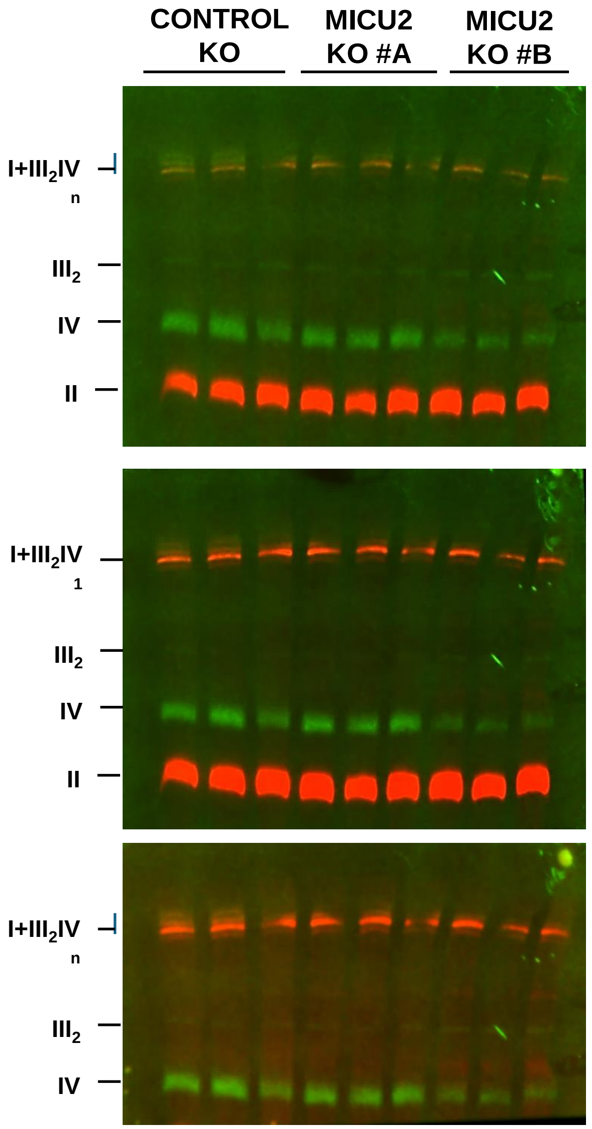

Hydridizations

UQCRC2 Ms
NDUFS2 Rab
COX1 Ms
SDHA Rab

Secondaires :
Anti-rabbit 680 (red)
Anti-mouse 790 (green)

Image forcée
Signal 680 (red)

La même, avec CII masqué

24/01/23

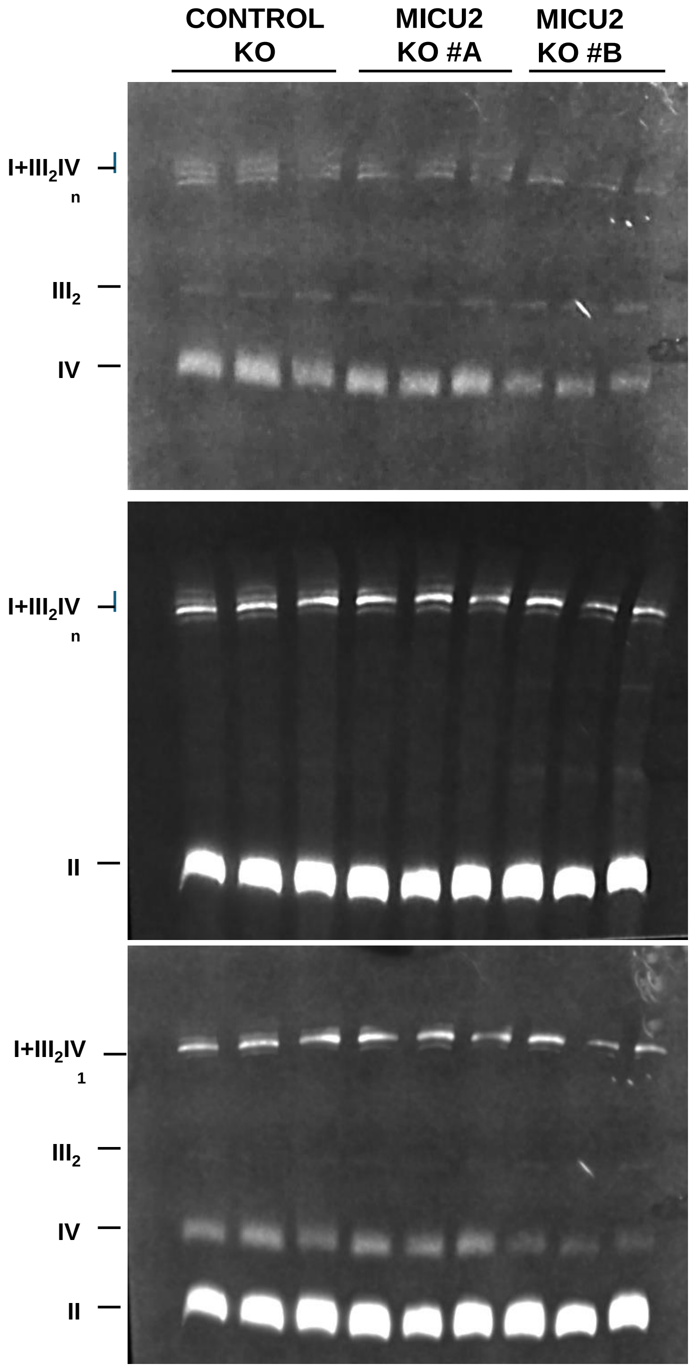

Hydridizations

Canal 790

UQCRC2 Ms
COX1 Ms

Canal 680

NDUFS2 Rab
SDHA Rab

Canaux 680/790

UQCRC2 Ms
NDUFS2 Rab
COX1 Ms
SDHA Rab

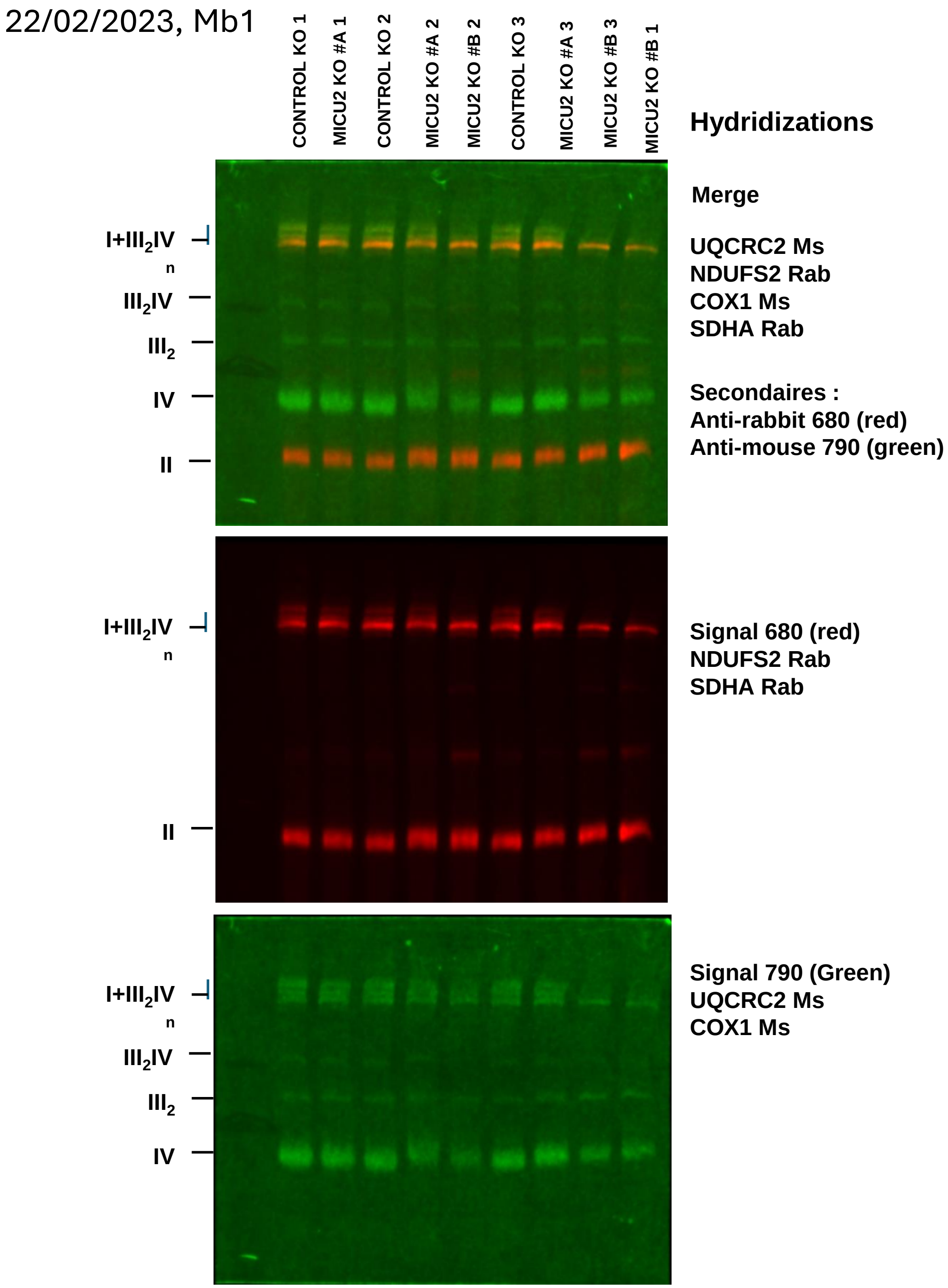
22/02/2023, Mb1
CONTROL KO 1
MICU2 KO #A 1
CONTROL KO 2
MICU2 KO #A 2
MICU2 KO #B 2
CONTROL KO 3
MICU2 KO #A 3
MICU2 KO #B 3
MICU2 KO #B 1
Hydridizations
Merge
UQCRC2 Ms
NDUFS2 Rab
COX1 Ms
SDHA Rab
Secondaires :
Anti-rabbit 680 (red)
Anti-mouse 790 (green)
I+III$_2$IV$_n$
III$_2$IV
III$_2$
IV
II
Signal 680 (red)
NDUFS2 Rab
SDHA Rab
I+III$_2$IV$_n$
II
Signal 790 (Green)
UQCRC2 Ms
COX1 Ms
I+III$_2$IV$_n$
III$_2$IV
III$_2$
IV

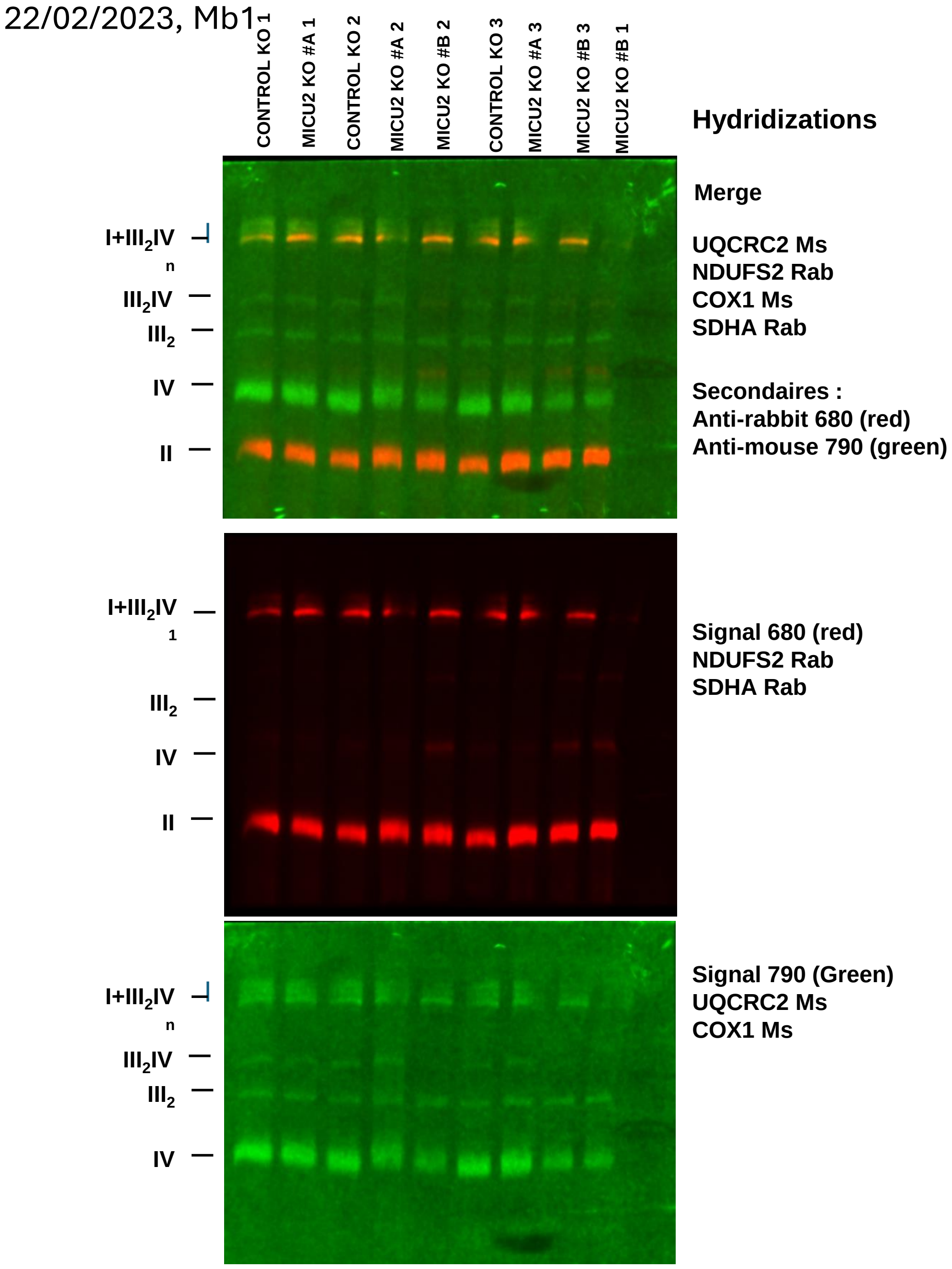
22/02/2023, Mb1
CONTROL KO 1
MICU2 KO #A 1
CONTROL KO 2
MICU2 KO #A 2
MICU2 KO #B 2
CONTROL KO 3
MICU2 KO #A 3
MICU2 KO #B 3
MICU2 KO #B 1
Hydridizations
Merge
UQCRC2 Ms
NDUFS2 Rab
COX1 Ms
SDHA Rab
Secondaires :
Anti-rabbit 680 (red)
Anti-mouse 790 (green)
I+III$_2$IV$_n$
III$_2$IV
III$_2$
IV
II
Signal 680 (red)
NDUFS2 Rab
SDHA Rab
I+III$_2$IV$_1$
III$_2$
IV
II
Signal 790 (Green)
UQCRC2 Ms
COX1 Ms
I+III$_2$IV$_n$
III$_2$IV
III$_2$
IV
