# Supplementary material for: MICU2 up-regulation enhances tumor aggressiveness and metabolic reprogramming during colorectal cancer development
Source: PLoS Biol. 2024 Oct 28;22(10):e3002854. doi: 10.1371/journal.pbio.3002854 (PMC11542858; doi:10.1371/journal.pbio.3002854)
Supplement: Attachment — Submitted filename: AnswerLetterPLOSBIOL_V5.docx [file pbio.3002854.s015.docx]

|  | |  |  | |
| --- | --- | --- | --- | --- |
|  |  | | |  |

July 9st, 2024

**Subject:** Revised Manuscript Submission for PLOS Biology: "Encoding of the Colorectal Cancer Metabolic Program through MICU2"

**Dear Ines Alvarez-Garcia, PhD,**

Thank you for the detailed feedback and for the opportunity to revise our manuscript entitled "Encoding of the colorectal cancer metabolic program through MICU2". We sincerely appreciate the time and effort that you, the Academic Editor, and the reviewers have dedicated to evaluating our work.

We have carefully considered the reviewers' comments and have undertaken additional experiments to address their concerns comprehensively. Our revised manuscript includes the following major updates:

1. **Reviewer 1's Comments:**
   - We have conducted experiments to show whether MICU2 depletion alters the size and formation of MCU.
   - Quantification has been added to several key experiments to provide clearer and more precise data.
   - The Methods section has been expanded for greater clarity and detail, and several figures have been improved for better presentation and interpretation of our data.
2. **Reviewer 2's Comments:**
   - We have analyzed the effect of MICU2 depletion on metastatic spread in vivo, adding new data to address this important aspect.
   - Clarifications and missing information noted by the reviewer have been incorporated into the text to improve the manuscript's comprehensiveness and readability.

We believe these revisions have significantly strengthened our manuscript and addressed the reviewers' concerns. We are hopeful that the updated version will meet the standards for publication in PLOS Biology.

We appreciate your patience during this revision process and apologize for any delays. We are committed to submitting the revised manuscript within the requested three-month timeframe.

Thank you once again for the constructive feedback and for considering our manuscript for publication. Please let us know if you require any further information.

Sincerely,

**Maxime GUEGUINOU (Ph.D)**

*Dr Maxime GUEGUINOU, PhD, CRCN INSERM, HDR*

*UMR INSERM 1069 Nutrition Croissance et Cancer (N2C)*

*Université de Tours, Faculté de médecine*

*10 Boulevard Tonnellé*

*37032 Tours, France*

*Tel : +33 (0)2.47.36.61.24*

[*maxime.gueguinou@univ-tours.fr*](mailto:maxime.gueguinou@univ-tours.fr)

**Reviewers' comments**

Rev. 1:

The manuscript concerns the mitochondrial consequences of loss of MICU2, part of the MCU. They investigate how this changes metabolism of the cell and some aspects of tumor phenotype. They show that loss of this protein leads to changes in mitochondrial fusion/fission, respiratory supercomplex expression, respiration (which impacts on central carbon metabolism more broadly) and some malignant phenotypes, such as invasion. It is an interesting study, which would benefit from a clearer link to tumors themselves - while small mRNA changes are observed, do these translate into protein changes, on which this manuscript is based?

We thank the expert for this comment. It's true that we are using bioinformatics to show the involvement of MICU2 in the development of Metastases in 2 cohorts of CRC patients, and these results have been supplemented by in vitro cell migration and invasion measurements on our cell lines. We wished to go further by studying the protein expression of MICU2 in TMA cohorts of patients, but unfortunately the specificity of the tissue markers was not good enough.

The development of primary culture or organoid CRC to measure both the expression of mitochondrial calcium players and their activities is under way, and will be the subject of an another publication.

To clarify and substantiate our transcriptomic findings, we complemented our transcriptomic analyses with an analysis of proteomic data generated by CPTAC conducted on the 95 tumor samples included into the TCGA-COAD and TCGA-READ genomic projects (Zhang et al. - Nature 2014, doi: 10.1038/nature13438).

Our proteomic analysis of 95 tumor samples reveals that among the MICU1/MICU2/EMRE MCU components, MICU2 tends to be over-expressed in stage 4 patients compared to those in stages 1 to 3 (P=0.063) in agreement with our findings on transcriptomic data. Furthermore, MICU2 is the only component significantly associated with vascular invasion (P=0.01) and shows a trend towards association with the presence of distant tumor metastases (P=0.077). Notably, high expression levels of MICU2 protein are significantly correlated with poorer patient survival (P=0.039).

Those data have been added in the manuscript (Supplemental Figure S1E).

Other comments:

1.The formation of the multiprotein complex MCU has been shown to depend on association of MICU1 and MICU2 as a heterodimer (Mallilankaraman et al 2012; Csordas et al 2013 and others). Given that the authors have shown that they are able to run native gels for supercomplexes, it would be important for this manuscript to show whether depletion of MICU2 alters the size and formation of MCU, given that MICU1 protein expression is unchanged (Figure 1D).

Thank you for the insightful comments regarding our research. We conducted an analysis of the MCU multiprotein complex using native gel electrophoresis, drawing inspiration from the methodologies outlined in Ghosh et al. (2022, DOI: 10.1093/hmg/ddab254) and Tsai et al. (2017, DOI: 10.1093/hmg/ddab254). We utilized validated antibodies for BN-PAGE, specifically those obtained from Cell Signaling (MCU, Cell Signaling 149997S, and MICU1, Cell Signaling 12524S).

Despite our efforts, the MICU1 antibody did not produce any results. However, we successfully validated the technique using the MCU antibody on HCT116 WT and MCU KO cell lines.

Interestingly, BN-PAGE analysis also reveals that the loss of MICU2 appears to partially alter the structure of the uniporter. This alteration is evident not around the 700 kDa structures but rather between the 300 and 450 kDa structures, where we observe an increased proportion of the 300 kDa form and a decreased amount of the 450 kDa form. Given the absence of differences around 700 kDa and the observed variations between 300 and 450 kDa, it is challenging to determine whether MICU2 loss leads to partial MICU1/MICU1 homodimerization or affects other proteins such as EMRE. Further studies will be necessary to uncover the structural changes in MCU in the absence of MICU2 in our model.

We hope this clarifies our findings and appreciate your continued guidance.

2. Figure 1C compares MICU1, 2 and MCU expression in cancer cell lines against a normal cell line. It would be best to include more than one representative normal cell line, as a single model is not enough to draw a conclusion regarding differences between normal and cancer. Additionally, it isn't clear that MICU1 or MCU are higher in the cancer cell lines than the normal - loading is not consistent between normal and the cancer cells, and given that western blotting is not fully quantitative, best practice would suggest quantification should really only be done on blots with equal loading.

We fully appreciate the reviewer's suggestion regarding the use of multiple normal cell lines. Unfortunately, no other human colonic epithelial cell lines are available on platforms such as ATCC. Concerning sample loading, we ensured that the same amount of protein was measured and loaded for each cell line. To avoid the variability associated with reference proteins across different cell types, we utilized the Stain-Free technique developed by Bio-Rad for normalization.

As stated on Bio-Rad's website, "Stain-Free imaging allows for the elimination of the inherently problematic use of housekeeping proteins as loading controls on western blots, enabling the user to obtain truly quantitative western blot data by normalizing bands to total protein in each lane." This approach allowed us to achieve accurate protein quantification.

Our findings regarding the increased expression of MCU in tumor cell lines align with the study by Liu et al. (Liu, Y., Jin, M., Wang, Y. et al. MCU-induced mitochondrial calcium uptake promotes mitochondrial biogenesis and colorectal cancer growth. Sig Transduct Target Ther 5, 59 (2020). <https://doi.org/10.1038/s41392-020-0155-5>), which demonstrated increased MCU expression in 203 patients through immunohistochemistry (IHC) in a matched comparison of tumor versus normal tissues.

3. In Figure 2C, this reviewer is not aware that it is possible to separate G0 from G1, or G2 from M through PI fluorescence by flow cytometry. If this is the case, could the authors outline in the materials and methods how this was performed, and if not, could they collapse the categories into broader cell cycle phases.

Thank you for your valuable comment; it's indeed crucial that we clarify our methodological approach. The different phases were separated using the following method: [10.1371/journal.pone.0068425](https://doi.org/10.1371/journal.pone.0068425)

Separation of G0 from G1 or G2 from M was achieved using the anti-Ki67 alexa fluor 488 antibody, which separates cells into cycles (G1, S, G2 and M). We separate M and G2 using the anti phospho-histone H3 alexa fluor 488 antibody, which separates cells in mitosis (M) from cells in other phases (G0, G1, S and G2). Finally, 7AAD enables us to separate cells with 2n chromosomes (G0/G1) from cells with 4n (G2/M). It is the combination of these markers that enables us to separate all phases of the cycle, even though we use two markers on the same fluorescence channel (anti Ki67 alexa fluor 488 and anti phospho-histone H3 alexa fluor 488 antibodies), as cells labelled with these antibodies are separable via their DNA content (7AAD labelling). We have corrected the material and method in the revised manuscript.

4. The results arising from Ki67 staining of tumors shown in Figure 2F were unexpected, in that the two knockdown constructs appeared to behave in different ways. Additionally, the pictures appear to be at different magnifications, despite the scale bar suggesting otherwise. The control KO punctae for Ki67 nuclear staining are significantly smaller than those for the MICU #B KO picture. In engraftment delay is indeed the reason for a change in proliferation, then the authors would need to take tumors of similar size (independent of engraftment) and examine these to compare like with like.

**CONTROL KO**

**MICU2 #A KO**

**MICU2 #B KO**

**1mm**

**1mm**

**1mm**

Thank you for your insightful feedback regarding the Ki67 staining results in Figure 2F. We appreciate your attention to detail and have several points to address based on your observations.

We apologize for any confusion regarding the magnification and scale bars in the images. It is crucial for our figures to be accurately represented, and we re-examined the images to ensure that all are presented at a consistent magnification. We corrected any inconsistencies and provide revised images to accurately reflect the comparative sizes of the stained nuclei. As suggested by the reviewer, we have taken particular care to select tumors that are relatively close in size for comparison. These images therefore replace Figure 2F.

5. Figure 4B and S4A and B. The authors suggest that their analysis of the TCGA-COAD data shows that MICU1 high tumors have higher expression of genes associated with the assembly of the complexes and go onto show changes in some supercomplex formation. Could the authors consider also showing protein expression of the assembly factors to support the RNA changes observed in tumor samples?

We express our gratitude to the reviewer for this pertinent comment, but after analyzing MICU2 expression in the 95 samples from the TCGA library run through proteomics, we noticed that the MICU2/MICU1 ratio emerged as irrelevant since protein expression is undetectable for MICU1 in most samples. At the protein level, we found a link between the respiratosome and MICU2 expression.

6. At the end before the section of the results describing Figure 4, the authors state that 'overall, we demonstrated that MICU2, unlike MICU1, controls the complex biogenesis…' While in this manuscript they do show that depletion of MICU2 alters supercomplex expression in cells, they do not show that depletion of MICU1 does not. They would need to refer to previously published work here to be able to make that comparison.

We thank the reviewer for his comments. In our study, we indeed observed a change in supercomplex expression in cells with MICU2 deletion. We concluded that MICU1 does not play a role in the biogenesis of the complex, based on this study, as it does not appear to have any influence when present. There is no work on MICU1 deletion to support this conclusion. Consequently, we will modify the manuscript in the revised version.

7. In Figure 5D the authors present mRNA for the PLINs. The stability of PLIN2 expression is controlled post-translationally, so it would be more informative to show protein expression.

In response to the reviewer's comment, we employed confocal microscopy to quantify perilipin using the fluorescent Perilipin antibody from Thermo Fisher (validated in the lab for the identification of lipids droplets in adipocytes). Regrettably, as evidenced by the images below, we were not able to detect perilipin proteins (double stained by anti-rabbit Alexa-Fluor 594, in red on the images and Nucleus DAPI In Blue) suggesting that in our conditions, the levels of perilipin is too low to be detected by our antibody. Therefore, we have opted to rely on the PCR data and appropriately qualify our statements in the article.

You'll find the images below.

Image showing perilipin labelling in red and the dapi nucleus in blue in the HCT116 cell line.

8. In Figure 6, the authors note that tumor oxygenation is lower in MICU2 KO cell clone A-derived tumors (6G). This is unexpected, given that for a given tumor size, these cells use less oxygen, and therefore the diffusion gradients across the tumor are likely to be less steep, leading to relatively higher oxygenation - similar to that seen after atovaquone treatment in a number of other studies. The increased glycolysis cannot be used as an indicator of a preference for growing in hypoxia, and this preference will not change the environment - the environment is create due to a combination of supply and demand for oxygen. As the authors have shown that there is increased hypoxia, and that the tumor cells use less oxygen, the supply must be significantly less in these tumors - this will be important to look for, or an alternative explanation.

We would like to thank the expert for their insightful comment. Indeed, it is conceivable that tumor metabolism, particularly anaerobic glycolysis, could influence tumor vascularization in breast and Colorectal cancer, potentially leading to variations in tumor oxygenation. Cancer Res. 2011 Apr 1;71(7):2550-60. doi: 10.1158/0008-5472.CAN-10-2828. Epub 2011 Feb 7.

Therefore, we cannot exclude a potential role for MICU2 in tumor angiogenesis and vessel quality. Future experiments will be necessary to determine whether the presence of MICU2 is an important factor in tumor vessel quality, potentially explaining the differences in oxygenation between WT and MICU2 KO tumors.

Discussion about these particularly intriguing results have been added to the discussion section.

Minor comments:

1.In Figure 2C, the colors are a bit hard to differentiate between, particularly the orange plot. Could the authors add the percentage cells in each phase of the cell cycle to the plot?

Thank you for this helpful comment on our article's message. The percentage of cells in each cycle phase has been added in the revised manuscript. We have also changed the colors of the graphs to make it easier to understand the different phases.

Rev. 2:

The paper by Robert et al. explores the role of MICU2, and EF-hand containing regulator of the mitochondrial calcium uniporter MCU in the development and metabolic reprogramming of colon carcinoma. The authors used a vast variety of techniques in combination with bioiformatics in order to prove their hypotheses. The work is performed carefully, however a number of points require clarification.

Introduction:

Please specify which isoform of VDAC has been linked to calcium transport across the OMM.

Several studies have consistently demonstrated that Voltage-Dependent Anion Channel isoform 1 (VDAC1) is the predominant isoform found in many malignant tumors, including colorectal cancer (CRC). Due to its prevalence and potential significance in cancer biology, we have chosen to target this specific isoform in our research efforts. This decision is supported by the substantial body of literature indicating that VDAC1 may play crucial roles in tumorigenesis and could be a promising therapeutic target in CRC and other cancers.

Please specify whether Garg et al reported activation of MCU by both MICU1 and MICU2. If yes, it is difficult to understand why expression of MICU1 and MICU2 are respectively down- and upregulated with stages of CRC, as cited in the results section. In addition, upregulation of MICU2 would counteract the Warburg effect/lactate production according to the graphical abstract based on the results. Is it known from the literature if CRC cells tend to suppress the Warburg effect with stage advancement and metastatic spread? MICU2 KO cells, in which more lactate is produced, gives rise to smaller tumors. This is somewhat unexpected, please give possible explanation. Is it possible that more lactate does not aid tumor growth because the experiments were performed in NOD-SCID mice where lactate probably does not remodel the tumor microenvironment?

Decades ago, Otto Warburg proposed that tumor cells exhibit higher glycolytic activity compared to normal cells, suggesting mitochondrial damage and downregulation of oxidative phosphorylation (OXPHOS) in tumors. However, recent experimental evidence has contradicted this assumption by demonstrating that mitochondrial respiratory function remains intact in most tumor cells. In fact, OXPHOS activity can be upregulated in certain cancers, playing a crucial role in cell proliferation and metastasis.

The study by Chekulayev et al. (2015, <https://doi.org/10.1016/j.bbrep.2015.08.020>) investigated glycolytic reprogramming in adjacent cells of colorectal cancer (CRC). Postoperative analyses compared OXPHOS and glycolytic activity between CRC cells and healthy colon tissue cells, revealing higher OXPHOS rates in both CRC cells and surrounding cells compared to healthy tissue. This finding suggests that CRC is not primarily characterized by acute hypoxia.

Additionally, the study highlighted increased glucose-phosphorylating capacity and upregulation of VEGF in CRC, further supporting the absence of acute hypoxia in CRC. Notably, CRC growth was associated with reduced creatine kinase activity and increased OXPHOS in surrounding tissue, indicating significant metabolic alterations during CRC progression.

Two other studies provide further insights into the role of OXPHOS in CRC:

- Kaldma et al. (2014) conducted an in situ study demonstrating that the OXPHOS system is a major provider of ATP in colorectal cancer cells. They found that inhibition of OXPHOS can suppress CRC proliferation and metastasis.
- Huang et al. (2019) identified LYRM2 as a regulator of complex I activity, supporting tumor growth in colorectal cancer through oxidative phosphorylation.

Together, these studies underscore the role of OXPHOS in CRC proliferation and metastasis.

The function of MICU subunits is to enhance the activity of the MCU complex as cytosolic Ca2+ levels rise and bind to the EF hands of MICU proteins. This enhancement leads to efficient stimulation of mitochondrial ATP production in response to cytosolic Ca2+ signaling events, particularly during periods of increased energy demand, as demonstrated in neurons (Ashrafi et al., 2020) and the heart (Wescott et al., 2019).

Referring back to the study by Matesanz-Isabel et al. (2016, BBA), their work indicates the following:

MICU1 and MICU2, the two main regulators of the mitochondrial Ca2+ uniporter (MCU), have distinct and defined functions.

Both MICU1 and MICU2 inhibit MCU at low cytosolic Ca2+ concentrations ([Ca2+]c), but these inhibitory effects are additive and kinetically different.

MICU1 activates MCU at high [Ca2+]c (> 2.5 μM), whereas MICU2 has no effect at high [Ca2+]c.

These findings underscore the complex regulatory roles of MICU1 and MICU2 in modulating MCU activity in response to varying Ca2+ levels, thereby influencing mitochondrial function and cellular energy metabolism

The author write: "So far, the contribution of MICU2 to the regulation of mitoCa2+ signaling, bioenergetics, metabolic reprogramming, mitochondrial dynamics, and CRC development have not been addressed."

There are several studies addressing the role of MICU2 in the regulation of mitochondrial calcium signaling, dynamics and metabolic reprograming. Please modify the sentence accordingly and cite those studies.

There are indeed studies on the role of MICU2 in the regulation of mitochondrial calcium signaling, dynamics and metabolic reprograming, but these studies do not focus exclusively on CRC. Moreover, some authors study MICU2 by deleting MICU1, which does not reflect the specific role of MICU2.

Results:

In addition to reference 12, please cite the earlier papers where the effects of MICU1 and MICU2 have been studied on calcium flux into the mitochondrial matrix (works by the groups of Mootha, Hajnoczky, Rizzuto).

As suggested by the reviewer, several previous papers (reference 12) have studied the effects of MICU1 and MICU2 on calcium flux in the mitochondrial matrix, which reinforces the message of our work.

The papers included in the revised manuscript are:

1. Cell Metabolism 17, 976–987, June 4, 2013 ª2013 Elsevier Inc. MICU1 Controls Both the Threshold and Cooperative Activation of the Mitochondrial Ca2+ Uniporter Gyorgy Csordas, Tunde Golenar, Erin L. Seifert, Kimberli J. Kamer, Yasemin
2. Paillard M, Csordás G, Szanda G, Golenár T, Debattisti V, Bartok A, Wang N, Moffat C, Seifert EL, Spät A, Hajnóczky G. Tissue-Specific Mitochondrial Decoding of Cytoplasmic Ca2+ Signals Is Controlled by the Stoichiometry of MICU1/2 and MCU. Cell Rep. 2017 Mar 7;18(10):2291-2300. doi: 10.1016/j.celrep.2017.02.032. PMID: 28273446; PMCID: PMC5760244.
3. Kimberli J Kamer, Zenon Grabarek, and Vamsi K Mootha. High‐affinity cooperative Ca2+ binding by MICU1–MICU2 serves as an on–off switch for the uniporter. EMBO rep (2017) 18: 1397 – 1411.
4. MICU1 and MICU2 Finely Tune the Mitochondrial Ca2+ Uniporter by Exerting Opposite Effects on MCU Activity, Patron et al 2014.

"Significantly, MCU, MICU1 and MCUb were negatively expressed while MICU2 was positively expressed". How can be a protein negatively expressed? Maybe the authors refer to a lower expression compared to healthy tissues."

Thank you for your comment, we did indeed misspeak. We have modified our comments in the revised manuscript.

Figure 1C: The authors show an increased expression of MCU, MICU1 and MICU2 with increasing aggressiveness. However, at the beginning of the result section they write "In particular, using The Cancer Genome Atlas Colon Adenocarcinoma (TCGA-COAD) dataset, the authors observed that the expression of MICU1 and MICU2 are respectively down- and upregulated with stages". Please explain.

We understand the comment made by reviewer 2. To clarify these points, it should be noted that Figure 1B is based solely on transcriptomic data comparing stages 1 to 3 vs. stage 4. These results show that at the transcriptomic level, MICU2 appears to be the only component increased in metastatic patients.

Figure 1C shows the protein expression of MCU, MICU1, and MICU2 in five colonic cancer cell lines and one normal epithelial cell line. The protein results indicate that MCU is more highly expressed in the tumor cell lines than in the normal line. This trend also appears for MICU1 and MICU2 expression in the SW480, HCT116, and SW620 cell lines, which are more likely to migrate and invade in vitro assays and produce more metastases in preclinical mouse models.

However, in contrast to the large number of transcriptomic samples, we have a limited number of cell lines. We used the proteomic results from 95 patients in the COAD TCGA cohort, as mentioned in our response to reviewer 1 at the beginning of this letter. Proteomic analysis of MICU2 shows that only MICU2 expression impacts patient survival and is the only component that tends to be significant both in terms of expression in stage 4 and in metastatic concealment, despite the restricted sample size.

Taken together, these transcriptomic and proteomic data suggest a predominant role for MICU2 in colorectal cancer aggressiveness. However, the role of MCU shown in our analyses seems somewhat at odds with the data from Liu, Y., Jin, M., Wang, Y. et al. (2020). "MCU-induced mitochondrial calcium uptake promotes mitochondrial biogenesis and colorectal cancer growth." Signal Transduction and Targeted Therapy, 5, 59. <https://doi.org/10.1038/s41392-020-0155-5>.

BAPTA-AM seems to decrease viability regardless of the presence of MICU2 and the conclusion of the authors from this experiment is not clear.

Thank you for your valuable comment. We have clarified our conclusions in the paper. To investigate whether the presence of MICU2 could alter the buffering capacity of cytosolic calcium, potentially affecting functions such as cell proliferation or migration, we studied the impact of cytosolic calcium reduction using BAPTA-AM. Our results demonstrate that the presence of BAPTA-AM reduces the viability of both WT and MICU2 KO colonic cells. This suggests that the presence or absence of MICU2 does not play a significant role in controlling cytosolic calcium concentration during proliferative activity.

Since MICU2 KO lines show decreased migration, on the other hand MICU2 is highly expressed in metastatic cells, it would be crucial to understand the effect of the lack of MICU2 in metastatic spread in vivo. This would significantly strengthen the paper and link the observations in preclinical models with clinical data.

Please write out PRPS1.

B

A

**Fig:A** In vivo imaging analysis of tumor progression in a liver metastatic mouse model was performed by intrasplenic injection of HCT116 WT or MICU2 KO cells on days 7, 14, 21 and 28, . The quantification of the total flux luminescence in mice, as measured by IVIS at different time points, is shown on the right (n=5 to 8).

**Fig:B** Six representative images show the metastatic liver in a mouse model 33 days after injection.

We thank the reviewer for this comment. We wanted to address the question using a liver metastasis model to better match the MICU2 expression observed in the patients in Figure 1, where the transcriptomic data show high MICU2 expression in liver metastases from colorectal cancer.

We addes these results and this text to follow the paper.

Colorectal cancer (CRC) metastatic dissemination to the liver is one of the most life-threatening malignancies in humans and represents the leading cause of CRC-related mortality. Forced metastasis models are performed by injecting HCT116 WT or MICU2 KO cells into the spleen. To prevent primary tumor growth in the spleen, a hemi-splenectomy is performed 15 minutes after injection. Using an in vivo imaging system, we observed a decrease in tumor burden induced by MICU2 KO cells compared to control cells in nude mice at different time points. Additionally, the number of metastatic nodules was significantly higher in mice injected with HCT116 WT cells than in those injected with HCT116 MICU2 KO cells.

Discussion:

Please discuss whether the known chemical MICU/MCU modulators might be useful to decrease tumor growth in light of your findings.

The reviewer's comment is highly relevant when considering the need to mimic the consequences of MICU2 KO at the mitochondrial calcium level and potentially elucidate the different phenotypic effects observed. It would be intriguing to employ MCU blockers such as mitoxantrone or Benzothenium chloride, or MCU/MICU1 modulators like the MCU-I4 or MCU-I11 inhibitors developed by Di Marco et al. (2020). However, it is important to note that no pharmacological inhibitor has been developed to date that can prevent the activation and conformational change of MICU2. Exploring this avenue could offer significant potential for reducing tumor proliferation and metastatic spread in colorectal cancer (CRC).

Materials and methods:

The methodologies used to obtain Figures 2A and 6J are not sufficiently described. Please specify the program used to perform these analyses.

Thank you for your comment. We have modified our manuscript.
